# Holistic concept guided quality control of traditional Chinese medicines for optimizing bioavailability

**DOI:** 10.1186/s13020-025-01269-w

**Published:** 2025-11-25

**Authors:** Yawen Tian, Juan Yao, Qian Gao, Linwei Zou, Pengxiang Lu, Tiantian Guan, Xuefeng Liu, Shuangyan Zhou, Xiaojie Jin

**Affiliations:** 1 College of Pharmacy, Gansu University of Chinese Medicine, Lanzhou, 730000 China; 2 Key Laboratory of Dunhuang Medicine, Ministry of Education, Gansu University of Chinese Medicine, Lanzhou, 730000 China; 3 Gansu Phamaceutical Industry Innovation Research Institute, Gansu University of Chinese Medicine, Lanzhou, 730000 China; 4 Chongqing Key Laboratory of Big Data for Bio Intelligence, Chongqing University of Posts and Telecommunications, Chongqing, China

**Keywords:** Quality control, Bioavailability, Traditional Chinese medicines, Safety, Efficacy, Holistic concept

## Abstract

Traditional Chinese medicines (TCM) exerts multiple pharmacological effects, including anti-inflammation, antioxidation, and immune regulation, etc. However, its clinical efficacy and safety depend not only on the synergy of active substances but also on the precise control of potential toxic constituents. Therefore, a clear understanding of the in vivo behavior of TCM is essential. Bioavailability, as a key indicator for evaluating the clinical efficacy and safety of TCM, are increasingly recognized as interdependent. The ultimate goal of quality control is reflected in the bioavailability observed in clinical practice, making it a critical link for ensuring consistency and safety of TCM. This review summarizes recent progress on the mechanisms and roles of bioavailability in TCM quality control, and highlights that overcoming current bottlenecks requires cross-disciplinary integration. Guided by TCM theory and a holistic perspective, quality control should focus on biological effects rather than fragmented component analysis, and shift toward systematic and integrative approaches. Adhering to these principles is fundamental for improving bioavailability, enhancing clinical potential, and ensuring the safe use of TCM.

**Supplementary Information:**

The online version contains supplementary material available at 10.1186/s13020-025-01269-w.

## Introduction

Traditional Chinese medicines (TCM) has been shown to exert a wide range of therapeutic effects, including anti-inflammatory [1], antioxidant [2] and immunomodulatory [3] activities, etc. The integration of modern biomedical technologies has further improved the scientific understanding of these effects. With the growing clinical value and wider application of TCM products, increasing attention has been paid worldwide to their mechanistic studies, efficacy evaluation, and quality control [4]. Nevertheless, significant challenges remain and should not be overlooked.

However, a fundamental gap persists in current TCM quality control: the predominant reliance on monitoring a limited set of chemical components as markers. While quality control frameworks have been established across the entire process from raw materials to finished TCM products, these approaches largely fail to capture the holistic nature and pharmacodynamic (PD) basis of TCM therapeutics, creating a significant disconnect between traditional practice and modern pharmaceutical standards [5]. It is widely recognized that the therapeutic advantages of formulas arise from their complex multi-component synergies and multi-target mechanisms, thereby producing holistic effects. However, the conventional component-based marker approach is inherently inadequate for evaluating this complexity and predicting clinical outcomes. Traditional quality control methods primarily monitor changes in a few key components, but fundamentally lack the capability to assess the actual in vivo bioavailability and bioactivity that ultimately determine therapeutic efficacy and safety [6]. At the same time, non-standardized cultivation and processing [7] excreate safety inconsistencies. These limitations, particularly the disconnect between static component markers and dynamic bioactivity, constitute a major bottleneck. They hinder reliable efficacy and safety assessments and limit broader clinical adoption compared to conventional drugs.

It is worth noting that bioavailability research, by dynamically characterizing the in vivo fate and systemic exposure of complex TCM components, has become a pivotal bridge for evaluating the clinical efficacy and safety of TCM in line with its holistic nature [8]. More importantly, bioavailability and quality control are increasingly recognized as interdependent. On one hand, the effectiveness of quality control is ultimately reflected in the bioavailability observed in clinical practice; on the other hand, optimization of bioavailability relies on improved quality control strategies [9]. This reciprocal relationship directly addresses the limitations of the few key component-centric approach and underscores the critical importance of bioavailability in several key aspects: (1) bioavailability studies reveal the absorption, distribution, metabolism, and excretion (ADME) of both bioactive and potentially toxic constituents within the complex TCM matrix. This provides crucial insights into how systemic exposure patterns are linked to therapeutic and safety outcomes, moving decisively beyond the static evaluation of a few marker components towards a functional understanding [10]; (2) bioavailability research clarifies how multiple factors—including interactions among constituents, dosage form design, and preparation methods—influence the absorption and systemic exposure of key bioactive fractions or the overall formula profile. Such knowledge supports the development of quality control strategies explicitly designed to enhance bioavailability and ensure consistent therapeutic effects and safety [11]; (3) by correlating systemic exposure profiles with clinical responses, bioavailability studies enable more accurate evaluation of efficacy and safety in real-world settings. This also provides a foundation for guiding personalized medicine approaches in Traditional Chinese Medicine [12].

Breaking through the bottleneck of quality control in TCM requires cross-disciplinary integration, linking quality evaluation with clinical application in line with the holistic concept of Traditional Chinese Medicine. Recent advances highlight how multidisciplinary approaches can systematically investigate bioavailability [13]. Metabolomics uncovers metabolic pathways of compounds and builds correlations between constituents and efficacy [14]. Network pharmacology and quality marker (Q-marker) studies further clarify pharmacological mechanisms and active constituents [15]. Nanotechnology, targeted delivery systems, and molecular compatibility strategies address absorption challenges of active constituents [16–18]. Artificial intelligence (AI), especially machine learning (ML) and deep learning (DL), integrates with these technologies to analyze massive TCM datasets, supporting key tasks such as material identification, active ingredient discovery, and process optimization [19]. Together, these innovations help pave the way for reconciling holistic evaluation with standardization in quality control, facilitating the essential paradigm shift from static, component-based analysis to dynamic, bioeffect-oriented assessment.

Based on the above issues, this review provides a novel perspective by positioning bioavailability as the central pillar for advancing TCM quality control. It critically examines the current status and challenges of integrating bioavailability concepts into TCM quality control. The paper systematically discusses the technical methods used to detect and evaluate bioavailability, and analyzes how innovative quality control measures can be designed to specifically improve bioavailability, thereby enhancing clinical safety and effectiveness. The overarching aim is to synthesize current knowledge and propose a bioavailability-oriented framework for modern TCM quality control, capable of ensuring predictable clinical efficacy and controllable quality risks by bridging the critical gap between chemical composition and biological response.

## Study on bioavailability of TCM

### ADME mechanism in bioavailability of TCM

#### ADME as a determinant of TCM exposure and efficacy

The clinical outcomes of TCM, including therapeutic efficacy and safety, are closely associated with the systemic exposure of its bioactive constituents and potential risk substances. This holistic relationship is dynamically reflected through the ADME process. Therefore, investigating the ADME and bioavailability of TCM provides a fundamental basis for understanding and managing potential risks in clinical use.

Absorption, the physiological process by which drugs enter systemic circulation from their administration sites, constitutes the initial phase of drug disposition. In TCM, oral administration predominates, with absorption mainly occurring in the gastrointestinal tract [8]. The Caco-2 cell model is widely used in vitro to evaluate intestinal drug absorption, with the apparent permeability coefficient (Papp) serving as the key parameter. According to established criteria, Papp values < 1.0 × 10^−6^cm/s indicate poor absorption, 1.0 × 10^−6^ ~ 1.0 × 10^−5^ cm/s suggest moderate absorption, and values > 1.0 × 10^−5^ cm/s indicate favorable absorption potential [20]. This model quantifies net permeation reflecting the combined effects of multiple transport pathways, although it has limited capacity to capture the complex multi-component interactions characteristic of TCM. For example, gastrodia elata polysaccharides exhibited a Papp of (1.29 ± 0.08) × 10^−^⁶cm/s, indicating moderate intestinal absorption, consistent with macropinocytosis and clathrin-mediated uptake of large hydrophilic molecules [21].

**Fig. 1:**
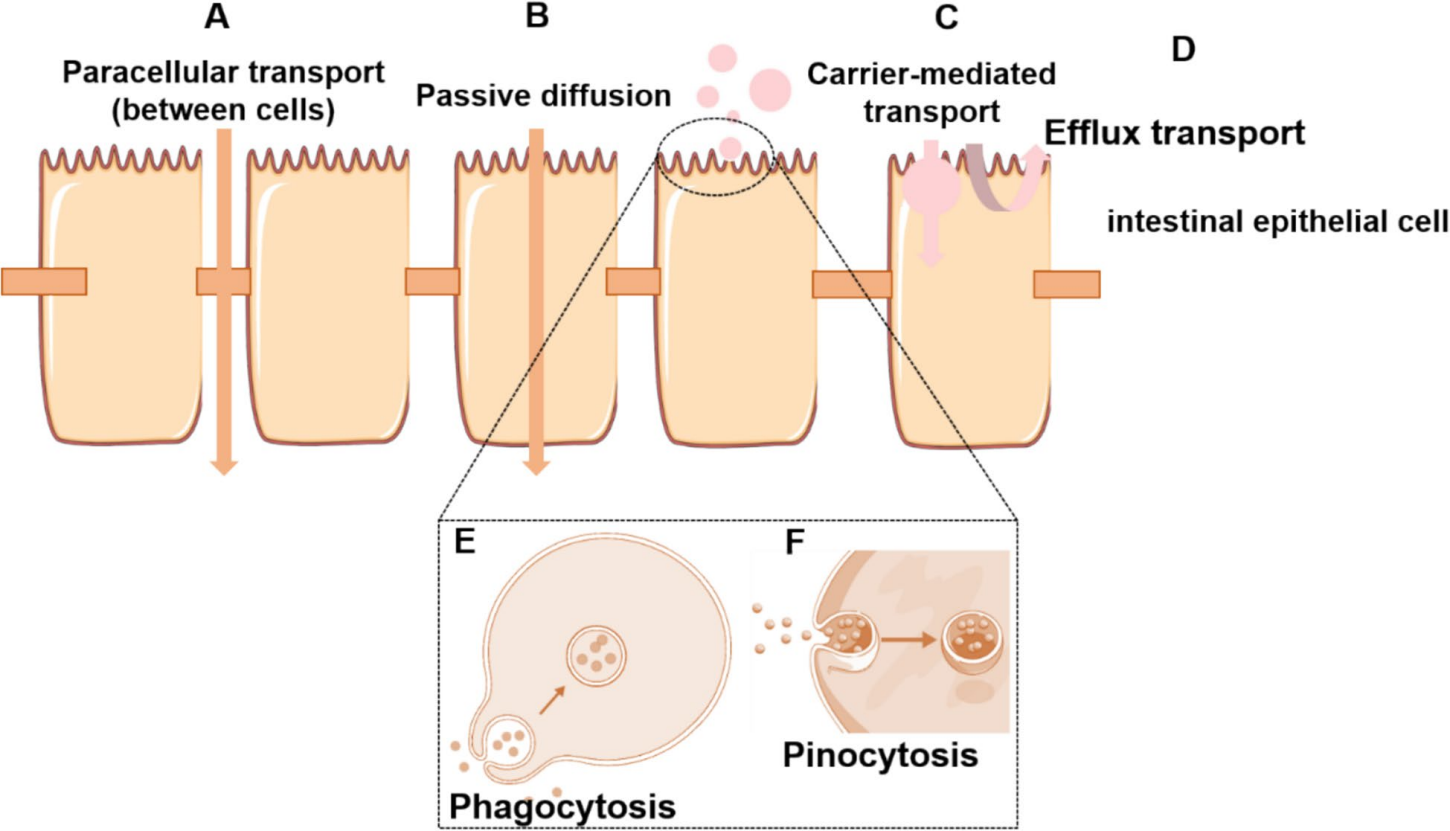
Intestinal absorption mechanisms of multi-component TCM. Schematic representation of the major transmembrane pathways for diverse TCM constituents: **A** paracellular transport-small hydrophilic molecules; **B** passive diffusion-lipophilic compounds; **C** carrier-mediated transport-molecules recognized by specific transporters; **D** efflux transport-active efflux of transporter substrates, limiting systemic absorption; **E** phagocytosis-polysaccharide aggregates and plant-derived nanoparticles; **F** pinocytosis-water-soluble macromolecules

The small intestinal mucosa provides the surface area for drug absorption and allows prolonged retention of compounds [22]. The intestinal epithelial membrane serves not only as the primary barrier to drug permeation but also as a critical determinant of gastrointestinal bioavailability. Drug transmembrane transport occurs mainly through six mechanisms: passive diffusion, paracellular transport, phagocytosis, pinocytosis, carrier-mediated transport, and efflux transport (Fig. 1). TCM constituents with different physicochemical properties follow distinct pathways: liposoluble terpenoids mainly diffuse passively, small molecule glycosides often pass through the paracellular route, while aglycones and similar compounds are recognized by specific transporters. A well-characterized example is P-glycoprotein (P-gp), an efflux transporter that actively exports substrates such as aconitine (AC) from intestinal cells. Despite its high toxicity [23], AC undergoes P-gp mediated efflux into the ileal lumen, resulting in low oral bioavailability (approximately 5–10%). Certain TCM formulations, however, can enhance AC absorption by inhibiting P-gp. For instance, The *Glycyrrhizae Radix et Rhizoma* and *Aconiti Lateralis Radix Praeparata* pair [23], Sini decoction [24], and processed *Aconiti Lateralis Radix Praeparata* combined with *Glycyrrhizae Radix* increase systemic AC exposure through dual mechanisms: P-gp efflux inhibition and glycyrrhizic acid mediated absorption enhancement. These PD synergies highlight the essential role of herb compatibility and processing in optimizing TCM bioavailability.

Distribution refers to the systemic translocation of drugs from the bloodstream to peripheral tissues. After entering circulation, TCM molecules may reversibly bind to plasma proteins, altering the concentration of free drug molecules. This is quantified by the drug-plasma protein binding (PPB), a critical parameter influencing pharmacokinetics (PK) and bioavailability. Drugs with high PPB can maintain effective plasma concentrations and prolong duration of action. However, high PPB also requires higher doses to reach therapeutic levels and may limit free drug distribution and tissue penetration, reduce bioavailability [25]. Barriers such as the blood–brain barrier (BBB) and placental barrier can further restrict drug access to target sites, reducing bioavailability [26]. Many central nervous system (CNS)-targeted drugs efficacy due to limited BBB permeability. In Traditional Chinese Medicine, aromatic resuscitating herbs are traditionally believed to “awaken the mind, open the orifices, promote circulation, and guide other medicines upward.” Modern studies show that aromatic compounds can enhance BBB permeability and lipophilicity, facilitating CNS delivery. For instance, β-asarone from *Acori Tatarinowii Rhizoma* has been formulated into chitosan nanoparticles to deliver astragaloside IV, improving nose-to-brain targeting and overcoming poor permeability and low lipophilicity [27]. The placental barrier, a unique maternal fetal interface, regulates nutrient supply and protects the fetus from harmful substances, significantly influencing ADME in the maternal fetal system [28]. Some exogenous compounds cross the placenta via passive diffusion or active transport [29]. For example, codeine and its metabolite morphine from *Papaveris Pericarpium* can penetrate the placental barrier. Codeine transport is independent of the organic cation transporter (OCT) pathway, while morphine transport is OCT-mediated [30]. Certain TCM constituents primarily cross the placenta as prototype compounds. For instance, five constituents of *Scutellariae Radix* (baicalin, wogonoside, baicalein, wogonin, and oroxylin A) have been detected in the fetus in unchanged forms [31]. Notably, PK studies on the transplacental transfer of TCM remain limited. Most reports only confirm whether active components can cross the placenta, while the mechanisms underlying their transfer have not been thoroughly investigated.

Metabolism and excretion together constitute the elimination phase of the ADME process. This stage plays a critical role in determining the systemic exposure of TCM components, the duration of therapeutic effects, and the risk of toxicity. Compounds are primarily metabolized through Phase I (oxidation, reduction, hydrolysis) and Phase II (conjugation) reactions, mainly mediated by cytochrome P450 (CYP450) enzymes and conjugating enzymes [32]. Some metabolites retain or alter the pharmacological or toxic properties of the parent compounds, thereby affecting overall exposure. For example, Baicalin is highly hydrophilic and has limited oral absorption. It requires hydrolysis by intestinal microbial β-glucuronidase to be converted into the more lipophilic baicalein, which can then be efficiently absorbed into the bloodstream. Compared to baicalin, baicalein exhibits significantly higher bioavailability and better BBB permeability, with notable distribution in brain regions such as the striatum, thalamus, and hippocampus. In contrast, baicalin relies more on enterohepatic circulation to maintain systemic exposure. Animal studies have shown that under cerebral ischemia, baicalin displays a prolonged half-life and increased brain concentration, suggesting that pathological conditions may amplify its metabolic transformation and circulation-dependent brain exposure. Further ADME studies reveal that baicalein is primarily eliminated through phase II conjugation metabolism, including glucuronidation and sulfation. After oral administration, intact baicalein is barely detectable in plasma; instead, its glucuronide and sulfate conjugates predominate. Following intravenous injection, approximately 76% of baicalein is rapidly converted into conjugated forms. Meanwhile, after oral administration of baicalin, both intact baicalin and baicalein-derived conjugates can be detected in serum. In rat models, baicalin undergoes extensive conjugation metabolism in both the ileum and jejunum. At high doses, metabolic saturation may occur, indicating a significant first-pass effect [33]. Excretion occurs primarily through the kidneys and bile, with efflux transporters such as P-gp playing a key regulatory role. Unlike single-component drugs, the multicomponent nature of TCM results in complex excretion behaviors. For instance, ginsenosides (e.g., Rg1 and Rb1) are extensively metabolized by intestinal flora, then rapidly absorbed and distributed in tissues. They exhibit distinctive hepatoenteric circulation and plasma concentration–time profiles. These compounds are mainly excreted via bile, with only 0.2% to 1.2% eliminated in urine [34]. Pathological conditions can further influence excretion. In a nephrotic syndrome rat model, the renal clearance and biliary excretion of tripterygium glycosides were significantly reduced. This led to their accumulation in plasma, liver, and kidney tissues, enhancing therapeutic effects but also increasing hepatotoxicity. Simultaneously, intestinal damage promoted fecal excretion of some components [35].

While the general ADME processes apply to most TCMs, a subset—particularly those containing inherently toxic components—presents distinct PK and safety challenges. These toxic TCMs often contain compounds with both therapeutic and toxic effects, making their systemic exposure profiles critical for clinical evaluation. Therefore, it is important to examine their ADME characteristics in greater detail, with a focus on metabolic activation, tissue accumulation, and detoxification mechanisms.

#### Toxic TCMs: risk exposure and detoxification mechanisms

Toxic TCMs represent a distinct category in PK research, as their active constituents often have a narrow therapeutic index. Their ADME characteristics—particularly absorption efficiency, metabolic activation, and excretion—directly influence clinical risk. Therefore, PK studies on toxic TCMs should account for both efficacy and toxicity. To support this, we compiled representative PK data for toxic TCMs listed in the Chinese Pharmacopoeia (ChP), as summarized in Table S1, Additional File 1. These parameters offer valuable insights into their ADME behaviors and risk profiles.

**Fig. 2:**
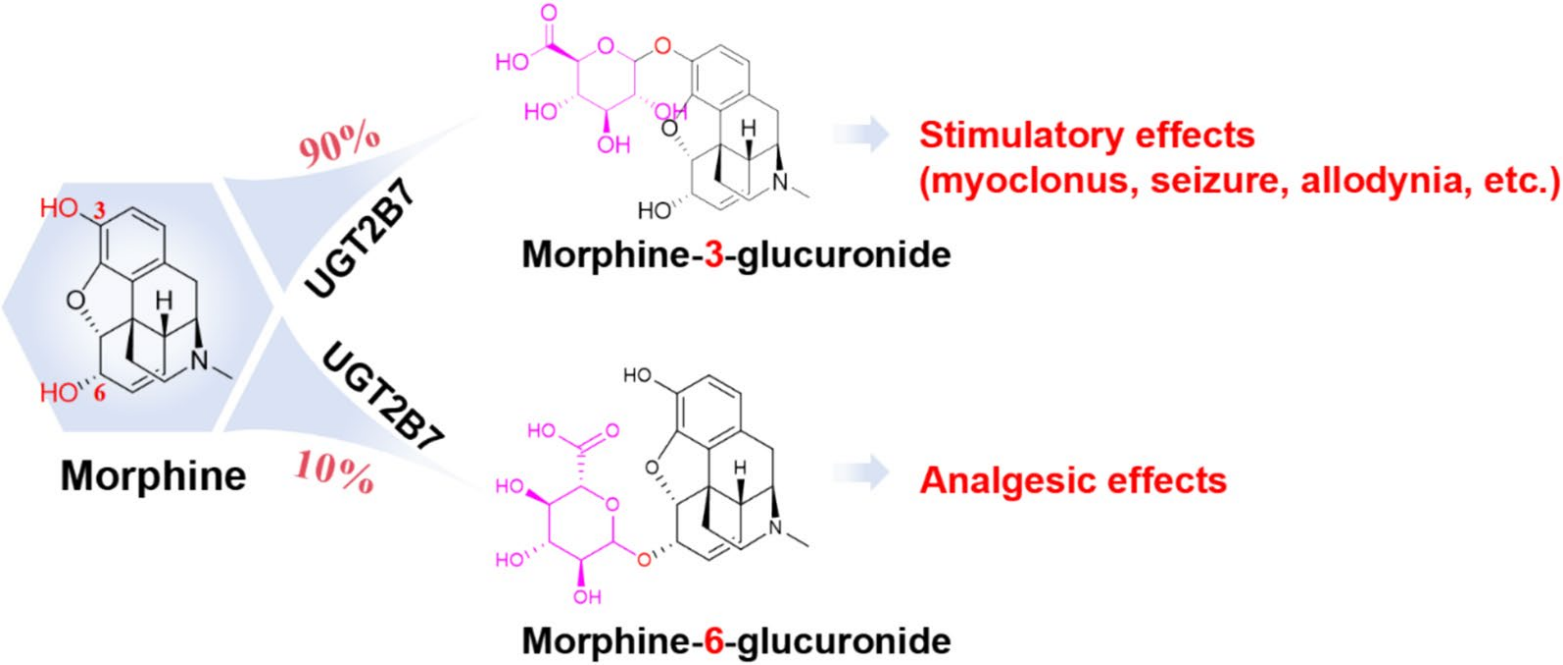
Glucuronidation of morphine(come from *Papaveris Pericarpium*) by UDP-glucuronosyltransferase 2B7

The toxic effects of certain TCMs are closely related to the systemic exposure levels of their components during metabolism. A representative example is morphine(come from *Papaveris Pericarpium*) [36], which undergoes biotransformation via UDP-glucuronosyltransferase 2B7 (UGT2B7) to form pharmacologically active glucuronides—primarily morphine-3-glucuronide and morphine-6-glucuronide (Fig. 2). PK studies in end-stage renal disease patients demonstrate distinct compartmental behaviors [37]: morphine follows a two-compartment model after oral administration, while its glucuronide metabolites exhibit one-compartment kinetics. Critically, impaired renal excretion leads to significant systemic accumulation of these metabolites, increasing the risk of toxicity. Similarly, TCMs containing heavy metals such as mercury and arsenic pose a risk of accumulation, especially when co-administered with other TCMs that alter their absorption and excretion. For instance, Baiziyangxin pills (BZYXP), which contain cinnabar (mercury sulfide), significantly affect the PK of mercury. Compared with cinnabar alone, BZYXP administration increases the maximum plasma concentration (C_max_), shortens the time to peak (T_max_), prolongs the half-life (T_1/2_), reduces clearance (CL), and increases the area under the concentration–time curve (AUC), indicating a higher risk of systemic accumulation. Tissue distribution studies reveal that mercury primarily accumulates in the liver, kidneys, and brain. After 30 days of administration, mercury levels in the brain were significantly higher in the BZYXP group than in the cinnabar-only group. Although no overt liver or kidney damage was observed even at tenfold doses, pathological analysis showed cerebral vascular edema and reduced spontaneous activity in rats, suggesting neurotoxicity. Mechanistically, certain components in BZYXP (such as *Glycyrrhizae Radix et Rhizoma* and *Angelicae Sinensis Radix*) may enhance mercury solubility and absorption by modifying the gastrointestinal environment, thereby prolonging mercury retention in the body. These findings indicate that even at therapeutic doses, long-term use of BZYXP may lead to mercury accumulation in the brain and potential neurotoxicity. Clinical use should involve strict control of dosage and duration, and re-evaluation of safety standards for cinnabar-containing TCMs is warranted [38]. In contrast, based on the TCM compatibility principle, some courier(shi) TCM, such as *Glycyrrhizae Radix et Rhizoma* have been shown to reduce toxicity. Modern studies indicate that licorice can inhibit the intestinal absorption of diester diterpene alkaloids from Aconitum species and promote their metabolism and excretion through the induction or regulation of drug-metabolizing enzymes and efflux transporters, thereby mitigating toxicity [39].

The ADME process determines the systemic exposure, therapeutic efficacy, and safety profile of TCM components, serving as a core mechanism in regulating bioavailability. Given the diverse ADME characteristics exhibited by many TCMs, comprehensive evaluation and monitoring of their absorption, distribution, metabolism, and excretion are essential. Evaluating systemic exposure is crucial for toxic TCMs, given the frequent coexistence of therapeutic effects and toxicities. However, fewer than 100 toxic herbs are recorded in the ChP, and their PK studies remain limited, outdated, and insufficient, highlighting the need for further investigation into their efficacy and safety.

### Analysis of TCM under ADME mechanism

Although TCM ADME studies often focus on representative compounds due to technical limitations, the ultimate goal is to understand the overall PK behavior of all bioactive constituents. The examples presented in this review illustrate common methodologies and principles, aiming to reflect systemic patterns relevant to the whole TCM.

#### Analysis of active substances

Investigating the ADME characteristics of TCM bioactive components requires advanced, high-throughput analytical platforms, especially for efficient sample processing and metabolite profiling [40]. Current protocols typically involve systematic collection of biospecimens (such as plasma, serum, urine, bile, and tissue homogenates) for pharmacokinetic trajectory mapping. Automated LC–MS/MS systems are widely used for simultaneous multi-component quantification. While traditional methods like protein precipitation, liquid–liquid extraction, and solid-phase extraction (SPE) remain common, newer techniques (such as microextraction, microdialysis, and automated sample preparation) are gaining popularity due to their minimal sample loss, reduced matrix interference, and improved efficiency [40, 41]. For example, Fu et al. [42] employed microdialysis coupled with high-performance liquid chromatography-triple quadrupole mass spectrometry to compare the PK of 10 lignans in plasma and cerebrospinal fluid (CSF) between healthy and Alzheimer's disease model rats. The study showed that disease status significantly altered systemic exposure profiles in both plasma and CSF. These findings highlight how pathological conditions can modify the integrated ADME behavior of TCM components, potentially influencing their therapeutic efficacy.

At present, Liquid chromatography-mass spectrometry (LC–MS) and gas chromatography-mass spectrometry (GC–MS) remain the gold standards for qualitative and quantitative profiling. GC–MS is particularly suitable for volatile compounds (e.g., monoterpenes, sesquiterpenes, aromatic compounds in volatile oils) and thermally stable components that can be derivatized into volatile forms (e.g., through silanization, acylation, esterification). These include certain organic acids, sugars, sterols, and fatty acids. Researchers often use GC–MS to profile volatile constituents in *Artemisiae Argyi Folium* [43], *Pogostemonis Herba* [44], and *Cinnamomi Cortex* [45], and their formulations to track absorption and metabolism. However, GC–MS is less effective for strongly polar, thermally labile, or high-molecular-weight compounds. In such cases, LC–MS offers advantages. For example, Tong et al. [46] used ultra performance liquid chromatography-quadrupole time of flight-mass spectrometry to analyze the chemical components, metabolites, and PK of Zhijuntangshen decoction. They identified multiple constituents with favorable absorption and distribution, supporting its clinical use from a systemic exposure perspective. Building on such multi-component PK studies, recent research has integrated metabolomics and network pharmacology to explore synergistic mechanisms in TCM formulas. Du et al. [47] combined PK, pharmacometabolomics, and dynamic network analysis to investigate Baoyuan decoction (BYD) for cardiac hypertrophy. They constructed a network linking major bioactive groups (e.g., saponins, flavonoids) with disturbed metabolic pathways (e.g., gut microbiota metabolism, amino acid metabolism, energy homeostasis), revealing how BYD modulates key pathological processes. In another study, Wang et al. [48] employed matrix-assisted laser desorption/ionization mass spectrometry imaging to visualize the spatial distribution of baicalin and its metabolites in mouse kidney tissue. Their findings showed distinct localization patterns: baicalin and wogonin were enriched in both the renal cortex and medulla, while other metabolites were mainly confined to the cortex. This spatial profiling highlights the tissue-specific metabolic fate of TCM components and underscores the importance of compartmentalized exposure in evaluating efficacy and safety.

While most analytical strategies for TCM target low-molecular-weight compounds such as flavonoids, alkaloids, and terpenes, polysaccharides represent another important class of bioactive substances with distinct challenges. Due to their high molecular weight, structural heterogeneity, and lack of chromophores or fluorophores [49], specialized analytical frameworks are required to characterize their in vivo behavior. Current approaches often use fluorescent or radiolabeling techniques to trace polysaccharides in vivo [50]. For instance, *Lycii Fructus* polysaccharides (LBP) are complex high-molecular-weight mixtures, and tracking their PK is particularly difficult. Using FITC-labeled LBP combined with high-performance gel permeation chromatography and fluorescence detection, researchers monitored the systemic fate of the polysaccharide fraction. The results showed that most of the administered LBP was excreted in feces, with minimal systemic absorption (T_max_: 2.00–2.33 h; T_1/2_: 31.39–45.76 h; MRT: 18.38–20.07 h) [51]. This study highlights an effective strategy for investigating the holistic PK of complex macromolecular components and provides valuable data on the bioavailability of polysaccharides in TCM.

GC–MS and LC–MS datasets of pharmaceutically active compounds often contain numerous known and unknown metabolites. AI offers significant advantages in handling such complex data, particularly in metabolite identification, predictive modeling, and high-throughput analysis. Compared to conventional methods, AI demonstrates superior accuracy and computational efficiency [52]. Kensert et al. [53] developed a YOLO-based one-dimensional convolutional neural network (1D CNN) model for direct chromatogram analysis. This model enables simultaneous peak detection, confidence estimation, and area quantification from raw chromatographic profiles. It achieved excellent performance (ROC-AUC: 0.996), outperforming traditional methods such as Savitzky-Golay smoothing by 8.6% in true positive rate on real chromatographic data. Although the model was trained solely on simulated chromatograms (1 million profiles generated under varied conditions), it represents an innovative advancement in AI-driven chromatographic analysis. Previously, AI has been successfully applied in TCM for medicinal material identification and formulation process optimization, valued for its automation, high throughput, and precision. However, PK and bioavailability analyses of TCM components still rely heavily on manual processing, with limited AI integration. Moreover, component datasets in TCM lack standardized labeling and unified data formats. Future efforts should focus on developing standardized, high-quality datasets and exploring AI applications in PK analysis, aiming to enhance the efficiency, accuracy, and reproducibility of TCM PK studies.

#### Analysis of endogenous substances and metabolites

Endogenous substances in TCM include not only therapeutic constituents but also inherent toxic compounds, such as AC, pyrrolizidine alkaloids (PAs), aristolochic acid, and anthraquinones [54]. These substances often exhibit dual roles, both pharmacologically active and potentially toxic. Therefore, evaluating the effectiveness of TCM must be integrated with safety assessment, taking into account the complete ADME process rather than focusing solely on original components. The ADME behavior of endogenous toxic substances and their in vivo metabolites has become a key area of research. Modern analytical technologies have significantly enhanced our ability to elucidate their mechanisms and define safety windows. Traditional methods such as processing and compatibility have long been used to mitigate toxicity, and their scientific basis is now being explored and validated through advanced platforms. Importantly, core analytical platforms used for studying active components are also applicable to toxic substances. However, due to their unique characteristics (e.g., trace levels, metabolic activation, and high toxicity), these compounds require specialized analytical strategies with heightened demands for sensitivity, selectivity, and accurate metabolite identification [55].

Due to the presence of numerous endogenous interfering substances in biological samples, efficient separation and enrichment of trace toxic components and their metabolites are essential. SPE remains the mainstream method, with specific adsorbents often selected to target particular toxin categories [56, 57]. In recent years, the Quick, Easy, Cheap, Effective, Rugged, Safe (QuEChERS) method has been adapted for extracting endogenous toxins such as PAs from plant and biological matrices. It integrates extraction and dispersive purification steps, offering high efficiency in removing complex interferences [58, 59]. Immunoaffinity chromatography provides excellent selectivity by immobilizing toxin-specific antibodies on the column to capture target toxins or their metabolites. This approach significantly enhances sensitivity and is particularly suitable for ultra-trace toxicity marker analysis [60]. However, its application remains largely limited to pesticide detection in food and environmental samples. In the context of Traditional Chinese Medicine, its use is still rare. Existing studies mainly focus on the enrichment of compounds like hesperidin and naringin from preparations containing dried tangerine peel [61].

At present, LC–MS is the primary method for analyzing endogenous toxic substances and their metabolites. For instance, *Ginseng Radix et Rhizoma* and *Aconiti Lateralis Radix Praeparata* are often used in combination. Xu et al. [62] found that ginsenoside Rg1 (the main active compound in *Ginseng Radix et Rhizoma*) enhanced the metabolism of AC, promoting its rapid conversion into less toxic monoester alkaloids. This effect was attributed to Rg1-induced activation of CYP450 enzymes, which accelerate the biotransformation of toxic diester diterpene alkaloids into their monoester forms. Interestingly, these monoester alkaloids also demonstrate anti-inflammatory activity, suggesting a dual benefit of reduced toxicity and enhanced efficacy. This study provides a methodological reference for exploring the metabolic mechanisms and personalized risks associated with herb pairs. It also offers mechanistic insight into the traditional concept of detoxification through TCM compatibility.

Among the variants of GC–MS, headspace gas chromatography–mass spectrometry is notable for its ability to directly detect volatile toxic metabolites in complex biological matrices such as blood, urine, and tissues. This technique enables in vivo monitoring with minimal sample preparation [63, 64]. Although direct studies on volatile toxic metabolites in TCM and formulas remain limited, it has been successfully applied to assess changes in volatile components during processing and to detect contamination in TCM [65, 66]. In parallel, high-resolution mass spectrometry (HRMS) offers powerful non-targeted scanning capabilities. It can systematically capture unknown metabolites produced from the biotransformation of toxic components in TCM. By combining HRMS with computational toxicology tools (e.g., Discovery Studio), researchers can perform preliminary toxicity risk assessments and predict potential toxic mechanisms through metabolic activation pathway analysis. These approaches help guide subsequent experimental validation.

PAs are not directly toxic themselves. However, after hepatic metabolism by CYP450 enzymes, they are converted into reactive pyrrole metabolites (e.g., dehydropyrrole), which can form covalent adducts with DNA, RNA, and proteins. This leads to cytotoxicity, genotoxicity, and ultimately liver injur [67]. As such, PAs serve as a representative model for studying the ADME behavior of complex toxic compounds. Their analysis integrates multiple advanced technologies [68]. Sample pretreatment methods such as SPE and QuEChERS have enabled sensitive and specific detection of trace PAs and their key hepatotoxic metabolites in biological samples, supporting in vivo exposure profiling. In parallel, HRMS was used for non-targeted screening, allowing comprehensive identification of unknown metabolites, including secondary oxidation or conjugation products [69]. For these newly discovered metabolites, researchers applied fragment profiling, pathway prediction, and computational toxicology tools to preliminarily assess hepatotoxic risk. Subsequently, metabolite exposure patterns were correlated with in vitro hepatocyte toxicity assays. This not only validated the predictive models but also clarified the molecular mechanism of PAs-induced liver injury, characterized by “metabolic activation to covalent binding to hepatic damage” [70]. This integrated methodological framework demonstrates how modern analytical and computational approaches can trace the fate of highly toxic substances, from exposure and metabolism to risk assessment and mechanistic elucidation. It provides a valuable paradigm for studying similar toxicants in TCM [71].

Modern analytical techniques provide a powerful toolkit for investigating the ADME behavior of endogenous toxic substances in TCM. These technologies enable precise mapping of in vivo exposure trajectories, identification of key active or toxic metabolites, and clarification of metabolic activation or detoxification mechanisms. Such insights are essential for understanding the toxicological basis of TCM, evaluating its safety window, and guiding safe clinical use(such as optimizing processing methods and informing compatibility practices). Ultimately, these techniques play a central role in quality control of TCM.

#### AI-driven prediction of ADME and toxicity properties

Given the critical role of ADME processes in drug efficacy and safety, various AI-driven models have been developed to predict PK behaviors. Considering the unique ADME characteristics of toxic substances, recent studies have integrated toxicity prediction with ADME modeling, collectively referred to as ADMET prediction. These tools are essential for early screening of compounds with favorable bioavailability, reduced toxicity risk, and therapeutic potential, offering valuable support for evaluating the effectiveness and safety of complex constituents in TCM.

**Fig. 3:**
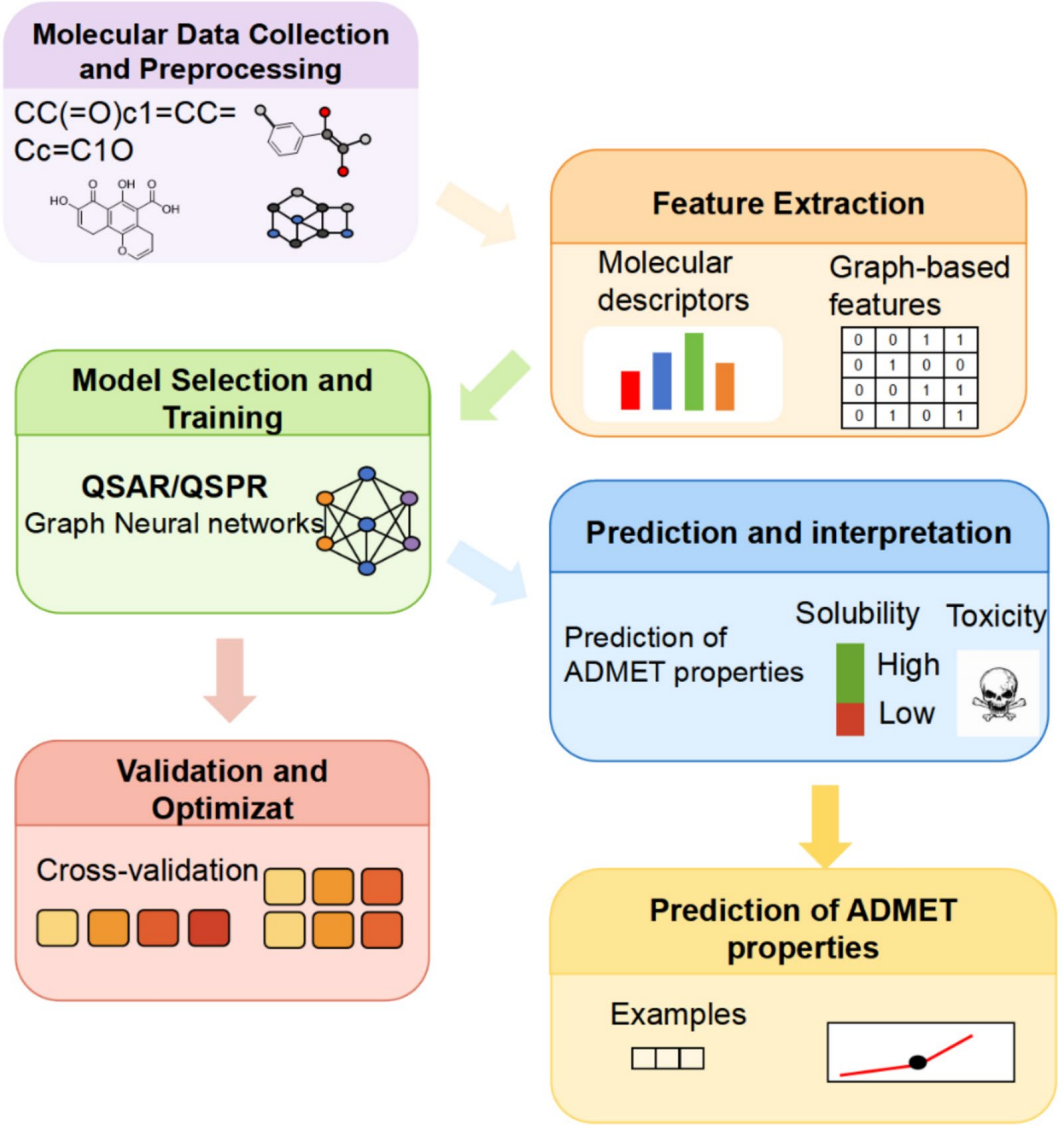
The workflow of predicting the properties of the molecule ADME and toxicity using AI

With advances in computer science, the growing availability of ADMET data has driven the development of computational models—such as k-nearest neighbors and random forest (RF)—for predicting ADMET properties and compound bioavailability. These models significantly reduce the cost and time required for early screening in TCM research [72, 73]. Currently, Quantitative Structure–Activity Relationship (QSAR) and Quantitative Structure–Property Relationship (QSPR) modeling are the most widely used approaches. By calculating molecular descriptors, these models establish correlations between chemical structures and their PK properties (including ADME) or toxicity endpoints [74]. Figure 3 illustrates a typical AI-powered ADMET prediction workflow based on QSAR/QSPR principles. Tools such as ADMETlab 3.0 [75] and SwissADME [76] use QSPR models to predict properties like solubility, membrane permeability, metabolic stability, and potential toxicity from molecular structures and SMILES strings. Table 1 summarizes several publicly available ADMET prediction platforms.

**Table 1:** Chemical ADMET properties prediction tools

Underlying principle	Name	Function	Refs.
QSAR	SwissADME	Submission of molecular structures, SMILES, or InChI enables ADMET property prediction	[76]
	TCMSP	The database uses tools created by the developers themselves to predict and upload ADMET features	[77]
	XenoSite	Submission of molecular structures or SMILES enables prediction of CYP450 metabolism, metabolic sites, and metabolite structures	[78]
	Pred-Skin	Submission of molecular structures or SMILES codes to predict the degree of absorption of drug molecules in the skin and to assess their potential toxicity and safety	[79]
	CypRules	Prediction of CYP450 metabolic inhibition using Mold2 2D descriptors	[80]
	pKCSM	Submit molecular structure or SMILES code, predicting ADMET properties based on graph signatures	[81]
	Interpretable-ADMET	It predicts specific ADMET properties, identifies substructural contributions, and enables automatic lead optimization. Molecular SMILES support precise, property-range, and structure-similarity searches	[82]
	vNN-ADMET	VNN-based modeling to predict compound ADMET properties	[83]
	QikProp	ADME prediction tool for compounds from Schrödinger	[84]
Extremely Randomized Trees	Hit Dexter	Submission of molecular structures or SMILES codes to predict compounds ADMET properties	[85]
	NERDD	Prediction of metabolic sites and metabolites of organic small molecules, labeling of compounds that may interfere with bioassays, identification of natural products and their derivatives in large libraries of compounds	[86]
DMPNN	ADMETlab 3.0	Predicting compound ADMET properties using molecular structure, SMILES code or InChI identifier	[75]
Rule-Based System	BioTransformer 3.0	Submission of molecular structures or SMILES codes to predict mammalian small molecule metabolism, gut microbiota, and soil/aquatic microbiota	[87]
Graph Neural Network	admetSAR3.0	Submission of molecular structures or SMILES codes to predict compounds ADMET properties	[88]
XLOGP3	FAFDrugs4	Submission of molecular structures or SMILES codes to predict compounds ADMET properties	[89]

In recent years, DL as a subset of AI, has gained momentum. Algorithms such as CNN and artificial neural networks (ANN) have been increasingly applied to enhance prediction accuracy. Deep QSAR modeling is emerging as an advanced approach within the QSAR domain [90]. Although its application in TCM is still at an early stage, DL offers a systematic framework that holds great potential for predicting ADMET properties in TCM.

### Cross-cutting factors modulating bioavailability

Although the ADME mechanisms and analytical methods related to TCM bioavailability have been discussed earlier, the actual bioavailability of TCM is also dynamically shaped by biological regulatory factors beyond inherent chemical properties. Two interrelated systems play critical roles: (1) the gut microbiota, which interacts with TCM compounds through metabolic transformation, probiotic enrichment, and microbiome modulation, thereby influencing both pharmacological activity and bioavailability [91]; (2) multi-component interactions at the pharmacokinetic level, particularly herb-herb interactions (HHI) and herb-drug interactions (HDI), which mediate synergistic or antagonistic effects and are increasingly recognized as key modulators of TCM bioavailability through complex and multi-mechanistic pathway [92].

These key intersecting factors reflect the holistic concept of Traditional Chinese Medicine. They highlight that the bioavailability of TCM arises from a dynamic network involving the host, microbiota, and compounds, rather than from isolated constituents alone. This perspective helps to better understand the variability in TCM bioavailability and its overall therapeutic effects.

#### Gut microbiota-mediated metabolism

The gut microbiota modulates the pharmacological activity and bioavailability of TCM constituents through various metabolic pathways, including glycosidic hydrolysis, carbonyl reduction, and deglucuronidation. In turn, TCM constituents can influence the composition and function of the gut microbiota by promoting beneficial bacteria, restoring microbial balance, and regulating microbial metabolites.

Quercetin and its glycoside, quercetin-3-rhamnoside, are widely present in TCMs such as *Mori Folium* and *Bupleuri Radix*. Due to its chemical structure, quercitrin has low oral bioavailability. However, gut microbes such as *Bacillus subtilis* and *Clostridium* K-60 can convert quercitrin into quercetin via dioxygenase or α-L-rhamnosidase. This transformation enhances the pharmacological effects of quercetin, including antihyperglycemic, antihyperlipidemic, antihypertensive, and anti-obesity activities [93]. Berberine, another representative TCM constituent for metabolic disorders, also exerts its effects through bidirectional interaction with the gut microbiota. On one hand, oral berberine is metabolized by nitroreductases into dihydroberberine, a more absorbable form with approximately fivefold higher intestinal uptake. Dihydroberberine is then reoxidized back to berberine in intestinal tissues, resulting in increased systemic exposure and improved bioavailability. On the other hand, berberine itself reshapes gut microbial composition, restores dysbiosis, strengthens the intestinal barrier, and reduces metabolic inflammation and endotoxemia [94].

Anthocyanins are another group of compounds with very low oral bioavailability, less than 2% is typically absorbed, they are highly sensitive to temperature, pH, and light [95]. Upon ingestion, anthocyanins are partially absorbed in the stomach, while the remainder reaches the intestine, where glycosidic bonds are hydrolyzed by lactase-phlorizin hydrolase. Only a small portion is absorbed by intestinal epithelial cells; most are extensively metabolized in the colon by microbial enzymes (e.g., β-D-glucosidases, β-D-glucuronidases) into simple phenolic acids such as gallic acid and protocatechuic acid, which are more readily absorbed [96, 97]. Nano drug delivery systems (NDDS) have been developed to enhance anthocyanin absorption by improving their stability and permeability [98]. Moreover, studies have shown that anthocyanins can modulate the gut microbiota by increasing beneficial bacteria through the production of short-chain fatty acids [99].

Current studies have shown that the gut microbiota is a key regulator of the PK and PD of TCM. The bidirectional interaction between TCM and the gut microbiota aligns with the holistic concept of Traditional Chinese Medicine, in addition to mediating these interactions, microbial metabolites further contribute by repairing the intestinal barrier, reducing inflammation, and enhancing the efficacy of TCM in treating metabolic and chronic diseases. Although this field holds great promise, it is still limited by factors such as individual variability, discrepancies between in vivo and in vitro findings, and the lack of standardized evaluation systems. Future progress will rely on multi-omics integration and the development of microbiota-responsive drug delivery systems to advance precision TCM therapy.

#### Herb-herb interactions and herb-drug interactions

The clinical efficacy of TCM relies heavily on multi-component formulas guided by compatibility principles. Their synergistic effects are largely mediated by complex PK interactions, particularly HHI and HDI. These interactions dynamically reshape the ADME processes of co-administered components, directly affecting their in vivo exposure, bioavailability, therapeutic efficacy, and safety. Understanding the roles of key biological barriers (such as metabolic enzymes, transporters, intestinal microbiota, and plasma proteins) is essential for addressing three core challenges: (1) explaining clinical variability in efficacy and safety; (2) providing mechanistic support for compatibility principles in Traditional Chinese Medicine and integrated Traditional Chinese-Western medicine therapy; (3) revealing multi-target regulatory networks during ADME. These insights contribute to the development of precision medication and quality control models characterized by “predictable efficacy and controllable risks” [92].

Metabolic enzymes are central to drug elimination. CYP450 enzymes catalyze the metabolism of nearly 90% of clinical drugs [100, 101]. For instance, the poor oral bioavailability of osthole is due to rapid metabolism by CYP3A4. In the Bushenyizhi formula, Schisandrin B (a known CYP3A4 inhibitor) significantly enhances the bioavailability of osthole by reducing its metabolism, supporting the compatibility rationale of the formula [102]. Metabolic enzymes and transporters often co-regulate drug elimination, sharing substrates and tissue distribution. For example, in the Shengmai yin, *Schisandrae Chinensis Fructus* (assistant TCM) enhances the absorption of ginsenosides from *Ginseng Radix et Rhizoma* (monarch TCM) by inhibiting both P-gp and CYP3A4. This dual inhibition improves ginsenoside oral bioavailability, similar to the action of ketoconazole [103]. In contrast, HDI can also lead to negative outcomes. Co-administration of Yougui yin and omeprazole significantly increased CYP2C19 expression, resulting in reduced plasma exposure of omeprazole (AUC_0-∞_ reduce 50.3%, AUC_0-T_ reduce 47.6%, C_max_ reduce 43.1%) and potentially compromising its therapeutic effect [104].

Transporters like PEPT1 and PEPT2 mediate the uptake of various TCM peptides. For example, Ganoderma lucidum triterpenoids enhance the intestinal absorption of cyclic peptides from *Notoginseng Radix* by upregulating PEPT1 expression, explaining the synergistic effect of this TCM pair [105]. OCT1/2/3 and MATE1 also play critical roles. Berberine inhibits OCT1/2 and MATE1, increasing the plasma concentration and AUC of metformin while reducing its clearance. Metformin, in turn, enhances berberine retention in tissues. This mutual inhibition of OCT/MATE1 transport generates a beneficial HDI [106]. Not all interactions are positive. *Rhizoma Dioscoreae Bulbiferae* extract, though anti-tumor, downregulates P-gp and MRP2, reducing pirarubicin efflux and increasing its accumulation and cardiotoxicity. This suggests that their combination may be unsafe [107].

Intestinal microbiota also serve as a metabolic interface in HHI and HDI. Bacterial enzymes such as β-glucuronidase, abundant in *Bifidobacterium* and *Lactobacillus*, promote the biotransformation of TCM compounds into active metabolites [108]. Tyrosine decarboxylase (TDC) converts L-tyrosine and levodopa into tyramine and dopamine, respectively, reducing levodopa’s brain availability. Co-administration with piperine reduces TDC levels, increases AUC_0–10_ of levodopa by 17.6%, and enhances its striatal concentration by 2.43-fold, showing marked synergy [109, 110].

Plasma protein binding also affects drug distribution. Tanshinone IIA and berberine can displace warfarin from human serum albumin (HSA), increasing free warfarin levels and enhancing its anticoagulant effect. PK studies show that co-administration prolongs coagulation time in mice [111].

Ultimately, HHI and HDI represent dynamic interactions between TCM components and physiological barriers. Assistant and courier TCMs often enhance bioavailability by inhibiting enzymes or transporters, embodying the Traditional Chinese Medicine principle of “enhancing efficacy and reducing toxicity”. HDI research also provides critical evidence for safe clinical co-administration. However, due to the complex composition of TCM and inter-individual variability, the actual PK behavior of many compounds remains poorly understood. More clinical studies and mechanistic investigations are needed to fully elucidate these interactions and guide rational use.

## Improving the bioavailability of TCM

Implementing bioavailability-enhancing strategies in TCM must align with the holistic concept. This requires not only optimizing the bioavailability of key active components but also maintaining the integrity and balance of complex TCM matrices. Risk control from both endogenous and exogenous sources is essential to achieve predictable clinical efficacy and controllable quality risks.

### Optimizing the quality of raw Chinese medicines

Medicinal plants are the foundation of TCM, and their quality directly affects the safety, efficacy, and bioavailability of herbal formulations. Environmental factors during cultivation influence the biosynthesis of active compounds. Exogenous contaminants (such as heavy metals, pesticide residues, mycotoxins, and sulfur dioxide (SO_2_)) pose significant safety risks. Standardized cultivation, rational agricultural input management, and regulated processing and storage are critical to ensuring raw material quality. The “Good Agricultural Practice” (GAP) guidelines systematically regulate origin selection, germplasm, planting, harvesting, processing, storage, and traceability [112].

The concept of “genuine producing area” and “geo-authentic medicinal materials”, as recorded in Shennong Bencao Jing Jizhu, reflects the synergistic role of genetics and ecological conditions in determining medicinal quality. While TCM contains diverse compounds, current cultivation efforts focus on enhancing key active constituents [113]. Multi-omics technologies are increasingly applied to explore biosynthesis regulation, geo-authenticity mechanisms, and plant breeding [114]. For example, SUN et al. [115] used transcriptomics and metabolomics to compare *Cistanches Herba* from different regions and found that saline-grown samples had the highest levels of phenylethanol glycosides, supporting their geo-authenticity. Planting management is also essential. Continuous cropping obstacles are common in TCM agriculture. Peng et al. [116] demonstrated that intercropping *Atractylodis Rhizoma* awith corn increased biomass and volatile oil content, promoting growth and quality.

Exogenous contaminants can reduce bioavailability through three main mechanisms: (1) direct toxicity: heavy metals and mycotoxins impair liver and kidney function, altering drug clearanc; (2) intestinal barrier disruption: pesticide and fertilizer residues may induce enteritis and impair absorption; (3) microbiota imbalance: residual SO_2_ suppresses probiotics, affecting microbial activation of active compounds. International and national bodies have established maximum residue limits for common pollutants. The ChP specifies detection methods, limits, and preliminary risk assessments. Bioavailability and bioaccessibility are now key indicators in pollutant risk evaluation, with the Caco-2 model being widely used for bioaccessibility testing.

Heavy metals are high-density elements (> 5.0 g/cm^3^) with known toxicity, including mercury, arsenic, lead, cadmium, etc. [117]. Sui et al. [118] applied a UBM/Caco-2 digestion model to assess heavy metal bioaccessibility in medicinal teas. Chromium was the most prevalent contaminant, with bioaccessibility ranging from 26.6% to 99.3% and bioavailability from 0 to 31.6%. To reduce the content of heavy metals in TCM, the first step is using phytoremediation and soil amendments; Secondly, selection of low-accumulation germplasm; Thirdly, in the processing, preparation and extraction stages, techniques such as water grinding, membrane separation and supercritical extraction can be applied [119]. At the same time, heavy metal content in medicinal materials should be analyzed using methods such as inductively coupled plasma mass spectrometry and atomic absorption spectrometry, with emerging technologies like sensor-based systems offering new possibilities for one-stop control [120, 121]. Table 2 summarizes advanced detection techniques for heavy metals in TCM.

**Table 2:** Detection methods for heavy metal ions in TCMs

Analytes		TCMs		Detection method	Linear range		LOD		Refs.
Hg^2+^		Aole Paidu capsule		ZGO: Mn NRs and HCD Triple Signal Sensor	2–11 μM; 8–35 μM; 57–134 μM		0.103 μM; 0.032 μM; 2.346 μM		[247]
		*Astragali Radix; Ginseng Radix et Rhizoma*		ratiometric electrochemical sensor	0.01–1.0 μM		6.42 nM		[248]
		*Laminariae Thallus*		SWASV	0.01–0.10 μg/mL		0.138 mg/kg		[249]
		*Chrysanthemi Flos; Citri Reticulatae Pericarpium*		Three-channel fluorescence array sensor	0.38–338 μmol/L		0.15 μmol/L		[250]
		*Astragali Radix*		ratiometric fluorescent detection	0–0.6 μM		0.022 μM		[251]
Pb^2+^		*Astragali Radix; Ginseng Radix et Rhizoma*		ratiometric electrochemical sensor	0.01–1.0 μM		5.47 nM		[248]
		*Astragali Radix; Glycyrrhizae Radix et Rhizoma; Panacis Quinquefolii Radix; Lonicerae Japonicae Flos*		Peptide-based nanochannel sensor	0.01–0.16 μM; 10–100 μM		0.005 μM		[252]
		*Chrysanthemi Flos; Citri Reticulatae Pericarpium*		Three-channel fluorescence array sensor	0.38–338 μmol/L		0.15 μmol/L		[250]
Cd^2+^		*Astragali Radix; Ginseng Radix et Rhizoma*		ratiometric electrochemical sensor	0.01–1.0 μM		1.08 nM		[248]
		*Licorice Alisma Salvia Poria*		DPASV	2.9 nM–4.6 mM		2.0 nM		[253]
Cd^2+^	*Chrysanthemi Flos; Citri Reticulatae Pericarpium*		Three-channel fluorescence array sensor			0.38–338 μmol/L		0.15 μmol/L	[250]
As^3+^	*Angelicae Sinensis Radix; Dioscoreae Rhizoma; Paeoniae Radix Alba; Achyranthis Bidentatae Radix*		Electrochemical sensor			2.7–40 nM		0.42 nM	[254]
Cu^2+^	*Laminariae Thallus*		SWASV			0.001–0.01 μg/mL		1.51 mg/kg	[249]
	*Phyllanthi Fructus*		ICP-OES			0.4–700 μg/L		0.16 μg/L	[255]
	*Astragali Radix*		ratiometric fluorescent detection			0–2 μM		0.057 μM	[251]
	*Schisandrae Chinensis Fructus; Chuanxiong Rhizoma; Alismatis Rhizoma*		TR-V probe for copper ion detection			1–100 μM		84 nM	[256]
Ag^+^	*Astragali Radix*		ratiometric fluorescent detection			0–1 μM		0.045 μM	[251]

Pesticide residues include organophosphates, organochlorines, and pyrethroids, which enter the body via the food chain and tend to accumulate in lipid-rich organs [122]. Such as *Lonicerae Japonicae Flos* is prone to the influence of pesticide residues [123]. To reduce pesticide residues, on the one hand, it is necessary to cooperate with the planting environment, reduce the use of pesticides, especially highly toxic chemical pesticides, and adopt alternative control technologies such as biological pesticides, insect-proof nets and insecticidal lamps. In particular, it is important to develop smart agriculture and make full use of modern technologies [124, 125]; On the other hand, processing and biodegradation strategies should be emphasized to fully implement the Traditional Chinese Medicine theory of “reducing toxicity and enhancing efficacy” [126]. Finally, rapid and high-throughput screening work should be achieved through precise detection. At present, there are already ML methods to help detect pesticide residues, half-lives and exposure levels in TCM. We will introduce them in later. Table 3 highlights detection methods for pesticide residues in TCM.

**Table 3:** Detection methods for pesticide residues in TCMs

Analytes	TCMs	Method	Pretreatment-material	Linear range	LOD	Refs.
Organophosphorus pesticide	*Ginseng Radix et Rhizoma; Salviae Miltiorrhizae Radix et Rhizoma*	Enzymatic reaction-modulated HG-AFS	Enzymatic modulation of hydrolysis	0.1–10 μg/mL	0.043 μg/mL	[257]
Imidacloprid	*Notoginseng Radix et Rhizoma; Dioscoreae Rhizome; Astragali Radix*	Ic-ELISA	Monoclonal antibodies	0.2–9.6 ng/mL	0.1 ng/mL	[258]
Methyl-paraoxon	*Ginseng Radix et Rhizoma; Nelumbinis Semen*	Fluorescence Colorimetric	fluorescent carbon based composite	1.25–20 μM; 20–60 μM; 14.3–285 μM	1.25 µM; 14.3 µM	[259]
Chlorpyrifos	*Salviae Miltiorrhizae Radix et Rhizoma; Codonopsis Radix*	Dual-readout ICA	C3N4-BiFeO3 nanocomposites	0.1–60 ng/mL	0.033 ng/mL	[260]
Acetamiprid	*Dioscoreae Rhizoma; Pseudostellariae Radix*	Fluorescent aptasensor	AuNPs	0.1–3 mg/mL	0.0285 mg/mL	[261]
Thiram Paraquat	*Coicis Semen; Ginseng Radix et Rhizoma*	Ratio Fluorescence Sensing System	N-CQDs@CuNCs Complex	10–500 nM; 5–100 nM	7.49 nM; 3.03 nM	[262]
Carbamate	*Ginseng Radix et Rhizoma; Poria Dioscorea opposita*	Fluorescence	CdSe-ZnS QDs	2–50 nM; 0–5000 nM; 50–5000 nM	2 nM; 50 nM; 50 nM	[263]
Deltamethrin	Wheat	SERS	Ag@ZnO NFs	1.0 × 10^−3^–1.0 × 102 μg/kg	0.16 μg/kg	[264]
Neonicotinic pesticides	*Lycii Fructus*	UPLC-MS–MS	Porous Boron Nitride Nanorods	8.4–100.0 μg/kg; 8.1–80.0 μg/kg; 9.2–100.0 μg/kg; 8.2–80.0 μg/kg; 9.3–100.0 μg/kg	2.2–3.7 μg/kg	[265]
Organochlorine pesticides	*Astragali Radix*	MSPE-GC–MS-MS	MFHBC	100–5000 ng/kg	8.4–278.3 ng/kg	[266]
Triadimenol Fipronil Tebuconazole Hexaconazole Diazinon	*Cinnamomi Ramulus; Scutellariae Radix*	HPLC–DAD	DES-UA-ELPME	0.05–0.5 μg/mL	0.02–0.2 μg/mL	[267]
Pyrethroids	*Ginseng Radix et Rhizoma*	Dual-Signal ITS	Luminol-Reduced AuNPs	0.2–200 ng/mL	0.067 ng/mL	[268]

Mycotoxins are toxic secondary metabolites produced by molds during cultivation, storage, or processing. Their hazards mainly include three aspects: the first is the characteristics and direct toxicity of ADME, Aflatoxin B1 (AFB1) is metabolized in the liver by CYP3A4 and CYP1A2 into epoxide metabolites (AFB1-8,9-epoxide,AFBO), which can form adducts with DNA and cause cancer [127, 128]; the second is the impact on the host's metabolic system. Various mycotoxins can induce or inhibit CYP450 and other metabolic pathways. When chronically or co-exposed, they can alter the metabolic rate and in vivo exposure of drugs and TCMs, affecting their efficacy and safety [129]; The third is the indirect effect on the active substances of TCM, which can degrade or modify the active substances and change the microbial community of the medicinal materials, thereby affecting intestinal metabolism and the absorption and bioavailability of the active substances. Mycotoxins have been listed as a limit index in the pharmacopoeia. Rhizome, and bark medicinal materials are often contaminated by soil fungi during their growth, while fruits and seeds are prone to getting damp and moldy during storage [130] In addition, the high-temperature and high-humidity environment during processing and preparation may also promote the reproduction of fungi and the production of toxins [131]. Physical, chemical and biological methods can be adopted to remove or reduce mycotoxins: post-harvest grading, crushing and rinsing can reduce mold and toxins. Frying, high-temperature steaming or extrusion molding can reduce heat-sensitive toxins. Residual toxins can be adsorbed by activated carbon during the extraction process. Biological enzymatic hydrolysis can also detoxify [132, 133]. Meanwhile, rapid and highly sensitive detection technologies such as HPLC–MS/MS, biosensors, and molecularly imprinted polymers should be combined to strengthen quality control and be promoted to complex matrices of TCM [134]. For instance, Hu et al. [135] found that the “sweating Processing”: during the *Dipsaci Radix* processing is prone to breed toxic fungi, reducing the efficacy of the medicine. Among the 63 batches of *Dipsaci Radix* by HPLC–MS/MS, 44.4% (28 batches) were contaminated with mycotoxins, and 17 batches had coexistence of multiple mycotoxins. Table 4 presents mycotoxin detection methods in TCM.

**Table 4:** Detection methods for mycotoxins in some TCMs

Analytes		TCMs	Detection method			Pretreatment-material	Linear range	LOD	Refs.
AFB1		*Citri Reticulatae Pericarpium; Bombyx Batryticatus*	Visual protein microarray			Carrier protein AgNPs	0.06–0.96 μg/kg	0.06 μg/kg	[269]
		*Ophiopogonis Radix; Paridis Rhizoma; Galla Chinensis*	ESI-UPLC-MS–MS			PANI@CS	10.0 μg/L–200 μg/L	0.1–6.0 μg/kg	[270]
		*Achyranthis Bidentatae Radix; Rehmanniae Radix; Chrysanthemi Flos; Quisqualis Fruit; Platycladi Semen*	Electrochemical aptamer sensor			BFPA-DES	2 fg/mL–2 ng/mL	0.804 fg/mL	[271]
		*Glycyrrhizae Radix et Rhizoma; Lycii Fructus; Eucommiae Cortex*	Fluorescent aptasensor			Hairpin probes DNA-AgNCs	1 × 10^−6^–1 μg/mL	0.19 pg/mL	[272]
		Red yeast rice	SPCC immunosensor array			BSA AgNPs	0.001–1 ng/mL	0.44 pg/mL	[273]
		Peanut	Fluorescent aptasensor			Self-assembled DNA double-crossover nanomachine	0.01–150 ng/mL	9.0 pg/mL	[274]
		*Lilii Bulbus; Nelumbinis Semen; Coicis Semen*	SERS-based aptasensor			Three-dimensional SPCM AuNPs	0.01–100 ng/mL	0.36 pg/mL	[275]
AFB1	*Nelumbinis Semen; Hordei Fructus Germinatus; Glycyrrhizae Radix Et Rhizoma*			QD-FLISA	PEG-functionalized CdSe-CdS QDs		0.09–0.38 ng/mL	0.05 ng/mL	[276]
	Red yeast rice			Chemiluminescence Immunosensor	HRP@AuNP-IgG		1.0 × 10^−4^–1.0 ng/mL; 1.0 × 10^−4^–1.0 ng/mL; 1.0 × 10^−4^–1.0 ng/mL	0.06 pg/mL; 0.08 pg/mL; 0.08 pg/mL	[277]
ZEN	*Citri Reticulatae Pericarpium; Bombyx Batryticatus*			Visual protein microarray	Carrier protein AgNPs		0.3–4.8 μg/kg	0.33 μg/kg	[269]
	*Coicis Semen*			Fluorescent aptasensor	Graphene oxide		1–1024 nM	0.037 nM	[278]
	*Ginseng Radix et Rhizoma*			LC–MS-MS	IAC		2.5–2500 ng/g	2.5 ng/g	[279]
	*Armeniacae Semen Amarum*			MOF Fluorescence Sensor	PVP-UiO-67		0.016–3.14 μM	7.44 nM	[280]
OTA	*Ophiopogonis Radix; Paridis Rhizoma; Galla Chinensis*			ESI-UPLC-MS–MS	PANI@CS		10.0 μg/L–200 μg/L	0.1–6.0 μg/kg	[270]
	Red yeast rice			SPCC immunosensor array	BSA AgNPs		0.001–1 ng/mL	0.83 pg/mL	[273]
	*Lilii Bulbus; Nelumbinis Semen; Coicis Semen*			SERS-based aptasensor	Three-dimensional SPCM AuNPs		0.001–10 ng/mL	0.034 pg/mL	[275]
	Red yeast rice			Chemiluminescence Immunosensor	HRP@AuNP-IgG		1.0 × 10^−4^–1.0 ng/mL	0.08 pg/mL	[277]
Analytes	TCMs			Detection method	Pretreatment-material		Linear range	LOD	Refs
OTA	*Ginseng Radix et Rhizoma*			LC–MS-MS	IAC		0.5–500 ng/g	0.5 ng/g	[279]
DON	*Citri Reticulatae Pericarpium; Bombyx Batryticatus*			Visual protein microarray	Carrier protein AgNPs		6–96 μg/kg	11.84 μg/kg	[269]
	*Ginseng Radix et Rhizoma*			LC–MS-MS	IAC		2.5–2500 ng/g	2.5 ng/g	[279]
FB1	*Citri Reticulatae Pericarpium; Bombyx Batryticatus*			Visual protein microarray	Carrier protein AgNPs		1.2–19.2 μg/kg	3.58 μg/kg	[269]
	*Ophiopogonis Radix; Paridis Rhizoma; Galla Chinensis*			ESI-UPLC-MS–MS	PANI@CS		10.0 μg/L–200 μg/L	0.1–6.0 μg/kg	[270]
CIT	Red yeast rice			SPCC immunosensor array	BSA AgNPs		0.001–1 ng/mL;	0.39 pg/mL	[273]
	Red yeast rice			Chemiluminescence Immunosensor	HRP@AuNP-IgG		1.0 × 10^−4^–1.0 ng/mL	0.06 pg/mL	[277]
Ochratoxin	*Citri Reticulatae Pericarpium; Bombyx Batryticatus*			Visual protein microarray	Carrier protein AgNPs		0.125–2 μg/kg	0.25 μg/kg	[269]
AFB2, AFG1, AFG2, FB3	*Ophiopogonis Radix; Paridis Rhizoma; Galla Chinensis*			ESI-UPLC-MS–MS	PANI@CS		10.0 μg/L–200 μg/L	0.1–6.0 μg/kg	[270]
AFM1	Peanut			Fluorescent aptasensor	Self-assembled DNA double-crossover nanomachine		0.01–200 ng/mL	6.24 pg/mL	[274]
PAT	*Coicis Semen*			Fluorescent aptasensor	Graphene oxide		5–1400 nM	2.29 nM	[278]
T-2 toxin	*Ginseng Radix et Rhizoma*			LC–MS-MS	IAC		0.5–500 ng/g	0.5 ng/g	[279]

ZEN, Zearalenone; DON, Deoxynivalenol; FB1, Fumonisin B1; AFB2, Aflatoxin B2; AFG1, Aflatoxin G1; AFG2, Aflatoxin G2; FB3, Fumonisin B3; AFM1, Aflatoxin M1; CIT, Citrinin; PAT, Patulin

Sulfur fumigation is traditionally used to preserve TCM by inhibiting enzymes and preventing spoilage. However, SO_2_ and H_2_SO_3_ can degrade active compounds and pose health risks. Especially medicinal materials are prone to oxidation and browning, insect damage and mold, and have high starch or sugar content, such as *Codonopsis Radix*, *Dioscoreae Rhizoma* and *Chrysanthemi Flos*. Moreover, the residue of SO_2_ can irritate the respiratory tract and cause liver and kidney toxicity, which is the main reason for the substandard quality of some medicinal materials [136]. For this reason, the ChP stipulates that the SO_2_ residue of more than ten kinds of TCMs such as *Codonopsis Radix* and *Dioscoreae Rhizoma* should not exceed 400 mg/kg [137]. In recent years, increasing attention has been given to non-sulfur fumigation alternatives, such as low-temperature vacuum freeze-drying and microwave or infrared dehydration, which preserve active ingredients while inhibiting mold. Additionally, improved packaging strategies, including modified atmosphere, vacuum, or nitrogen filling, have been applied to stabilize quality and extend storage duration. In addition, processing is also a good way to reduce SO_2_ residues [138]. At present, the rapid detection of SO₂ residues in TCM has become a research hotspot for ensuring the quality and safety of medicinal materials. Common methods include lead acetate test strips, fluorescent probes, HPLC/UPLC, etc. ML, which combines spectroscopy and imaging techniques, has also been widely applied [136], we will introduce it in later.

Although most plant growth regulators have relatively low toxicity, their abuse should also be taken into consideration. Because studies have found that certain plant growth regulators, such as paclobutrazol, can increase the apoptosis rate of liver cells and inhibit the proliferation of photoreceptor cells in the retina [139, 140]. Although the toxic mechanism is still unclear, the abuse of plant growth regulators will increase health risks and should be the focus of research on pollutants in TCM in the future [141].

Ensuring safe, low-contaminant raw materials is essential for subsequent quality control and efficacy optimization. Environmental contaminants not only pose direct health risks but also interfere with the ADME of active ingredients, complicating safety assessments. Sensitive, multi-residue detection methods provide technical support for effective monitoring and source risk control. Looking forward, integrating AI for data analysis, pattern recognition, and predictive modeling will be pivotal in optimizing raw material quality. Ultimately, the goal of modern TCM quality control is not only to enhance bioavailability but also to ensure efficacy under the premise of safety.

### Processing-mediated bioavailability enhancement

Processing is a pharmaceutical technique developed under the guidance of Traditional Chinese Medicine theory. Based on the characteristics of medicinal materials and clinical requirements, its core value lies in systematically optimizing bioavailability. This includes enhancing the release and dissolution of active components, reducing or transforming toxic substances, and enabling tissue-targeted delivery through adjuvants.

Processing alters the ADME behavior of TCM components by promoting the dissolution and absorption of active ingredients. For example, UPLC-Q-Exactive Orbitrap MS was used to study the PK and tissue distribution of *Rhei Radix et Rhizoma* and its charcoal-processed form in a mouse model of ulcerative colitis. The charcoal form significantly reduced Tmax, increased AUC, C_max_, and T_1/2_ of five free anthraquinones, and decreased clearance and MRT_0-t_. Tissue distribution revealed earlier and higher accumulation in 0.25–1.25 h, compared to 4–6 h in the raw form. These results confirm the scientific basis of the traditional concept of “charcoal to stop bleeding” by facilitating the rapid release of procoagulant anthraquinones [142]. Processing also affects compound compatibility in formulas. In the Qixueshuangbu formula, several TCMs were stir-fried with yellow wine or steamed with black bean juice. This significantly increased the C_max_ and AUC of ginsenosides Rb1, Re, Rg1, ferulic acid, astragaloside IV, and paeoniflorin. Certain compounds also showed altered T_1/2_ and MRT_0-t_, suggesting that adjuvants modulate their metabolic stability and release profile [143]. Another key goal of processing is toxicity reduction without compromising efficacy. For example, stir-frying *Strychni Semen* at 230–240℃ for 3–4 min breaks ether bonds in strychnine and brucine, forming isomers and N-oxides with lower toxicity approximately 1/10 to 1/15 of the original compounds, while retaining pharmacological effects [144]. This exemplifies the Traditional Chinese Medicine concept of “reducing toxicity while preserving efficacy”. Processing also helps reduce SO_2_ residues. Sulfur fumigation of *Codonopsis Radix* often exceeds ChP residue limits. However, honey-processing or rice stir-frying after fumigation significantly lowers SO_2_ levels in decoction pieces, with honey-processed samples meeting regulatory standards even after 90 min of fumigation [145]. Adjuvants used during processing can facilitate tissue-specific distribution. For instance, salt-processed *Morindae Officinalis Radix* showed enhanced absorption of monotropein (Tmax reduced from 1.0 h to 0.5 h) and increased C_max_ and AUC_0-t_ of alizarin, rubiadin, and rubiadin 1-methyl ether. These compounds accumulated more in the small intestine, illustrating the PK basis for the traditional theory of “guiding drugs to the kidney” [146].

Modern processing focuses on precision and efficiency. Novel techniques such as high-pressure steaming, puffing, and freeze-drying have replaced empirical methods. For example, *Ginseng Radix et Rhizoma* treated with *Lactobacillus plantarum* fermentation and high-pressure expansion showed a 47-fold increase in rare saponins (Rg3, Rg5, Rk1), enhanced antioxidant activity, and improved intestinal absorption, as verified by rat intestinal capsule models [147]. These innovations overcome the bioavailability bottleneck of traditional ginsenosides (< 5%). Fermentation, germination, and defrosting are also being explored to optimize compound release and bioavailability [148].

Processing has now entered the era of integrated production. Automated systems streamline cleaning, moistening, cutting, processing, and drying into continuous lines particularly beneficial for bulk TCM. Some large equipment enterprises have established various specialized production lines for seeds, small fruits, flowers, whole plants and roots and stems [149]. The modernization of TCM processing is shifting from single-factor control to multi-factor integration [150]. Among them, Intelligent technologies such as electronic eyes and tongues simulate human perception to quantify color, shape, taste, and aroma, enabling standardized classification and real-time quality monitoring [151]. For example, electronic nose analysis during the “nine steaming and nine sun-drying” of *Rhei Radix et Rhizoma* revealed significant odor changes after the sixth cycle. Inorganic sulfur compounds peaked at that point, and the odor profile of the final product differed significantly from both raw and single-steamed forms. This study provides a scientific explanation for the traditional belief in the unique efficacy of this processing method [152].

Processing plays a key role in enhancing the bioavailability of TCM and reflecting Traditional Chinese Medicine holistic philosophy. However, challenges persist, including outdated techniques, lack of standardized procedures, and reliance on empirical practices [153]. Addressing these issues requires a shift toward precision processing guided by the principle of “enhancing efficacy and reducing toxicity”. Future efforts should integrate multidisciplinary innovations to establish a processing quality control system rooted in bioavailability. By applying multi-omics technologies and component-effect network analysis, it is possible to quantitatively link processing parameters with quality attributes and clinical outcomes. This approach will support the development of a modern and standardized processing framework with controllable safety margins and predictable therapeutic effects.

### Application of nanomedicine in TCM preparations

The optimization of modern TCM preparations requires overcoming bioavailability barriers through multiple strategies. Challenges include low solubility, poor stability, limited membrane permeability, structural incompatibility, and rapid metabolism of active components. To address these issues, researchers have proposed classification frameworks such as the biopharmaceutics classification system of Chinese materia medica [154] and the biopharmaceutics classification system of CMM component (BCSCC) [155]. Based on these systems, TCM dosage forms have evolved from traditional preparations to modern forms with improved molecular properties. Traditional dosage forms (such as pills, powders, and ointments) often require multiple administrations and have slow onset of action. Modern dosage forms, including granules and injections, aim to enhance absorption and therapeutic efficiency. For instance, the lipophilic compound tanshinone IIA from *Salviae Miltiorrhizae Radix et Rhizoma* was modified through sulfonation to increase its water solubility. It was then developed into an injectable form to bypass gastrointestinal absorption and first-pass metabolism. Sodium tanshinone IIA sulfonate is now widely used in clinical practice in China [156].

Modern preparation technologies aim to improve solubility, permeability, or both simultaneously, thereby enhancing oral bioavailability. In particular, nanotechnology plays a increasingly important role in overcoming physicochemical limitations of TCM molecules [157]. Nano-TCM preparations also offer advantages in targeted delivery, toxicity reduction, and sustained release, making them a shows potential direction in modern TCM development [158].

#### Improve the solubility of TCM preparations

Solubility enhancement technologies, such as amorphous solid dispersions (ASDs), emulsions, inclusion complexes, nanomicelles, and protein-based delivery systems, have shown great potential in improving the bioavailability of poorly soluble TCM ingredients. These strategies aim to overcome barriers such as poor solubility, high crystallinity, and rapid metabolism.

ASDs involve dispersing poorly soluble drugs with carriers to form amorphous solid mixtures, thereby improving solubility and dissolution rates by altering the crystalline structure [159]. For example, quercetin exhibits anti-ulcer activity but suffers from low solubility and rapid elimination. Researchers developed raft-forming gastric retention formulations using povidone K30. Both the liquid and chewable ASD tablets floated within 1 min and remained buoyant for over 24 h in acidic media. The optimized liquid formulation showed strong raft strength (10.4 g) and sustained release (93% within 8 h) [160]. Tanshinone IIA, known for its strong crystallinity and poor solubility, was combined with sodium alginate to form ASDs. The carrier inhibited crystal growth and significantly enhanced solubility and anti-inflammatory activity in vitro [161]. Furthermore, most TCM preparations are metabolically destroyed before reaching the colon, resulting in low drug concentration at the lesion site and poor therapeutic effect. The emergence of enteric-coated ASDs is a powerful tool for the intestinal targeted release of TCM preparations [162].

Emulsions are systems of immiscible liquids stabilized by surfactants. Microemulsions (10–100 nm) and nanoemulsions (100–300 nm) differ in droplet size and formation mechanisms*.* A microemulsion of *Chuanxiong Rhizoma*-*Angelicae Sinensis Radix* pair is effective in anti-inflammatory, analgesic and antipyretic. A combination of *Chuanxiong Rhizoma* and *Angelicae Sinensis Radix* volatile oils (CA-VO) was developed for treating acute lung injury (ALI). The formulation significantly increased oral bioavailability and improved pharmacological outcomes, including reduced lung index and prolonged survival in mice [163]. Nanoemulsions, by encapsulating drugs in nanoscale droplets, not only enhance the solubility of drugs but also reduce the metabolism of unstable active substances [164]. Berberine is a typical substance with poor solubility, poor permeability, fast intestinal metabolism and P-gp excretion. It is prone to be metabolized by primary oxidative demethylation reactions in the intestinal mucosa, affecting oral bioavailability [165]. The preparation of berberine into nanoemulsion (BBR-NE) can reduce its contact with metabolic enzymes and significantly improve its stability and membrane permeability. Caco-2 cell transport and in vivo intestinal perfusion revealed that BBR-NE reduced the exopostery ratio from 18.17 of BBR to 7.21 and increased permeability by 1.78 times. The C_max_ of BBR-NE increased from 88.57 µg/L to 113.70 µg/L, the T_max_ shortened from 0.43 h to 0.23 h, the AUC_0-t_ increased by 212.02%, and the T_1/2_ prolonged by 327%. The hypoglycemic effect of BBR-NE was superior to metformin, attributed to its triple mechanisms of anti-metabolism, anti-excretion, and permeation enhancement [166].

Inclusion complexes, particularly those using cyclodextrins, encapsulate hydrophobic drugs within hydrophilic cavities to improve solubility and stability [167]. Sun et al. [168] found that ginsenoside Rg3 can induce immunogenic cell death (ICD) in colorectal cancer, and quercetin enhances the ICD effect through reactive oxygen species. The nano-formulation based on folic acid-targeted cyclodextrin achieved co-delivery of the two drugs, with the optimal molar ratio (1:1), prolonged t₁/₂ to 1.3–1.4 h, significantly improved circulation time and tumor targeting without obvious toxicity. Combined with anti-PD-L1 therapy, it significantly prolonged survival in colorectal cancer models, demonstrating the utility of nanoinclusion systems in immunochemotherapy.

Nanomicelles consist of hydrophobic cores and hydrophilic shells, ideal for solubilizing anticancer drugs. Baicalin nanomicelles (BLNM) constructed with stearic acid modified pluronic F68 achieved 84.3% encapsulation efficiency and 97% drug retention over 48 h. They significantly enhanced baicalin’s solubility, stability, and cytotoxicity against A549 lung cancer cells [169]. Similarly, there are hybrid micelles, such as the development of an SK/siIDO1-HMs hybrid micelle for co-delivery of shikonin and IDO-1 siRNA, which was experimentally demonstrated to have a good cycling time, rapid intracellular release, and a broad therapeutic scope [170].

Protein-based systems provide biocompatibility, biodegradability, and intrinsic targeting ability. HSA is widely used in TCM nanomedicine [171], it can significantly optimize drug stability and PK properties [172]. Xu et al. [173] discovered that piperine, as a natural P-gp inhibitor, can enhance the anti-tumor sensitivity of paclitaxel, and developed co-loaded albumin nanoparticles to improve solubility and targeting. Piperine reduced paclitaxel IC_50_ by up to 9.14-fold and synergistically inhibited tumor growth while reducing toxicity. Silk fibroin and zein are also gaining attention. Among them, silk fibroin is mostly used in nanoparticle, gel and other drug delivery systems to achieve controllable release, enhance tissue adhesion and wound regeneration, etc. [174]. Such as, Qu et al. [175] prepared apicin nanospheres (with a drug loading rate of 6.20% and heat resistance up to 260℃) by taking advantage of the good biocompatibility and drug loading characteristics of silk fibroin protein, significantly enhancing the inhibitory effect on 4T1 and MDA-MB-231 cells. With free apigenin, nanospheres increased blood drug concentration, prolonged T_1/2_ (from 1.51 h to 2.93 h), increased AUC_0-t_ (from 20.50 to 30.18 μg/mL·h), reduced CL value from 27.25 to 11.50, and prolonged MRT from 3.81 h to 4.06 h. Zein is widely used in gastrointestinal protection, enhancing intestinal absorption and improving bioavailability. It is suitable for oral administration or local delivery of TCM extracts or weakly stable components [176]. Fu et al. [177] prepared hybrid nanoparticles of notoginsenoside using zein and lecithin. Its permeability was 1.5 times higher than that of free notoginsenoside, and the absorption parameters in the ileum and jejunum were increased by 1.75 times and 1.80 times, respectively. Its relative oral bioavailability was 1.71 times that of free notoginsenosides, indicating that this nanoparticle can enhance the stability and absorption efficiency of notoginsenosides in the gastrointestinal tract, thereby significantly improving bioavailability.

Although solubilization technologies significantly improve dissolution, bioavailability is still limited by cellular membrane barriers. Therefore, combining solubility enhancement with permeability-promoting strategies is essential to maximize therapeutic efficacy.

#### Improve the permeability of TCM preparations

When the active substance has sufficient solubility, permeability becomes a key constraint on absorption. The following infiltration promotion techniques are often used in conjunction with solubilization processes.

Liposomes are vesicles composed of phospholipid bilayers that can encapsulate both hydrophilic and hydrophobic compounds. Their structural similarity to cellular membranes grants them excellent biocompatibility and low immunogenicity [178]. For example, comfreyin has potent antitumor effects via immunogenic cell death but suffers from poor solubility and low therapeutic index [179]. Researchers developed co-loaded liposomes combining viologen with mitoxantrone or adriamycin, which showed improved drug ratio delivery, enhanced tumor inhibition, and a better safety profile in vivo [180]. Hydrophilic salvianolic acid B (SAB) is embedded into the lumen of liposomes modified with polyethylene glycol (PEG) to create a drug delivery system named PEG-SAB-Lip, which extends the biological half-life of SAB in the body. Experiments have shown that when SAB accumulates at the tumor site, it can reverse the tumor microenvironment by converting M2 macrophages into M1 macrophages and inhibiting Tregs infiltration [181]. LipoMicel® Berberine (LMB), a lipid micelle formulation using phosphatidylcholine and medium-chain triglycerides, significantly improved berberine solubility and absorption. Solubility increased 1.4-fold in water and 22-fold in simulated gastric fluid, while Caco-2 permeability was 14.6 times higher than conventional berberine. In a randomized, double-blind, cross-designed registered clinical trial (n = 10 healthy volunteers): compared with conventional berberine preparations, a single oral administration of 500 mg LMB increased AUC_0–24_ by 5.8 times, C_max_ by 9.5 times, and T_max_ by approximately 5 times. There was no statistically significant difference between men and women, and no adverse reactions occurred. This research provides a feasible example for the clinical transformation of TCM molecular delivery systems [182].

Phospholipid complexes improve gastrointestinal absorption by enhancing lipophilicity through non-covalent interactions. For instance, a phospholipid complex of total alkaloids (TA) from *Sophorae Flavescentis Radix* increased the oil–water partition coefficient and significantly improved oral bioavailability [183]. The experiment found that, compared to the simple TA, the relative bioavailability of oxymatrine, matrine, sophocarpine, and oxidized sophorin in the phospholipid complex increased to 161.33%, 134.82%, 185.94%, and 397.36% respectively after gavage. Meanwhile, the T_1/2_ and MRT_0-t_ of these four components were also extended [184].

Self-assembly refers to the spontaneous formation of nanostructures (such as micelles or nanoparticles) through intermolecular interactions among TCM components. The Chinese Medicine Self-Assembly Nanostrategy (CSAN) aims to improve solubility, targeting, and therapeutic efficacy, especially in oncology [185]. For instance, glycyrrhizic acid and hydroxycamptothecin self-assembled into micelles with enhanced solubility and antitumor activity [186]. In another case, tanshinone IIA and glycyrrhizic acid formed nanomicelles coated with serum exosomes and CpG oligonucleotides. This system can bind to free transferrin in the blood to prolong the circulation time and remain stable when crossing the BBB and targeting glioblastoma. When combined with temozolomide, improved therapeutic outcomes and reduced recurrence [187]. In addition, the phenomenon is also very common in formulas. Such as, NPs composed of polysaccharides in Huanglian decoction [188] promote the absorption of berberine in the intestinal tract, NPs in Baishaogancao decoction promote in vitro release and intestinal absorption of insoluble components of *Glycyrrhizae Radix et Rhizoma* [189]. Processing can also induce self-assembly. Vinegar-frying *Bupleuri Radix* leads to the formation of bupleurum polysaccharide micelles with small size, positive charge, and flexible conformation, enhancing immune cell uptake [190]. Similarly, frying *Epimedii Folium* in mutton fat promotes the self-assembly of icariin into stable nanomicelles. These structures improve icariin permeability and encapsulation in the rat duodenum, enhancing solubility and absorption [191]. These phenomena support the scientific basis of Traditional Chinese Medicine compatibility and the theory of “enhancing efficacy and reducing toxicity”.

**Fig. 4:**
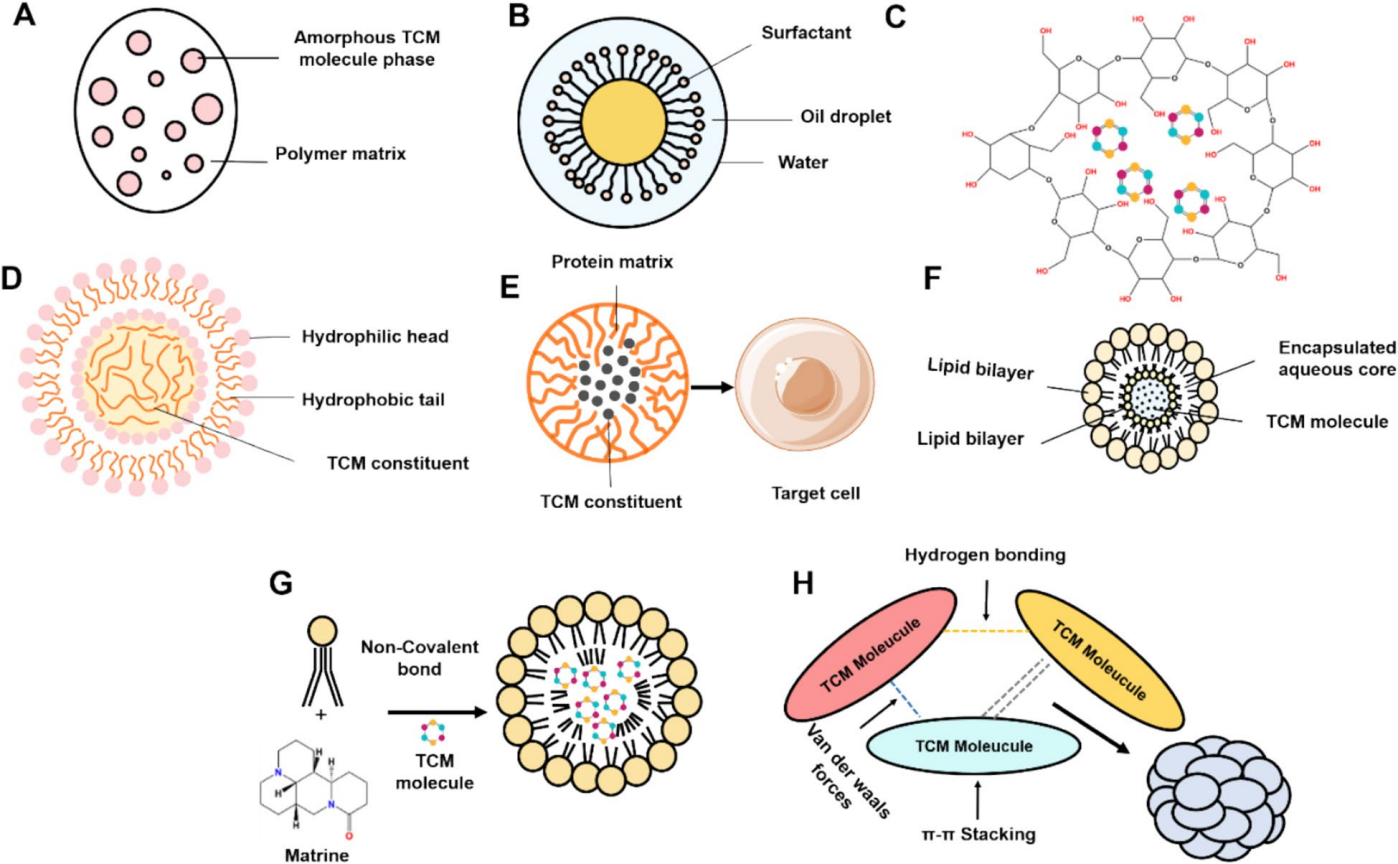
Traditional Chinese medicines NDDS. **A**. Amorphous solid dispersions. **B**. Nanoemulsion. **C**. Inclusion complexes made of cyclodextrin. **D**. Nanomicelle. **E**.Protein nanoparticles. **F**. Liposome. **G**. Phospholipid complexes. **H**. Chinese medicine self-assembled nanoparticle

Nanotechnology provides a modern scientific framework for interpreting Traditional Chinese Medicine theories and offers promising avenues for formulation innovation. However, despite its potential to enhance drug delivery, therapeutic efficacy, and mechanistic understanding, most nano-TCM technologies remain at the preclinical stage. Clinical translation is hindered by unresolved challenges, including: (1) long-term toxicity profiles of nanocarriers (e.g., polymer accumulation, immune responses) require comprehensive assessment. (2) batch-to-batch consistency in complex TCM nanopreparations demands advanced quality by design (QbD) approaches. (3) Specific guidelines for nanomedicine evaluation add complexity to approval processes. Addressing these challenges through collaborative efforts in data generation, standardized methodologies, robust validation, and proactive engagement with regulatory agencies is essential before nano-TCM can fulfill its promise and play a pivotal role in TCM modernization. Figure 4 shows the above-mentioned nanoformulations.

#### AI-nanomaterial collaborative design

In recent years, AI has advanced from conceptual validation to practical application in nanocarrier design and formulation optimization. ML and DL models have been applied to predict key formulation parameters such as particle size, encapsulation efficiency, in vitro release profiles, and toxicity, significantly reducing experimental workload and improving design efficiency.

For instance, the NanoMine database [192, 193], through cross-experimental integration and ML modeling, revealed that the glass transition temperature of polymer nanocomposites is primarily influenced by nanofiller volume fraction and interfacial surface energy. This provides quantitative, data-driven guidance for material design. In another study, RF and light gradient boosting machine algorithms identified nanoparticle concentration, diameter, and zeta potential as key factors influencing toxicity, leading to the proposal of safety design guidelines (e.g., diameter > 50 nm and neutral zeta potential) for nanomaterials [194]. AI has also been used to analyze drug release data, predict optimal drug-to-carrier ratios, and model release kinetics under various conditions [195, 196]. These tools have been applied to optimize the design of lipid nanoparticles, liposomes, solid lipid nanoparticles, and polymeric carriers [197].

Recently, high-throughput platforms have emerged that combine microfluidics, high-content characterization, and active ML. A closed-loop optimization platform for nanoparticle design was developed, which increased the uptake efficiency of Polylactic acid-glycolate (PLGA)-PEG nanoparticles by threefold in breast cancer cells within two weeks. The system rapidly identified formulations that met multiple requirements (particle size, stability, drug loading, and release profile), thus accelerate the transition from screening to optimization [198]. These AI-driven strategies are increasingly being explored in the development of TCM-based nanomedicines. Current applications focus on two main directions: using AI to identify active TCM ingredients, predict targets and toxicity, and match them with suitable nanocarriers; using AI directly to optimize nanoformulation parameters. In cancer treatment, AI-TCM integration has shown particular promise. For example, Xia et al. [199] combined DL with experimental screening to develop a geometric perception neural network, identifying ilexgenin A as a dual-functional natural product with lipid membrane modulation and GLUT1 targeting properties. Liposomes co-loaded with ilexgenin A significantly enhanced antitumor efficacy, increasing tumor accumulation by 60%, prolonging T_1/2_ by 2.5 times, and reducing hepatosplenomegaly toxicity.

Although AI applications in TCM nanomedicine offer promising avenues for accelerating formulation design and optimization, significant translational barriers remain. The predictive power of AI models depends heavily on high-quality, standardized datasets, yet the intrinsic complexity of TCM—characterized by multi-component systems and phytochemical variability—poses substantial challenges to data integration and model generalizability. Moreover, in silico predictions must be rigorously validated through in vitro, in vivo, and ultimately clinical studies to bridge the gap between computational outcomes and real-world efficacy. Regulatory frameworks for AI-assisted nanopreparations are still evolving, requiring evidence of manufacturing reproducibility, long-term safety, and immunocompatibility. Therefore, while AI holds transformative potential, its successful application in TCM nanomedicine hinges on multidisciplinary collaboration, robust experimental validation, and proactive regulatory engagement.

### Bioavailability-oriented synergistic strategies

In Traditional Chinese Medicine, compatibility governs multi-component interactions under the guidance of syndrome differentiation, generating synergistic effects beyond the sum of individual herbs. At the heart of this lies the Jun-Chen-Zuo-Shi (monarch-minister-assistant-courier) framework. This is not a rigid role assignment but a dynamic functional hierarchy: the monarch TCM (jun) provides the principal therapeutic effect (e.g., ginsenosides in Shengmai yin) [103]; the minister TCM (chen) synergistically overcome bioavailability barriers (e.g., *Schisandrae Chinensis Fructus* lignans in Shengmai yin [103]; the assistant(zuo)/courier(shi) agents optimize ADME processes. For example, *Borneolum Syntheticum* increases vascular permeability and modulates membrane transport to enhance drug distribution [200].

This hierarchical strategy influences TCM bioavailability by modulating HHI. Compatibility principles are further governed by three classical frameworks: (1) the seven compatibility relationships, which define synergistic, antagonistic, and neutral interactions (single medicine, mutual reinforcement, mutual assistance, mutual restraint, mutual suppression, mutual aversion, mutual incompatibility); (2) the eighteen incompatibilities, which warn against specific herb pairs due to potential toxicity; (3) the nineteen antagonisms, which identify combinations that reduce efficacy or cause adverse reactions. These traditional principles align with modern pharmacological concerns such as CYP450 inhibition or induction [201], demonstrating the empirical foresight of traditional HHI warnings. Classic assistant and courier TCMs like *Glycyrrhizae Radix et Rhizoma* [202] and *Borneolum Syntheticum* [203] exemplify how compatibility optimizes ADME. These adjuvants enhance the bioavailability of monarch components by influencing parameters such as membrane permeability, metabolic stability, and tissue distribution.

With the modernization of TCM, compatibility is increasingly interpreted through the lens of component-efficacy relationships. This has led to the development of component-based TCM preparations built from isolated active compounds or defined chemical groups [204], representative approaches include the BCSCC and innovative structure-based formulations, which improve solubility and permeability to enhance gastrointestinal absorption. Examples include the TA-phospholipid complex from *Sophorae Flavescentis Radix* [184], as well as standardized compound formulas like Guanxinning tablets [205], and Zuojin pills [206]. These represent the integration of compatibility theory with modern pharmaceutical design.

**Fig. 5:**
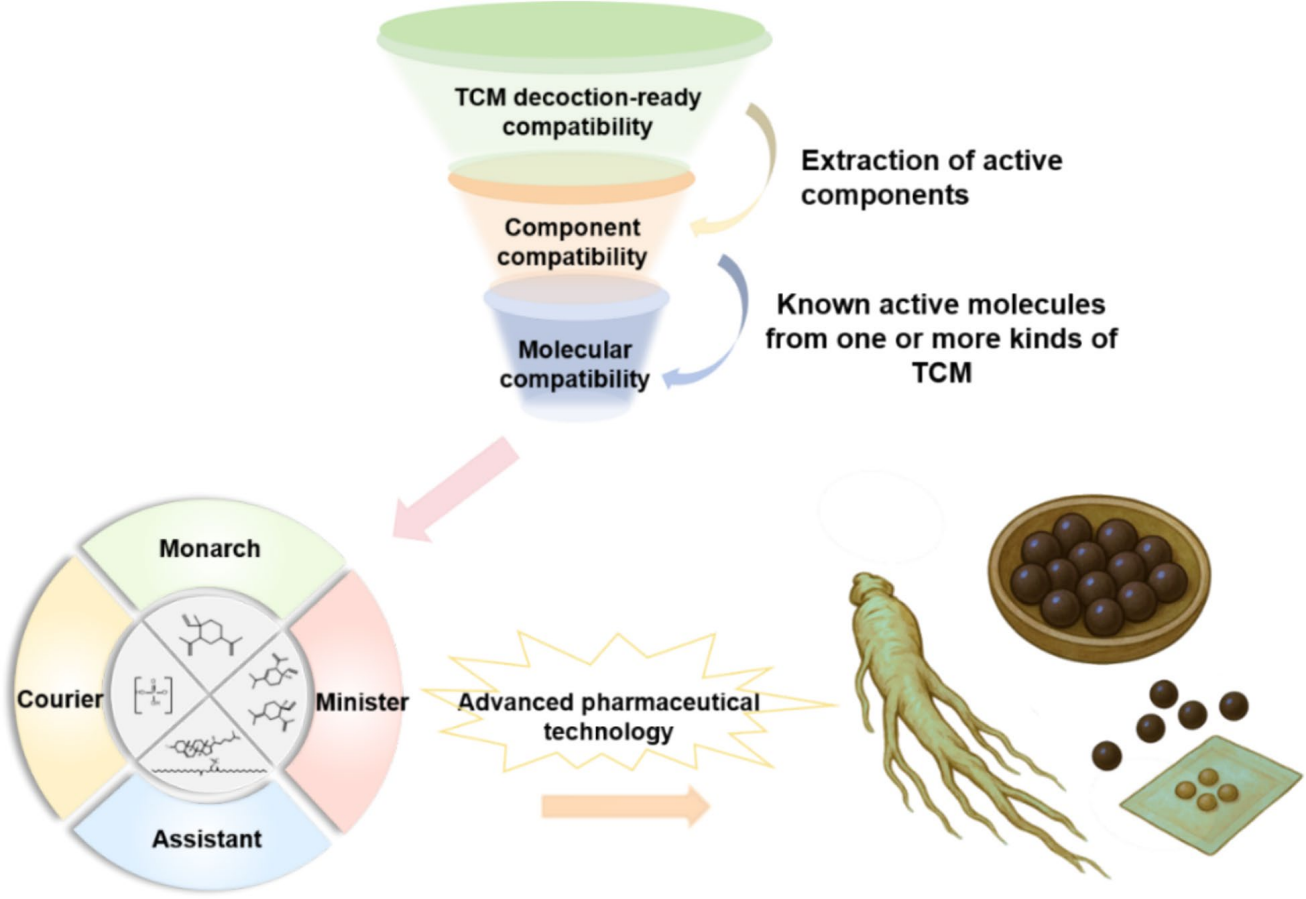
From TCM synergy to functional hierarchies: the evolution of TCM compatibility

Molecular compatibility reflects Traditional Chinese Medicine’s holistic concept at the systems pharmacology level, where structurally distinct bioactive compounds self-assemble into synergistic alliances that align with the traditional monarch-minister-assistant-courier framework [207]. A representative example is the development of elemene-based antitumor drugs. Elemene, extracted from *Curcumae Rhizoma*, exhibits poor water solubility and low bioavailability. Dr. Tian Xie et al. [208] revealed significant synergistic effects among three elemene isomers (β-, γ-, and δ-elemene) in antitumor efficacy and safety. Although β-elemene (accounting for 85%) shows the strongest individual activity, the natural isomeric mixture (β:γ:δ = 85:15) produced superior therapeutic efficacy and safety compared to any single component. Mechanistically, β-elemene plays the role of the monarch component, directly inducing tumor cell apoptosis by downregulating Bcl-2 and survivin, and upregulating Fas/FasL expression. γ- and δ-elemene function as minister components, enhancing tumor penetration, overcoming multidrug resistance, and amplifying the therapeutic effect of β-elemene. These isomers also inhibit DNA/protein synthesis, promote differentiation and apoptosis, and reverse drug resistance [209], resulting in a synergistic effect greater than the sum of individual actions. To address solubility and bioavailability challenges, PEGylated liposomes were developed using a PEG-soy lecithin-cholesterol complex, which acts as the assistant/courier component in the delivery system. These liposomes improved PK parameters: CL decreased by 1.75-fold, half-life (T_1/2_) increased by 1.62-fold, and AUC_0–1.5.5_ increased by 1.76-fold. Furthermore, transferrin-modified biomimetic liposomes enabled BBB penetration and prolonged survival in glioblastoma-bearing mice. This multi-level organization from molecular synergy to delivery system design translates Traditional Chinese Medicine holistic therapeutic concept into quantifiable PK/PD outcomes, significantly enhancing the bioavailability and efficacy of β-elemene [17]. Molecular compatibility not only preserves the integrative spirit of Traditional Chinese Medicine but also bridges traditional theory with modern science. It enables mechanistic interpretation and standardization, providing a structure-based strategy for the modernization of TCM. Figure 5 illustrates the hierarchical compatibility model in TCM and its correspondence with the “Jun-Chen-Zuo-Shi” principle.

Molecular compatibility bridges the holistic principles of Traditional Chinese Medicine with modern evidence-based medicine by enabling quantifiable pathway modulation. Numerous studies support this integration. For example, in *Salviae Miltiorrhizae Radix et Rhizoma*, a 13.4:1 mass ratio of salvianolic acid B (monarch) to tanshinone IIA (minister) significantly regulated key pathways: it activated *p*-PI3K/PI3K and *p*-Akt/Akt, while inhibiting *p*-NF-κB/NF-κB, thereby improving renal inflammation more effectively than either component alone [210]. Similarly, in a systems pharmacology analysis of Banxiabaizhutianma decoction, *Pinelliae Rhizoma* (stigmasterol) and *Gastrodiae Rhizoma* (gastrodin) were confirmed as the monarch TCMs for treating phlegm-dampness hypertension [211]. Their combination modulated lipid metabolism via the AMPK/SREBP-1C pathway, achieving superior lipid-lowering effects compared to individual ingredients. These findings reflect a central tenet of Traditional Chinese Medicine: functional integration among components creates emergent therapeutic effects that individual agents cannot produce alone.

Traditional Chinese-Western Medicine combinations can enhance efficacy through targeted PK modulation, a form of HDI. While some HDIs are beneficial, others pose safety risks, highlighting the need for careful evaluation. For instance, baicalein, a flavonoid from *Scutellariae Radix*, significantly inhibits CYP3A4 in a dose-dependent manner. When combined with simvastatin, it increases the drug’s exposure by threefold and prolongs its metabolic time, enhancing therapeutic efficacy [212]. However, not all combinations are safe. *Angelicae Dahuricae Radix* contains furanocoumarins that inhibit hepatic drug-metabolizing enzymes. When co-administered with nifedipine, this interaction raises plasma drug levels and increases the risk of adverse effects [213]. Therefore, rational drug compatibility should be guided by both functional synergy and quantitative monitoring, while also considering individual differences and classical contraindications to ensure safety and efficacy.

TCM compatibility has evolved from crude herb combinations (decoction pieces) to molecular-level integration, while retaining its core principle: therapeutic effects arise from coordinated functional interactions. This evolution is now supported by a three-pronged strategy: (1) bioavailability-oriented design: enhancing solubility, permeability, and metabolic stability to optimize ADME performance; (2) quantification of pathway synergy: using network pharmacology to analyze monarch-minister ratios and validate compatibility rationale; (3) risk control: identifying and avoiding harmful HHI and HDI to ensure safety. Looking ahead, the traditional Jun-Chen-Zuo-Shi (monarch-minister-assistant-courier) system could be transformed into a programmable functional hierarchy: major active ingredients act as therapeutic cores; enhancement modules improve delivery and efficacy; detoxifying carriers reduce toxicity, and targeting units direct effects to specific tissues. This approach will help shift Traditional Chinese Medicine from empirical compatibility toward computationally driven precision medicine, accelerating its modernization and scientific validation.

### Quality evaluation of TCM

The quality evaluation of TCM and its preparations ensures intrinsic quality and clinical efficacy by assessing authenticity, composition, content, consistency, safety, and effectiveness. It plays a central role in safeguarding the bioavailability of TCM. At the core of this process lies the development of evaluation methods and the establishment of a comprehensive quality system. Currently, the advancement of AI-driven evaluation methods and the construction of “Q-Marker” centered quality systems are providing scientific support for improving and maintaining the bioavailability of TCM.

#### AI-driven quality evaluation methods for TCM

Current strategies are evolving from single-indicator assessment to integrated, multidimensional systems, from static detection to dynamic monitoring, and from experience-based judgment to standardization and internationalization. In particular, AI-driven multimodal technologies are narrowing the gap between traditional quality control and clinical outcomes [214].

AI has been increasingly applied across multiple stages of TCM production, including ecological suitability analysis, germplasm identification, processing optimization, authenticity verification, and preparation development. For instance, Yang Y et al. [215] used the MaxEnt model combined with HPLC and chemometrics to predict suitable planting zones for *Zanthoxyli Radix* and showed that ecological factors significantly influence the accumulation of its active alkaloids. Bai et al. [216] applied five ML/DL models to hyperspectral imaging data for *Coicis Semen* and demonstrated that the CARS-ResNet model could accurately determine its storage age. Odor, an important indicator of TCM quality, is gaining attention, although the mechanism of “qi discrimination for quality assessment” is still unclear at present, research in this area has gradually become popular. Yao et al. [217] used an electronic nose to differentiate *Angelicae Dahuricae Radix* from different sources. Combined with chemometrics, network pharmacology, and molecular docking, they established a correlation between odor, active components, and efficacy supporting a quality control model based on the “odor-component-efficacy” relationship. Authenticity identification is critical for safety and efficacy. Liu et al. [218] constructed a barcode reference database for the genus *Uncaria* using 613 sequences. They demonstrated that ITS/ITS2 barcodes, combined with BLOG and WEKA-SMO classifiers, achieved 100% identification accuracy, outperforming traditional methods and offering strong support for standardization. AI also contributes to processing optimization. For example, Liu et al. [219] studied the honey-frying process of *Mori Cortex*. Under optimal conditions (80 °C, 100 min), honey-frying produced 5-hydroxymethylfurfural and altered flavonoid content. Using ANN-assisted screening, network pharmacology, and molecular docking, they identified resveratrol, kuwanon G, and moracenin as key anti-inflammatory markers. This established the model of “chemical profiling-AI screening-activity verification”, providing theoretical support for standardizing processing techniques. To monitor the processing degree of *Epimedii Folium*, a predictive model was developed using electronic eyes, near-infrared spectroscopy, CNN, and partial least squares regression. The CNN model reached 93.3% accuracy in classification. The regression model achieved high predictive performance for four key flavonoids (R^2^ > 0.90, RPD > 4) [220].

In pesticide residue control, AI has been used to assess environmental impact. QSAR models built using genetic algorithms and bayesian optimization linked pesticide structures with acute toxicity in earthworms [221]. For mycotoxin detection, DL models such as sub-pixel convolutional networks have been used to quantify AFB1 in peanuts [222, 223]. Ma et al. [224] coupled a whole-cell biosensor array with RF models to identify moldy peanuts and corn, achieving 83% accuracy. AI is also useful for sulfur fumigation detection. In the case of *Zingiberis Rhizoma Recens*, image-based prediction models using SVM, BP-ANN, and RF were developed. A weighted voting fusion strategy based on model accuracy achieved 80%−100% prediction accuracy [225].

Despite rapid progress, AI-based TCM quality evaluation still faces several challenges: the diverse components and preparation methods of TCM complicate data standardization and acquisition; the synergy between AI technologies and Traditional Chinese Medicine theory remains underexplored; AI should support rather than replace the goal of establishing robust quality standards rooted in Traditional Chinese Medicine theory. Future research should focus on high-quality data infrastructure, deep integration of multimodal AI with traditional methods, and the development of an intelligent, diversified, and standardized quality control system. Under the guidance of Traditional Chinese Medicine theory, such systems will enable scientific, accurate, and internationally aligned evaluation of TCM quality.

#### Quality evaluation system

Establishing a complete and advanced evaluation system is essential for ensuring consistency, safety, and clinical efficacy. In recent years, several quality evaluation concepts tailored to Traditional Chinese Medicine characteristics have emerged, among which the Q-Marker has become a core standard.

Q-Markers refer to inherent or process-derived chemical substances in raw or prepared TCM products that are closely linked to efficacy and safety [226]. They are designed to address the limitations of traditional quality standards and improve consistency, traceability, and controllability. Using the five key elements of Q-Marker——“fingerprint components-process reproducibility”, “biology-efficacy and safety”, “TCM efficacy-mechanism correlation”, “measurability of quality substances”, and “stability of quality standards” as the main focuses, a multidisciplinary integration has formed a multi-dimensional information integration Q-Marker comprehensive analysis research pathway, such as the five-principle Q-Marker research pathway and the metabolomics Q-Marker research pathway [227]. The five principles refer to “quality transfer and traceability”, “component specificity”, “component efficacy”, “component measurability”, and “compatibility environment in compound formulas”. Based on the “five principles”. For instance, Wang et al. followed the five principles of Q-markers and systematically revealed the Q-markers of Qingzaojiufei decoction (QZJFD) in resisting ALI based on the strategy of “network pharmacology-metabolomics-PK-PK/PD modeling”. A total of 121 components were identified in vitro, 33 in vivo prototypes and 20 candidate components, and a ternary network was constructed to reveal their effects on inflammation, oxidative stress and metabolic pathways. Metabolomics identified 35 differential metabolites and verified pathways in five types of biological samples. Comparative PK showed that the PK of the 18 components changed significantly under the ALI model. By integrating the correlation and factor analysis of PK-PD, nine Q-markers were finally screened out the monarch TCM is chlorogenic acid in *Mori Folium*, the minister TCM are methylophiopogonanone A and methylophiopogonanone B in *Ophiopogonis Radix*, and the assistant TCM are *Sesami Semen Nigrum* in sesamin, ursolic acid in *Eriobotryae Folium,* amygdalin in *Armeniacae Semen Amarum*, the courier TCM are liquiritin apioside, liquiritigenin, isoliquiritin in *Glycyrrhizae Radix et Rhizoma*. This research not only systematically reveals the material basis of QZJFD’s resistance to ALI, but also provides a scalable paradigm for the transformation of TCM quality control from chemical composition to overall biological effects [228]. Similarly, there are *Pinellia Ternata* [229], *Cuscutae Semen* [230], Buyanghuanwu decoction [231] and so on. Despite the fragmentation of Q-Marker and the lack of standardization of selected ingredients, the multi-dimensional integration pattern presented by Q-Marker is an idea needed for quality control of TCM [232].

In recent years, the whole process quality control strategy has been put forward in the field of quality control of TCM. Starting from the whole process, this strategy systematically links raw Chinese medicines, decoction-ready medicines and preparations to show the whole process transitivity of the quality of TCM. This covers not only the quality transfer from raw materials to clinical, but also the quality traceability from clinical to raw materials, which is consistent with the concept of transferability and traceability of Q-Marker [233]. Therefore, the combination of Q-Marker and the whole process quality control strategy has become the main direction of the current research on the quality control of TCM. Among them, the concept of QbD [234] is introduced into the whole process quality control of TCM. By setting the outline of product quality objectives, identifying key quality characteristics, defining key processes and key process parameters, it not only ensures the efficacy, safety and quality stability of TCM products, but also provides a systematic and efficient management method for the optimization of bioavailability of TCM. Here we take Xuesaitong injection (XST) as an example to introduce the implementation of the whole-process quality control strategy. This is a TCM injection derived from the total saponins of notoginseng total saponins. Researchers first established a systematic chemical composition spectrum using high-performance liquid chromatography-mass spectrometry, covering the main components (such as notoginsenoside R1, ginsenoside Rg1, Re, Rb1, Rd, accounting for approximately 60–70% of the total saponins). It also covers a variety of trace saponins (such as ginsenoside Rh4, Rg6 and notoginsenoside T5) [235]. Then, by using new methods such as “content-weighted component-target network” and “the adjusted efficacy score”, combined with animal experiments, It was determined that notoginsenoside R1, ginsenoside Rg1, Rb1, Rd and Re are bioequivalent components highly correlated with the overall efficacy, and were ultimately established as Q-markers of XST [236, 237]. Centering on these Q-markers, the key production processes (extraction and column chromatography) for preparing XST were identified and optimized. Meanwhile, a computational model was established to characterize the relationship between key process parameters and Q-marker levels, thereby achieving process prediction and quality improvement [238]. In addition, ginsenoside Rd has been found to be an active substance with potential allergenicity. Therefore, based on the QbD concept, corresponding internal control standards have been established to keep its content within a range that can avoid adverse reactions without reducing therapeutic efficacy [239, 240].

At present, the quality evaluation of TCM is mostly oriented towards the chemical components of efficacy or toxicity, but it lacks a direct connection with biological activity and is difficult to reflect clinical efficacy and safety. With the advancement of science and the guidance of the holistic concept of Traditional Chinese Medicine, there is an urgent need for a more systematic and comprehensive evaluation approach. Thus, the discovery strategy of Q-marker systems based on chemical activity gradually took shape, mainly relying on methods such as network pharmacology, activity equivalence assessment in systems biology, spectral-activity relationship, and PK-PD analysis [233]. Among them, the analysis based on PK-PD aims to establish qualitative and quantitative PK/PD correlations from different in vivo PK/PD processes. Through quantitative PK-PD correlations, the relationship between the key active constituents and key pharmacological effects of TCM on diseases can be discovered, such as the TCMIP strategy proposed by Liu et al. [72]. This typical example is the research on the Q-marker system of the Yuanhuzhitong plate (YZP). Firstly, the chemical composition of YZP was clarified through chemical fingerprinting and multi-component quantitative analysis [241]. Then, by integrating in vitro intestinal absorption experiments, serum drug chemistry and PK studies, the ADME process and PK characteristics were revealed, and potential key active substances were screened [242, 243]. Subsequently, a component-target network was constructed. The results showed that the basis of its action mainly involved targets (opioid receptors, dopamine receptors, cation channels, etc.), revealing that YZP has pharmacological effects such as analgesia, anti-anxiety, anti-depression and vasodilation [244]. On this basis, seven candidate Q-markers were screened out from aspects such as component abundance, source specificity, drug-like properties and active contribution. Further, by integrating grey relational analysis with the least square method, support vector machine, etc., the quantitative relationship between Q-markers and drug efficacy was precisely characterized. Eventually, three Q-markers were determined as the minimum combination for quality control [245]. This method verifies the contribution of the key active substances of YZP to the overall therapeutic effect, and clarifies the synergistic mechanism among multiple components, establishing a scientific, simple and promotable quality evaluation system for YZP.

**Fig. 6:**
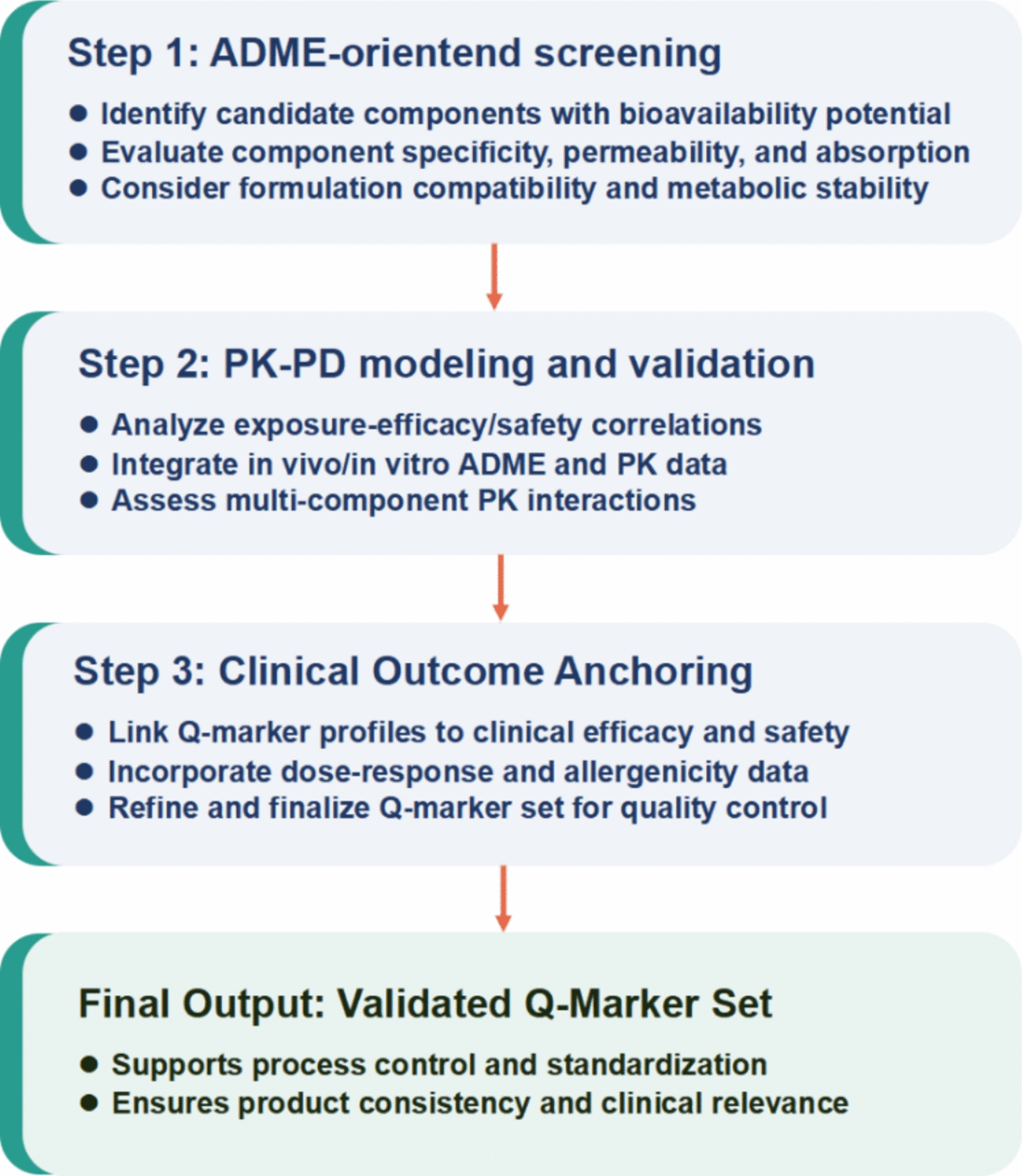
Unified Q-marker workflow integrating ADME-PK-clinical data

The efficacy and material basis of the same TCM in different formulas vary. Therefore, it is necessary to rely on the Traditional Chinese Medicine theory to predict Q-markers. This has led to the formation of the “Q-marker discovery strategy guided by TCM theory”, with prescription compatibility as the core, combined with network pharmacology, PK/PD and metabolomics, to analyze prescriptions and screen Q-markers that conform to Traditional Chinese Medicine theory [233]. For instance, Yuanhuzhitong dropping pills (YZDP) are a combination of *Corydalis Rhizoma* and *Angelicae Dahuricae Radix*. Zhang et al. [246] screened the Q-Marker of this drug based on the “property-response-component” ternary relationship. Firstly, YZDP was identified, and 51 prototype components and metabolites of YZDP absorbed in plasma and brain tissue were obtained. It was found that *Corydalis Rhizoma* and *Angelicae Dahuricae Radix* could act synergistically on multiple G protein receptors (GPCR). Then, through olfactory and gutaste bionic models, molecular docking and GPCR experiments, it was determined that tetrahydropalmatine and protopine were the basis for pungent and bitter flavors, and imperatorin was the basis for pungent flavors. The pharmacodynamic mechanism was verified through animal experiments, network pharmacology and metabolomics. In the multi-component PK study of YZDP, it was confirmed that there was a significant PK interaction after the combination of the two drugs (the C_max_ and AUC_0-∞_ of corydaline, tetrahydropalmatine and protopine were significantly increased; while the C_max_ of imperatorin and isoimperatorin decreased, however, the retention time is prolonged and the absorption is delayed). It was determined that corydaline, tetrahydropalmatine, protopine, imperatorin and isoimperatorin were the Q-markers of YZDP. The rationality of the formula compatibility was revealed and the basis for Q-Marker of the preparation was provided. To better illustrate the relationship between Q-markers, ADME properties, and clinical outcomes, we have summarized representative examples in Table S2(Additional File 1). Meanwhile, we have added a unified Q-Marker workflow(Fig. 6). Since this table is too lengthy, it has been included in the supplementary materials.

Bioavailability is essentially the transformation of “the presence of components” into “availability”. The multi-modal integrated quality evaluation method incorporates biological effects into quality control, enabling the quality evaluation of TCM to shift from merely focusing on component content to being linked with clinical efficacy, and achieving the integration of the entire industrial chain with the aid of Q-markers. This transformation conforms to the guidance of Traditional Chinese Medicine theory and the holistic concept, meets the core requirement of optimizing bioavailability through quality control, and is now connecting Traditional Chinese Medicine with modern medicine. However, we still need to overcome the following several challenges: (1) The active substances still need to be revealed in depth, and the Q-marker should be oriented towards biological effects; (2) The guidance for the discovery of Q-marker in the theory and material basis of Traditional Chinese Medicine needs to be strengthened; (3) It is necessary to clarify the dose–response relationship between the content of Q-marker and clinical efficacy/safety. Going forward, the integration of multi-modal analysis, QbD, PK-PD modeling, and Traditional Chinese Medicine theory will be essential for building a scientific, standardized, and internationally recognized TCM quality evaluation system.

## Conclusions and future perspectives

This review highlights the central role of bioavailability research in TCM as a crucial link between the in vivo behavior of complex compounds and their clinical efficacy and safety. Bioavailability and quality control form a dynamically interdependent system: the former reflects the clinical manifestation of quality, while the latter provides a foundation for optimizing therapeutic outcomes.

In recent years, considerable progress has been made in understanding the ADMET characteristics, PK, and bioavailability of TCM, laying the groundwork for improving clinical efficacy and managing potential risks. However, challenges remain particularly in distinguishing toxic from efficacious components, especially in inherently toxic TCM. Ensuring safety continues to be a bottleneck in the global acceptance of TCM, underscoring the need for stronger control over both exogenous and endogenous toxicants, such as pesticide residues and heavy metals. Although technologies like GC/LC–MS, sensor arrays, and AI algorithms offer promising capabilities, their application is still limited by the lack of standardized annotations and large-scale, high-quality datasets.

It is widely recognized that TCM molecules, due to their complex structures and multi-target mechanisms, differ fundamentally from Western drugs. They often exhibit low absorption and diverse interactions. Therefore, enhancing bioavailability requires integrated strategies including improving raw material quality, optimizing processing methods, developing nano-formulations, and designing targeted delivery systems. Theories such as molecular compatibility, integrated Traditional Chinese-Western Medicine, and the Q-marker system provide valuable guidance for enhancing efficacy and reducing toxicity, while supporting the development of effect-oriented quality control systems.

To establish a modern, process-oriented quality control framework, a multidisciplinary integration strategy is essential. Guided by the holistic concept of Traditional Chinese Medicine and the Jun-Chen-Zuo-Shi principle, key therapeutic functions should first be identified. Then, integrated technologies should be applied to analyze the biological behavior of components covering molecular-level data such as ADMET profiles, target-pathway networks, and bioavailability. These insights help reveal the pharmacological basis of TCM prescriptions. Through PK/PD modeling, quantitative correlations between clinical efficacy and in vivo behavior can be established, forming a “preclinical-clinical” efficacy map. The key active components identified through this process can then be traced back to guide Q-Marker selection, optimize raw materials, processing, and formulation design, and even support personalized treatment based on metabolomics or genetic characteristics. This bidirectional, dynamic framework, starting from the whole, analyzing at the molecular level, and feeding back into the whole, represents a practical path for quality control and bioavailability optimization in modern TCM.

Looking ahead, future research should focus on integrating toxicokinetic data and developing dynamic models to assess the balance between efficacy and toxicity. The standardization and intelligent analysis of omics data will be critical to achieving closed-loop management of quality, safety, and therapeutic outcomes. In addition, advances in personalized medicine, based on metabolic, genetic, or microbiota profiles, may drive the development of precision Traditional Chinese Medicine interventions. Ultimately, the modernization of TCM quality evaluation must remain grounded in Traditional Chinese Medicine theory and the holistic concept. Only through the integration of innovative technologies with foundational principles can we construct a scientific, effective, and internationally recognized system for quality control and bioavailability regulation in TCM.

## Supplementary Information

Additional file1

## Data Availability

No data was used for the research described in the article.

## Abbreviations

AC: Aconitine
ADME: Absorption, distribution, metabolism, excretion
ADMET: Absorption, distribution, metabolism, excretion, and toxicity
AFB1: Aflatoxin B1
AFB2: Aflatoxin B2
AFG1: Aflatoxin G1
AFG2: Aflatoxin G2
AFM1: Aflatoxin M1
AI: Artificial intelligence
ALI: Acute lung injury
ASDs: Amorphous solid dispersions
AUC: Area under the concentration–time curve
BBB: Blood–brain barrier
BBR-NE: Berberine into nanoemulsion
BCS: Biopharmaceutics classification system
BCSCC: Biopharmaceutics classification system of Chinese materia medica composition
BLNM: Baicalin nanomicelles
BZYXP: Baiziyangxin pills
BYD: Baoyuan deocction
CA-VO: *Chuanxiong Rhizoma* And *Angelicae Sinensis Radix* volatile oils
ChP: Chinese Pharmacopoeia
CIT: Citrinin
CL: Clearance
C_max_: Maximum plasma concentration
CNS: Central nervous system
CSAN: Chinese medicine self-assembly nanostrategy
CTD: Cantharidin
CYP450: Cytochrome P450
DL: Deep learning
DON: Deoxynivalenol
FB1: Fumonisin B1
FB3: Fumonisin B3
GC–MS: Gas Chromatography-Mass Spectrometry
GLUT: Glucose transporter
GST: Glutathione-S-transferases
GPCR: G protein receptors
HA: Hypaconitine
HHI: Herb-herb interactions
HDI: Herb-drug interactions
HRMS: High-resolution mass spectrometry
HSA: Human serum albumin
ICD: Immunogenic cell death
LBP: Lycium barbarum polysacharides
LC–MS: Liquid chromatography
LC–MS/MS: Liquid chromatography-tandem mass spectrometry
LMB: LipoMicel® Berberine
MA: Mesaconitine
ML: Machine learning
MRP: Multidrug resistance-associated transporter
NDDS: Nano drug delivery systems
OAT: Organic anion transporter
OCT: Organic cation transporter
PAs: Pyrrolizidine alkaloids
PAT: Patulin
Papp: Apparent permeability coefficient
PD: Pharmacodynamic
PEPT: Peptide transporter
PEG: Polyethylene glycol
P-gp: P-glycoprotein
PK: Pharmacokinetic
PLGA: Polylactic acid-glycolate
PRTS: *Raridis Phizoma* Total saponins
QbD: Quality by design
Q-marker: Quality Marker
QSAR/QSPR: Quantitative structure–activity relationship/quantitative structure–property relationship
QuEChERS: Quick, easy, cheap, effective, rugged, and safe
QZJFD: Qingzaojiufei decoction
RF: Random forest
SPE: Solid-phase extraction
SO_2_: Sulfur dioxide
SULT: Sulfotransferases
SAB: Salvianolic acid B
TA: Total alkaloids
TCM: Traditional Chinese medicines
T_max_: Shortens the time to peak
T_1/2_: Prolongs the half-life
TDC: Tyrosine decarboxylase
UGT: UDP-glucuronosyltransferases
XST: Xuesaitong injection
YZP: Yuanhuzhitong plate
YZDP: Yuanhuzhitong dropping pills
ZEN: Zearalenone
