# Supplementary material for: Holistic concept guided quality control of traditional Chinese medicines for optimizing bioavailability
Source: Chin Med. 2025 Nov 25;20:202. doi: 10.1186/s13020-025-01269-w (PMC12645736; doi:10.1186/s13020-025-01269-w)
Supplement: Supplementary file 1 — Additional file1 [file 13020_2025_1269_MOESM1_ESM.docx]

Table S1 Statistics on the classification of toxic tradional Chinese medicines(TCMs)in the 2025 ChP.

| Toxicity level | Latin name | Active substances | Toxic substances | PK parameters | Animal model | Toxicology | Refs |
| --- | --- | --- | --- | --- | --- | --- | --- |
| Slightly toxic | *Erycibes Caulis* | Scopoletin | Scopoletin | **Scopoletin 10 mg/kg:**  AUC_0-t_=61.984±9.406μg/L/h  AUC_0-∞_=69.910±16.189μg/L/h  MRT_0-t_=0.685±0.166h  MRT_0-∞_=0.948±0.334h  T_1/2=_0.656±0.379h  T_max_=0.331±0.118h  CL=148.364±29.053L/h/kg  C_max_=101.314±52.201μg/ml | p.o.  Male SD rats | LD_50_>2000mg/kg | [1]  [2]  [3] |
|  | *Murrayae Folium et Cacumen* | Alkaloid,  Flavonoids,  Coumarin | Unknown | - | - | - | [4] |
|  | *Eupolyphaga Steleophaga* | Proteins,  Active peptide,  Amino acid,  Polysaccharide | Unknown | - | - | - | [5] |
|  | *Gleditsiae SINENSIS Fructus* | Triterpenoid saponins,  Polysaccharides,  Cellulose | Unknown | - | - | - | [6] |
|  | *Euphorbiae Hirtae Herba* | Intermedine,  Lycopsamine,  Lycopsamine N-oxide | Intermedine,  Lycopsamine,  Lycopsamine N-oxide | - | - | Hepatotoxicity | [7] |

Continue Table S1

| Toxicity level | Latin name | Active substances | Toxic substances | PK parameters | Animal model | Toxicology | Refs |
| --- | --- | --- | --- | --- | --- | --- | --- |
| Slightly toxic | *Toosendan Fructus* | Toosendanin | Toosendanin | **Toosendanin 60g/kg:** AUC=109.5±39.7  MRT=3.16±0.75  T_1/2=_3.45±1.56  T_max_=0.39±0.17  CL=6.25±231 | i.g.  Male SD rats | - | [8]  [9]  [10] |
|  | *Sinopodophylli Fructus* | Podophyllotoxin | Podophyllotoxin | **Podophyllotoxin:** AUC=63.58±8.48µg/mL/min  T_1/2α=_0.88±0.06h  T_1/2β=_29.93±4.72h  T_max_=1.23±0.09h  CL=0.16±0.02mg/h/mg  C_max_=3.87±0.67μg/ml | i.p.  Female male, Kunming mice | LD_50_=45.83mg/kg | [11]  [12]  [13] |
|  | *Hirudo* | Hirudin | Aflatoxin B1 | - | - | - | [14] |
|  | *Menispermi Rhizoma* | Sinomenine | Sinomenine | **Sinomenine 40 mg/kg:**  AUC=1053.80±427.69h∙μg/L  MRT=5.15±2.47h  T_max_=1.65±1.48h  CL=30.37±11.85L/h/kg | i.p.  Male SD rats | LD_50_=453.54mg/kg | [15]  [16] |
|  | *Armeniacae Semen Amarum* | Amygdalin | Hydrocyanic acid | - | Male, SD rats | LD_50_=880mg/kg | [17] |

Continue Table S1

| Toxicity level | Latin name | Active substances | Toxic substances | PK parameters | Animal model | Toxicology | Refs |
| --- | --- | --- | --- | --- | --- | --- | --- |
| Slightly toxic | *Artemisiae Argyi Folium* | Camphor,  Caryophyllene oxide | Camphor,  Caryophyllene oxide | **Camphor 25mg/kg:**  T_1/2_=0.13±0.01h  T_max_=0.03±0.02h  C_max_=8317±517µg/L  MRT_0-∞_=0.15±0.01h  AUC_0-t_=1583±120µg/L/h  CL=15.85±1.21L/h/kg  **Caryophyllene oxide 25mg/kg:**  T_1/2_=1.21±0.69h  Tmax=0.03±0.02h  Cmax=4244±488µg/L  MRT_0-∞_=1.06±0.48h  AUC_0-t_=1358±70.1µg/L/h  CL=17.22±1.33L/h/kg | i.v.  Male, SD rats | - | [18][19] |
|  | *Illicii Cortex* | Myristicin | Safrol | - | - | LC_50_=15.26µg/mL  LC_50_=39.45µg/mL  LC_50_=72.18µg/mL | [20] |
|  | *Knoxiae Radix* | 3-hydroxy-morindone,  Lucidine | Anthraquinones | - | - | - | [21] |
|  | *Zanthoxyli Radix* | Nitidine chloride | Nitidine chloride | **Nitidine chloride 25mg/kg:**  C_max_=96.48±5.30ng/mL  T_max_=2h | i.g.  Male, SD rats | Hepatotoxicity,  Cardiotoxicity,  Nephrotoxicity | [22]  [23] |

Continue Table S1

| Toxicity level | Latin name | Active substances | Toxic substances | PK parameters | Animal model | Toxicology | Refs |
| --- | --- | --- | --- | --- | --- | --- | --- |
| Slightly toxic | *Euodiae Fructus* | Evodiamine | Evodiamine | **Evodiamine 1mg/kg:**  AUC=9.13μg·min/mL  T_1/2=_74.9min  C_max_=0.34μg/mL | i.v.  Male SD rats | LD_50_=77.7938mg/kg | [24] |
|  | *Picrasmae Ramulus et Folium* | 4,5-Dimethoxycanthin-6-one | 4,5-Dimethoxycanthin-6-one | **4,5-Dimethoxycanthin-6-one 0.8mL/kg:**  AUC=124.27±48.08ng/mL/min  T_1/2α=_1.15±0.52min  T_1/2β=_1.15±0.52min  CL=450.64±145.25mL/min | i.v.  Mongrel | - | [25] |
|  | *Psammosilenes Radix* | Triterpenoid saponins,  Cyclic peptides,  Carboline alkaloids | Unknown | - | - | - | [26] |
|  | *Carotae Fructus* | Volatile oils,  Sesquiterpenes,  Flavonoids | Volatile oils | - | - | - | [27] |
|  | *Impatientis Semen* | Balsaminasterol,  Fatty oil | Unknown | - | - | - | [28] |
|  | *Cnidii Fructus* | Osthole | Unknown- | - | - | - | [29] |
|  | *Gleditsiae Fructus Abnormalis* | Triterpenoid saponins,  Volatile oils | Olefin benzene | - | - | - | [30][31] |

Continue Table S1

| Toxicity level | Latin name | Active substances | Toxic substances | PK parameters | Animal model | Toxicology | Refs |
| --- | --- | --- | --- | --- | --- | --- | --- |
| Slightly toxic | *Aconiti Kusnezoffii Folium* | Aconitine,  Mesaconine,  Hypaconitine | Aconitine,  Mesaconine,  Hypaconitine | **Aconitine 0.5g/kg:**  AUC=13.47±3.28µg/L/h  T_max_=4.38±3.11h  C_max_=1.28±0.34μg/L  CL=4.62±01.29L/h/kg  **Mesaconine 0.5g/kg**  AUC=26.39±5.42µg/L/h  T_max_=2.18±1.40h  C_max_=2.66±0.50μg/L  CL=9.89±1.93L/h/kg  **Hypaconitine 0.5g/kg:**  AUC=62.90±26.89µg/L/h  T_max_=2.14±0.90h  C_max_=6.37±1.96μg/L  CL=4.02±1.80L/h/kg | p.o.  Male SD rats | - | [32] |
|  | *Carpesii Fructus* | Carabrone | Volatile oils | - | - | - | [33] |
|  | *Dryopteridis Crassirhizomatis Rhizoma* | Filixic acid ABA,  Albaspidin AP,  Albaspidin BB | Unknown | - | - | - | [34] |
|  | *Dryopteridis Crassirhizomatis Rhizoma Carbonisatum* | Filixic acid ABA,  Albaspidin AP | Unknown | - | - | - | [35] |
|  | *Osmundae Rhizoma* | OsMundacetone | Unknown | - | - | - | [36] |

Continue Table S1

| Toxicity level | Latin name | Active substances | Toxic substances | PK parameters | Animal model | Toxicology | Refs |
| --- | --- | --- | --- | --- | --- | --- | --- |
| Slightly toxic | *Paridis Rhizoma* | Polyphyllin II,  Polyphyllin VII | Polyphyllin II,  Polyphyllin VII | **Polyphyllin II 1 mg/kg:**  AUC_0-t_=4032.45±781.55µg/L/h  MRT_0-t_=14.88±1.08h  T_1/2z_=147.08±113.20h  T_max_=0.17±0.00h  C_max_=718.21±128.87µg/L  CL_z_=0.10±0.09L/h/kg  **Polyphyllin VII 1 mg/kg:**  AUC_0-t_=4459.23±109.11µg/L/h  MRT_0-t_=5.15±0.88h  T_1/2z_= 49.43±37.43h  T_max_=0.17±0.00h  C_max_=398.28±70.86µg/L  CL_z_=1.55±0.25L/h/kg | i.v.  Male SD rats | - | [37] |
|  | *Tribuli Fructus* | Diosgenin,  Dioscin,  Tribuloside | Diosgenin,  Dioscin,  Tribuloside | - | Rats, mouse | Diosgenin: p.o. LD_50_>8g/kg  Dioscin: s.c. LD_50_>300mg/kg | [38] |
|  | *Entadae Semen* | Phaseoloidin,  Entadamide A | Unknown | - | - | - | [39] |
|  | *Pterocephali Herba* | Iri doid glycosides,  Triterpenoids | Unknown | - | - | - | [40] |

Continue Table S1

| Toxicity level | Latin name | Active substances | Toxic substances | PK parameters | Animal model | Toxicology | Refs |
| --- | --- | --- | --- | --- | --- | --- | --- |
| Slightly toxic | *Bruceae Fructus* | Bruceine D,  Bruceine E | Bruceine D,  Bruceine E | **Bruceine D 2.05mg/kg:**  AUC_0-t_=0.04±0.01mg·h/L  MRT_0-∞_=2.28±1.53h  C_max_=0.05±0.03mg/L  T_max_=0.54±0.34h  T_1/2_=1.54±1.37h  F=5.0%  **Bruceine E 9.1mg/kg:**  AUC_0-t_=0.34±0.14mg·h/L  MRT_0-∞_=3.68±1.67h  C_max_=0.19±0.11mg/L  T_max_=0.66±0.30h  T_1/2_=2.26±1.29h  F=5.9% | p.o.  Male SD rats | - | [41] |
| Toxic | *Toxicodendri Resina* | Urushiols | Urushiols | - | - | - | [42] |
|  | *Berberidis Radix* | Berberine hydrochloride,  Jatrorrhizine,  Palmatine chloride | Berberine hydrochloride | - | - | - | [43] |
|  | *Pseudolaricis Cortex* | Pseudolaric acid A,  Pseudolaric acid B | Unknown | - | - | - | [44] |
|  | *Momordicae Semen* | Momordica saponinI,  Momordica saponinⅡ | Momordica saponinI,  Momordica saponinⅡ | - | - | - | [45] |

Continue Table S1

| Toxicity level | Latin name | Active substances | Toxic substances | PK parameters | Animal model | Toxicology | Refs |
| --- | --- | --- | --- | --- | --- | --- | --- |
| Toxic | *Sophorae Tonkinensis Radix et Rhizoma* | Matrine,  (+)-Oxymatrine | Matrine,  (+)-Oxymatrine | **Matrine 30mg/kg:**  AUC=100.35mg·min/L  C_max_=21.11mg/L  T_1/2α_=80.4min  T_1/2β_=3.51h  T_max_=45min  **(+)-Oxymatrine:**  T_1/2_=4.181h  C_max_=605.5ng/mL  T_max_=0.75h | Matrine: i.m. SD rats,  (+)-Oxymatrine: SD rats, Caco-2 model | - | [46] |
|  | *Arisaematis Rhizoma* | Apigenin,  Trigonelline | Lectins,  Calcium oxalate raphides | - | - | - | [47] |
|  | *Arisaematis Rhizoma Preparatum* | Apigenin | Lectins,  Calcium oxalate raphides | - | - | - | [48] |
|  | *Kansui Radix* | Kansuinine A,  Kansuinine B,  Euphol | Ingenane diterpene | **Euphol 30min:**  SGF=39.84±2.34µM  SIF=77.33±2.34µM  T_1/2_=4.52±1.43h | Simulated gastrie fluid, Simulated intestinal fluid | - | [49][50] |
|  | *Laggerae Herba* | Pterodontic acid,  Absinthin | Unknown | - | - | - | [51] |

Continue Table S1

| Toxicity level | Latin name | Active substances | Toxic substances | PK parameters | Animal model | Toxicology | Refs |
| --- | --- | --- | --- | --- | --- | --- | --- |
| Toxic | *Euphorbiae Semen Pulveratum* | Euphorbia factor L1,  Euphorbia factor L2,  Euphorbia factor L3 | Euphorbia factor L1,  Euphorbia factor L2,  Euphorbia factor L3 | **Euphorbia factor L1 30g/kg:**  T_1/2_=14.048±1.252h  Ke=0.050±0.005h^-1^  **Euphorbia factor L2 30g/kg:**  T_1/2_=12.728±1.197h  Ke=0.055±0.005h^-1^  **Euphorbia factor L3 30g/kg:**  T_1/2_=13.044±1.43h  Ke=0.054±0.006h^-1^ | i.g.  SD rats | - | [52] |
|  | *Euphorbiae Semen* | Euphorbia factor L1,  Euphorbia factor L2,  Euphorbia factor L3 | Euphorbia factor L1,  Euphorbia factor L2,  Euphorbia factor L3 | **Euphorbia factor L1 9.5mg/kg:**  AUC_0-t_=100.9 ± 46.0µg·h/L  AUC_0-∞_=158.0 ± 82.4µg·h/L  T_1/2_=15.9 ± 3.3h  T_max_=1.1 ± 0.5h  C_max_=30.4 ± 12.0µg/L  **Euphorbia factor L2 6mg/kg:**  AUC_0-t_=830.0±113.0µg·h/L  AUC_0-∞_=2668.0±336.0µg·h/L  T_1/2_=4.2±1.8h  T_max_=6.2±2.0h  C_max_=81.3±35.5µg/L | i.g.  SD rats | LD_50_> 100 μg/ear | [53] |

Continue Table S1

| Toxicity level | Latin name | Active substances | Toxic substances | PK parameters | Animal model | Toxicology | Refs |
| --- | --- | --- | --- | --- | --- | --- | --- |
| Toxic | *Euphorbiae Semen* | Euphorbia factor L1,  Euphorbia factor L2,  Euphorbia factor L3 | Euphorbia factor L1,  Euphorbia factor L2,  Euphorbia factor L3 | **Euphorbia factor L3 10.5mg/kg:**  AUC_0-t_=680.6±230.1µg·h/L  AUC_0-∞_=765.9±172.8µg·h/L  T_1/2_=10.7±3.3h  T_max_=3.2±0.8h  C_max_=73.6±22.7µg/L | i.g.  SD rats | LD_50_> 100 μg/ear | [53] |
|  | *Genkwa Flos* | Genkwanin,  Yuanhuacine,  Yuanhuadine | Yuanhuacine,  Yuanhuadine | **Genkwanin 0.3g/kg:**  C_max_=449.21±57.74ng/mL  T_max_=0.39±0.14h  T_1/2_=3.96±2.91h  AUC_0-t_=500.98±141.25ng/mL·h  AUC_0-∞_=507.63±139.00h  MRT_0-t_=1.29±0.19h  **Yuanhuacine 0.3g/kg:**  C_max_=198.50±27.76ng/mL  T_max_=0.61±0.14h  T_1/2_=3.47±2.27h  AUC_0-t_=348.82±74.94ng/mL·h  AUC_0-∞_=356.26±81.23h  MRT_0-t_=2.23±0.59h | i.g.  Male, SD rats | - | [54] |

Continue Table S1

| Toxicity level | Latin name | Active substances | Toxic substances | PK parameters | Animal model | Toxicology | Refs |
| --- | --- | --- | --- | --- | --- | --- | --- |
| Toxic | *Curculiginis Rhizoma* | Curculigoside A,  Curculigoside B,  Curculigoside C | Unknown | **Curculigoside A 100mg/kg:**  T_1/2_=32.7±13.5h  T_max_=0.33±0.12h  CL=75.0±16.0L/h/kg  V_d_=3674.4±1921.7L/kg  C_max_=128.2±46.2ng/mL  AUC_0-t_=1241.4±280.6ng/mL/h  AUC_0-∞_=1374.0±244.0ng/mL/h  MRT_0-t_=26.29±2.14h  MRT_0-∞_=39.09±10.34h  **Curculigoside C 15mg/kg:**  T_1/2_=2.022±0.184h  T_max_=0.106±0.149h  AUC_0-60_=62.731±10.149µg/L/h  AUC_0-∞_=66.310±10.563µg/L/h  F=2.01%  V_d_=673.157±116.509L/kg  CL_z_=231.036±36.69L/h/kg | p.o.  Female SD rats, Wistar rats | - | [55]  [56] |
|  | *Calomelas* | Hg_2_Cl_2_ | Hg | **Tianwangbuxin pills(bioaccessible):** SGF=0.59µg/g; SIF=0.63µg/g;  Bioaccessibility=0.016%; MDE=22.0µg/day | Simulated gastrie fluid, Simulated intestinal fluid | - | [57][58] |

Continue Table S1

| Toxicity level | Latin name | Active substances | Toxic substances | PK parameters | Animal model | Toxicology | Refs |
| --- | --- | --- | --- | --- | --- | --- | --- |
| Toxic | *Chelidonii Herba* | Chelerythrine,  Chelidonine,  Sanguinarine | Chelerythrine | **Chelerythrine 0.1mg/kg：**  C_max_=69.79±15.41ng/mL  T_max_=0.42±0.13h  **Chelerythrine 0.1mg/kg：**  C_max_=5.04±1.00ng/mL  T_max_=1.83±0.26h | Chelerythrine i.m.  Chelerythrine p.o.  (Landrace × Large White) female pigs | - | [59] |
|  | *Ginkgo Semen* | Ginkgolide,  Bilobalide | Ginkgotoxin,  Ginkgolic acid | **Ginkgolic acid(13:0) 100mg/kg:**  AUC_0-t_=930.52±96.21ng/L/h  AUC_0-∞_=957.91±93.86ng/L/h  MRT_0-t_=4.66±0.91h  MRT_0-∞_=5.24±1.15h  T_1/2_=3.21±1.65h  T_max_=0.50±0.01h  C_max_=252.94±44.11ng/mL  **Ginkgolic acid(15:1) 100mg/kg:**  AUC_0-t_=2149.40±315.50ng/L/h  AUC_0-∞_=2181.11±307.83ng/L/h  MRT_0-t_=4.71±1.07h  MRT_0-∞_=4.95±1.06h  T_1/2_=2.59±1.00h  T_max_=0.58±0.20h | p.o.  Male SD rats | Cytotoxicity | [60] |

Continue Table S1

| Toxicity level | Latin name | Active substances | Toxic substances | PK parameters | Animal model | Toxicology | Refs |
| --- | --- | --- | --- | --- | --- | --- | --- |
| Toxic | *Physochlainae Radix* | Scopolamine,  Anisodamine,  Hyoscyamine | Scopolamine,  Anisodamine,  Hyoscyamine | **Scopolamine 0.62g/kg:**  T_max_=0.08±0.00h  C_max_=45.60±9.04ng/mL  T_1/2_=2.99±0.79h  AUC_0-t_=30.36±13.00h·ng/mL  AUC_0-∞_=30.37±13.31h·ng/mL  CL=23.48±6.37L/h/kg  **Anisodamine 0.62g/kg:**  T_max_=0.75±0.27h  C_max_=142.35±43.30ng/mL  T_1/2_=3.15±1.68h  AUC_0-t_=570.90±272.72h·ng/mL  AUC_0-∞_=571.90±272.04h·ng/mL  CL=1.36±0.52L/h/kg  **Hyoscyamine 0.62g/kg:**  T_max_=0.88±0.14h  C_max_=124.86±30.02ng/mL  T_1/2_=2.08±0.21h  AUC_0-t_=409.23±254.71h·ng/mL  AUC_0-∞_=409.24±254.71h·ng/mL  CL=1.88±0.48L/h/kg | p.o.  Male SD rats | - | [61] |

Continue Table S1

| Toxicity level | Latin name | Active substances | Toxic substances | PK parameters | Animal model | Toxicology | Refs |
| --- | --- | --- | --- | --- | --- | --- | --- |
| Toxic | *Aconiti Radix Cocta* | Benzoylaconine,  Benzoylhypacoitine,  Benzoylmesaconine | Aconitine,  Mesaconine,  Hypaconitine | **Aconitine 0.5g/kg:**  AUC=13.47±3.28µg/L/h  T_max_=4.38±3.11h  C_max_=1.28±0.34μg/L；  CL=4.62±01.29L/h/kg  **Mesaconine 0.5g/kg:**  AUC=26.39±5.42µg/L/h  T_max_=2.18±1.40h  C_max_=2.66±0.50μg/L  CL=9.89±1.93L/h/kg  **Hypaconitine 0.5g/kg:**  AUC=62.90±26.89µg/L·h  T_max_=2.14±0.90h  C_max_=6.37±1.96μg/L  CL=4.02±1.80L/h/kg | p.o.  Male SD rats | - | [32] |
|  | *Typhonii Rhizoma* | Thymine | Calcium oxalate raphides | - | - | - | [62] |
|  | *Bungarus Parvus* | BM-Apotxin | Snake venom | - | - | - | [63] |
|  | *Pinelliae Rhizoma* | Alkaloids,  Volatile oils | Lectins,  Calcium oxalate raphides | - | - | - | [64] |

Continue Table S1

| Toxicity level | Latin name | Active substances | Toxic substances | PK parameters | Animal model | Toxicology | Refs |
| --- | --- | --- | --- | --- | --- | --- | --- |
| Toxic | *Xanthii Fructus* | Xanthatin,  Neochlorogenic acid,  Cryptochlorogenic acid | Atractyloside | **Xanthatin 2.4mg/200g:**  T_1/2_=108.58±32.82min  C_max_=418.72±137.51ng/mL  AUC_0–t_=14,340.20±7122.41ng·h/mL  AUC_0–∞_=15,538.97±7733.12ng·h/mL  CL=0.13±0.14mL/min  V_d_=46.85±20.19mL  **Neochlorogenic acid:**  T_max_=3.75±0.46h  T_1/2_=1.70±0.61h  **cryptochlorogenic acid:**  T_max_=2.75±0.27h  T_1/2_=2.12±0.68h  **Atractyloside 11.4mg/kg:**  T_1/2_=13.64h  C_max_=41.98µg/mL  AUC_0–t_=132.70µg·h/L  T_max_=0.38h | Xanthatin i.v.  Neochlorogenic acid i.g.  cryptochlorogenic acid i.g.  Atractyloside i.v.  SD rats | - | [65] |
|  | *Anemones Raddeanae Rhizoma* | Raddeanin A | Triterpenoid saponins | - | - | - | [66] |
|  | *Scorpio* | Active peptide | Scorpion venum | - | - |  | [67] |

Continue Table S1

| Toxicity level | Latin name | Active substances | Toxic substances | PK parameters | Animal model | Toxicology | Refs |
| --- | --- | --- | --- | --- | --- | --- | --- |
| Toxic | *Aconiti Lateralis Radix Praeparata* | Benzoylaconine,  Benzoylhypacoitine,  Benzoylmesaconine | Aconitine,  Mesaconine,  Hypaconitine | **Aconitine 0.5g/kg:**  AUC=13.47±3.28µg/L/h  T_max_=4.38±3.11h  C_max_=1.28±0.34μg/L；  CL=4.62±01.29L/h/kg  **Mesaconine 0.5g/kg:**  AUC=26.39±5.42µg/L/h  T_max_=2.18±1.40h  C_max_=2.66±0.50μg/L  CL=9.89±1.93L/h/kg  **Hypaconitine 0.5g/kg:**  AUC=62.90±26.89µg/L/h  T_max_=2.14±0.90h  C_max_=6.37±1.96μg/L  CL=4.02±1.80L/h/kg | p.o.  Male SD rats | - | [32] |
|  | *Cinnabaris* | HgS | Hg | **Tianwangbuxin pills (bioaccessible):**  SGF=0.59µg/g; SIF=0.63µg/g;  Bioaccessibility=0.016%; MDE=22.0µg/day | Simulated gastrie fluid,  Simulated intestinal fluid | - | [57] |
|  | *Sulfur* | S | SO_2_ | - | - | - | [68] |

Continue Table S1

| Toxicity level | Latin name | Active substances | Toxic substances | PK parameters | Animal model | Toxicology | Refs |
| --- | --- | --- | --- | --- | --- | --- | --- |
| Toxic | *Meliae Cortex* | Toosendanin | Toosendanin | **Toosendanin 60g/kg:**  AUC=109.5±39.7  MRT=3.16±0.75  T_1/2=_3.45±1.56  T_max_=0.39±0.17  CL=6.25±231  C_max_=41.9±13.9 | i.g.  Male SD rats | - | [9]  [10]  [69] |
|  | *Euphorbiae Ebracteolatae Radix* | Triterpenoid | Unknown | - | - | - | [70] |
|  | *Bufonis Venenum* | Bufalin,  Resibufogenin | Bufalin,  Resibufogenin | **Bufalin 200mg/kg**  T_1/2_=4.537±2.442h  C_max_=466.5±116.920ng/mL  T_max_=0.938±0.125h  AUC_0-t_=1487.316±407.361ng/mL/h  MRT_0-t_=3.338±1.011h  **Resibufogenin 200mg/kg:**  T_1/2_=4.884±2.116h  C_max_=13.35±2.243ng/mL  T_max_=1.438±0.657h  AUC_0-t_=104.246±20.661ng/mL/h  MRT_0-t_=6.478±1.035h | i.g.  Male Wistar rats | - | [71] |
|  | *Pharbitidis Semen* | Resin glycosides | Resin glycosides | - | - | - | [72] |

Continue Table S1

| Toxicity level | Latin name | Active substances | Toxic substances | PK parameters | Animal model | Toxicology | Refs |
| --- | --- | --- | --- | --- | --- | --- | --- |
| Toxic | *Aconiti Kusnezoffii Radix Cocta* | Benzoylaconine,  Benzoylhypacoitine,  Benzoylmesaconine | Aconitine,  Mesaconine,  Hypaconitine | **Aconitine 0.5g/kg:**  AUC=13.47±3.28µg/L/h  T_max_=4.38±3.11h  C_max_=1.28±0.34μg/L；  CL=4.62±01.29L/h/kg  **Mesaconine 0.5g/kg:**  AUC=26.39±5.42µg/L/h  T_max_=2.18±1.40h  C_max_=2.66±0.50μg/L  CL=9.89±1.93L/h/kg  **Hypaconitine 0.5g/kg:**  AUC=62.90±26.89µg/L/h  T_max_=2.14±0.90h  C_max_=6.37±1.96μg/L  CL=4.02±1.80L/h/kg | p.o.  Male SD rats | - | [32] |
|  | *Euphorbiae Pekinensis Radix* | Euphol | Tirucallol | **Euphol 30min:**  SGF=39.84±2.34µM  SIF=77.33±2.34µM  T_1/2_=4.52±1.43h | Simulated gastrie fluid, Simulated intestinal fluid | - | [50][73] |
|  | *Phytolaccae Radix* | EsculentosideA | Esculentoside A | **Esculentoside A 5mg/kg:**  C_max_=46.70±23.98µg/L  AUC_0-t_=130.18±31.76h·ng/mL | i.g.  Male SD rats | - | [74] |

Continue Table S1

| Toxicity level | Latin name | Active substances | Toxic substances | PK parameters | Animal model | Toxicology | Refs |
| --- | --- | --- | --- | --- | --- | --- | --- |
| Toxic | *Dichroae Radix* | Febrifugine,  Isofebrifugine | Febrifugine | **Febrifugine i.v. 2mg/kg:**  C_max_=714.5±113.4ng/mL  T_1/2_=3.2±1.6h  AUC_0-t_=1607.5±334.1h·ng/mL  AUC_0-∞_=1661.5±349.21h·ng/mL  CL=1.2±0.2L/h/kg  MRT_0-t_=2.2±0.2h  MRT_0-∞_=2.8±0.3h  V_z_=5.6±3.0L/kg  **Febrifugine p.o. 6mg/kg:**  C_max_=704.7±126.5ng/mL  T_1/2_=2.6±0.5h  AUC_0-t_=2208.6±253.1h·ng/mL  AUC_0-∞_=2308.2±283.0h·ng/mL  CL=2.6±0.3L/h/kg  MRT_0-t_=3.5±0.2h  MRT_0-∞_=4.2±0.3h  V_z_=9.7±1.6L/kg | i.v. p.o.  SD rats | - | [75] |
|  | *Periplocae Cortex* | Periplocymarin,  Periplocoside M,  Periplocoside N | Periplocymarin,  Periplocoside M,  Periplocoside N | **Periplocymarin 12g/kg Male:**  C_max_=75.2±33.0ng/mL  T_max_=5.0±0.0h  T_1/2_=11.8±7.8h | i.g.  Male and female SD rats | - | [76] |

Continue Table S1

| Toxicity level | Latin name | Active substances | Toxic substances | PK parameters | Animal model | Toxicology | Refs |
| --- | --- | --- | --- | --- | --- | --- | --- |
| Toxic | *Periplocae Cortex* | Periplocymarin,  Periplocoside M,  Periplocoside N | Periplocymarin,  Periplocoside M,  Periplocoside N | **Periplocymarin 12g/kg Male:**  AUC_0-56h_=920.5±414.6µg·h/L  AUC_0-∞_=982.9±364.3µg·h/L  MRT=22.9±11.9h  **Periplocymarin 12g/kg Female:**  C_max_=152.1±47.9ng/mL  T_max_=7.5±1.9h, T_1/2_=8.8±3.1h  AUC_0-56h_=1340.1±303.7µg·h/L  AUC_0-∞_=1354.2±306.6µg·h/L  MRT=10.7±1.7h  **Periplocoside M 12g/kg Male:**  C_max_=38.5±12.7ng/mL  T_max_=0.7±0.5h  T_1/2_=20.3±7.8h  AUC_0-56h_=169.5±30.8µg·h/L  AUC_0-∞_=218.6±45.5µg·h/L  MRT=20.5±7.5h  **Periplocoside M 12g/kg Female:**  C_max_=194.5±41.1ng/mL  T_max_=1.8±1.4h  T_1/2_=26.8±8.0h  AUC_0-56h_=1865.5±364.9µg·h/L  AUC_0-∞_=2220.9±252.8µg·h/L | i.g.  Male and female SD rats | - | [76] |

Continue Table S1

| Toxicity level | Latin name | Active substances | Toxic substances | PK parameters | Animal model | Toxicology | Refs |
| --- | --- | --- | --- | --- | --- | --- | --- |
| Toxic | *Periplocae Cortex* | Periplocymarin,  Periplocoside M,  Periplocoside N | Periplocymarin,  Periplocoside M,  Periplocoside N | **Periplocoside M 12g/kg Female:**  MRT=29.4±8.3h  **Periplocoside N 12g/kg Male:**  C_max_=9.0±0.9ng/mL  T_max_=17.0±2.0h  T_1/2_=82.2±15.7h  AUC_0-56h_=372.9±42.7µg·h/L  AUC_0-∞_=1060.1±282.1µg·h/L  MRT=122.0±24.7h  **Periplocoside N 12g/kg Female:**  C_max_=9.0±2.1ng/mL  T_max_=23.0±2.0h  T_1/2_=189.8±141.7h  AUC_0-56h_=345.2±86.8µg·h/L  AUC_0-∞_=1993.0±1018.4µg·h/L  MRT=284.2±201.6h | i.g.  Male and female SD rats | - | [76] |
|  | *Daturae Flos* | Scopolamine,  Hyoscyamine | Scopolamine,  Hyoscyamine | **Scopolamine 0.62g/kg:**  T_max_=0.08±0.00h  C_max_=45.60±9.04ng/mL  T_1/2_=2.99±0.79h  AUC_0-t_=30.36±13.00h·ng/mL  AUC_0-∞_=30.37±13.31h·ng/mL  CL=23.48±6.37L/h/kg | p.o.  Male SD rats | - | [61][77] |

Continue Table S1

| Toxicity level | Latin name | Active substances | Toxic substances | PK parameters | Animal model | Toxicology | Refs |
| --- | --- | --- | --- | --- | --- | --- | --- |
| Toxic | *Realgar* | As_4_S_4_ | As | ***Realgar* 3mg/kg:**  MRT_0-1440_=190.50min  T_max_=100min  C_max_=19.50µg/L  AUC=2942.86µg/h/L  T_1/2_=478.51min  ***Realgar* 30mg/kg:**  MRT_0-1440_=257.82min  T_max_=120min  C_max_=25.21µg/L  AUC_0-t_=7380.95µg/h/L  T_1/2_=1453.42min | i.g. 28days  Beagle dog | - | [78] |
|  | *Ricini Semen* | Ricinine,  Ricin,  Castor oil | Ricinine,  Ricin,  Castor oil | **Ricinine 3.0mg/kg:**  C_max_=163±65µg/L  T_max_=0.7±0.4h  AUC_0-∞_=938±535ng/mL/h  AUC_0-tn_=760±406ng/mL/h  MRT_0-∞_=10.7±5.9h  MRT_0-t_=8.2±4.3h  T_1/2_=2.2±1.6h | i.g.  Male SD rats | Nephrotoxicity  Hepatotoxicity  Neurotoxicity | [79] |
|  | *Papaveris Pericarpium* | Morphine | Morphine | - | - | - | [80] |

Continue Table S1

| Toxicity level | Latin name | Active substances | Toxic substances | PK parameters | Animal model | Toxicology | Refs |
| --- | --- | --- | --- | --- | --- | --- | --- |
| Toxic | *Agkistrodon* | Snake venom metalloproteinases,  Snake venom serine proteases | Snake venom metalloproteinases,  Snake venom serine proteases | - | - | - | [81] |
|  | *Scolopendra* | Protein,  Polypeptides,  Polysaccharides | Protein | - | - | - | [82] |
| Highly toxic | *Aconiti Radix* | Aconitine,  Mesaconine,  Hypaconitine | Aconitine,  Mesaconine,  Hypaconitine | **Aconitine 0.5g/kg:**  AUC=13.47±3.28µg/L/h  T_max_=4.38±3.11h  C_max_=1.28±0.34μg/L  CL=4.62±01.29L/h/kg  **Mesaconine 0.5g/kg:**  AUC=26.39±5.42µg/L/h  T_max_=2.18±1.40h  C_max_=2.66±0.50μg/L  CL=9.89±1.93L/h/kg  **Hypaconitine 0.5g/kg:**  AUC=62.90±26.89µg/L/h  T_max_=2.14±0.90h  C_max_=6.37±1.96μg/L  CL=4.02±1.80L/h/kg | p.o.  Male SD rats | - | [32] |

Continue Table S1

| Toxicity level | Latin name | Active substances | Toxic substances | PK parameters | Animal model | Toxicology | Refs |
| --- | --- | --- | --- | --- | --- | --- | --- |
| Highly toxic | *Hyoscyami Semen* | Hyoscyamine,  Scopolamine | Hyoscyamine,  Scopolamine | **Scopolamine 0.62g/kg:**  T_max_=0.08±0.00h  C_max_=45.60±9.04ng/mL  T_1/2_=2.99±0.79h  AUC_0-t_=30.36±13.00h·ng/mL  CL=23.48±6.37L/h/kg  **Hyoscyamine 0.62g/kg:**  T_max_=0.88±0.14h  C_max_=124.86±30.02ng/mL  T_1/2_=2.08±0.21h  AUC_0-t_=409.23±254.71h·ng/mL  CL=1.88±0.48L/h/kg | p.o.  Male SD rats | - | [61] |
|  | *Strychni Semen* | Strychnine,  Brucine | Strychnine,  Brucine | **Strychnine 0.4mg/kg:**  T_max_=0.21±0.04h  C_max_=29.1±7.8µg/L  T_1/2_=0.93±0.25h  AUC_0-t_=44.3±16.5h·µg/L  AUC_0-∞_=46.4±16.8h·µg/L  CL=10.0±4.9L/h/kg  MRT_0-∞_=1.5±0.33h  **Strychnine 1.0mg/kg:**  T_max_=0.40±0.22h  C_max_=69.1±22.6µg/L | i.g.  Male SD rats | Brucine i.v.  LD_50_=12mg/kg | [83]  [84] |

Continue Table S1

| Toxicity level | Latin name | Active substances | Toxic substances | PK parameters | Animal model | Toxicology | Refs |
| --- | --- | --- | --- | --- | --- | --- | --- |
| Highly toxic | *Strychni Semen* | Strychnine,  Brucine | Strychnine,  Brucine | **Strychnine 1.0mg/kg:**  T_1/2_=1.0±0.19h  AUC_0-t_=135±43.1h·µg/L  AUC_0-∞_=136±43.2h·µg/L  CL=8.1±2.9L/h/kg  MRT_0-∞_=1.7±0.44h  **Brucine 0.2554mg/kg:**  T_max_=0.24±0.13h  C_max_=4.9±1.3µg/L, T_1/2_=2.8±1.9h  AUC_0-t_=14.3±6.9h·µg/L  AUC_0-∞_=21.2±10.7h·µg/L  CL=16.1±10.6L/h/kg  MRT_0-∞_=4.5±2.8h  **Brucine 0.6385mg/kg:**  T_max_=0.14±0.04h  C_max_=10.8±2.8µg/L  T_1/2_=4.4±2.8h  AUC_0-t_=27.3±10.6h·µg/L  AUC_0-∞_=44.1±20.4h·µg/L  CL=17.4±8.3L/h/kg  MRT_0-∞_=6.1±3.8h | i.g.  Male SD rats | Brucine i.v.  LD_50_=12mg/kg | [83][84] |
|  | *Strychni Semen Pulveratum* | Strychnine,  Brucine | Strychnine,  Brucine | **Strychnine 0.4mg/kg:**  T_max_=0.21±0.04h | i.g.  Male SD rats | Brucine i.v.  LD_50_=12mg/kg | [83][84] |

Continue Table S1

| Toxicity level | Latin name | Active substances | Toxic substances | PK parameters | Animal model | Toxicology | Refs |
| --- | --- | --- | --- | --- | --- | --- | --- |
| Highly toxic | *Strychni Semen Pulveratum* | Strychnine,  Brucine | Strychnine,  Brucine | **Strychnine 0.4mg/kg:**  C_max_=29.1±7.8µg/L  T_1/2_=0.93±0.25h  AUC_0-t_=44.3±16.5h·µg/L  AUC_0-∞_=46.4±16.8h·µg/L  CL=10.0±4.9L/h/kg  MRT_0-∞_=1.5±0.33h  **Strychnine 1.0mg/kg:**  T_max_=0.40±0.22h  C_max_=69.1±22.6µg/L, T_1/2_=1.0±0.19h  AUC_0-t_=135±43.1h·µg/L  AUC_0-∞_=136±43.2h·µg/L  CL=8.1±2.9L/h/kg  MRT_0-∞_=1.7±0.44h  **Brucine 0.2554mg/kg:**  T_max_=0.24±0.13h  C_max_=4.9±1.3µg/L  T_1/2_=2.8±1.9h  AUC_0-t_=14.3±6.9h·µg/L  AUC_0-∞_=21.2±10.7h·µg/L  CL=16.1±10.6L/h/kg  MRT_0-∞_=4.5±2.8h | i.g.  Male SD rats | Brucine i.v.  LD_50_=12mg/kg | [83][84] |

Continue Table S1

| Toxicity level | Latin name | Active substances | Toxic substances | PK parameters | Animal model | Toxicology | Refs |
| --- | --- | --- | --- | --- | --- | --- | --- |
| Highly toxic | *Strychni Semen Pulveratum* | Strychnine,  Brucine | Strychnine,  Brucine | **Brucine 0.6385mg/kg:**  T_max_=0.14±0.04h  C_max_=10.8±2.8µg/L  T_1/2_=4.4±2.8h  AUC_0-t_=27.3±10.6h·µg/L  AUC_0-∞_=44.1±20.4h·µg/L  CL=17.4±8.3L/h/kg  MRT_0-∞_=6.1±3.8h | i.g.  Male SD rats | Brucine i.v.  LD_50_=12mg/kg | [83][84] |
|  | *Crotonis Fructus* | Crotonoside,  Phorbol,  Crotonaldehyde | Crotonoside,  Phorbol,  Crotonaldehyde | **Crotonoside 12.5mg/kg:**  AUC_0-t_=34.3±10.4µg/mL/min  AUC_0-∞_=38.3±9.7µg/mL/min  C_max_=4.35±1.72µg/mL  MRT_0-t_=8.34±2.77min  MRT_0-∞_=19.79±8.20min  T_1/2_=30.77±7.44min  V_z_=16.83±8.18mL/kg  CL=0.346±0.096mL/min/kg  **Crotonoside 25.0mg/kg:**  AUC_0-t_=125.5±45.9µg/mL/min  AUC_0-∞_=136.8±50.3µg/mL/min  C_max_=17.28±7.61µg/mL  MRT_0-t_=6.65±2.37min  MRT_0-∞_=18.75±4.04min | i.v.  SD rats | - | [85] |

Continue Table S1

| Toxicity level | Latin name | Active substances | Toxic substances | PK parameters | Animal model | Toxicology | Refs |
| --- | --- | --- | --- | --- | --- | --- | --- |
| Highly toxic | *Crotonis Fructus* | Crotonoside,  Phorbol,  Crotonaldehyde | Crotonoside,  Phorbol,  Crotonaldehyde | **Crotonoside 25.0mg/kg:**  T_1/2_=49.43±18.76min  V_z_=15.67±6.56mL/kg  CL=0.206±0.077mL/min/kg  **Crotonoside 50.0mg/kg:**  AUC_0-t_=353.4±117.6µg/mL/min  AUC_0-∞_=390.7±129.8µg/mL/min  C_max_=58.32±16.29µg/mL  MRT_0-t_=8.47±1.47min  MRT_0-∞_=22.20±4.96min  T_1/2_=53.26±13.73min  V_z_=10.79±2.12mL/kg  CL=0.140±0.046mL/min/kg | i.v.  SD rats | - | [85] |
|  | *Crotonis Semen Pulveratum* | Crotonoside,  Phorbol,  Crotonaldehyde | Crotonoside,  Phorbol,  Crotonaldehyde | **Crotonoside 12.5mg/kg:**  AUC_0-t_=34.3±10.4µg/mL/min  AUC_0-∞_=38.3±9.7µg/mL/min  C_max_=4.35±1.72µg/mL  MRT_0-t_=8.34±2.77min  MRT_0-∞_=19.79±8.20min  T_1/2_=30.77±7.44min  V_z_=16.83±8.18mL/kg  CL=0.346±0.096mL/min/kg | i.v.  SD rats | - | [85] |

Continue Table S1

| Toxicity level | Latin name | Active substances | Toxic substances | PK parameters | Animal model | Toxicology | Refs |
| --- | --- | --- | --- | --- | --- | --- | --- |
| Highly toxic | *Crotonis Semen Pulveratum* | Crotonoside,  Phorbol,  Crotonaldehyde | Crotonoside,  Phorbol,  Crotonaldehyde | **Crotonoside 25.0mg/kg:**  AUC_0-t_=125.5±45.9µg/mL/min  AUC_0-∞_=136.8±50.3µg/mL/min  C_max_=17.28±7.61µg/mL  MRT_0-t_=6.65±2.37min  MRT_0-∞_=18.75±4.04min  T_1/2_=49.43±18.76min  V_z_=15.67±6.56mL/kg  CL=0.206±0.077mL/min/kg  **Crotonoside 50.0mg/kg:**  AUC_0-t_=353.4±117.6µg/mL/min  AUC_0-∞_=390.7±129.8µg/mL/min  C_max_=58.32±16.29µg/mL  MRT_0-t_=8.47±1.47min  MRT_0-∞_=22.20±4.96min  T_1/2_=53.26±13.73min  V_z_=10.79±2.12mL/kg  CL=0.140±0.046mL/min/kg | i.v.  SD rats | - | [85] |
|  | *Hydrargyri Oxydum Rubrum* | HgO | Hg | **Tianwangbuxin pills(bioaccessible):** SGF=0.59µg/g, SIF=0.63µg/g,  Bioaccessibility=0.016%, MDE=22.0µg/day | Simulated gastrie fluid,  Simulated intestinal fluid | - | [57] |

Continue Table S1

| Toxicity level | Latin name | Active substances | Toxic substances | PK parameters | Animal model | Toxicology | Refs |
| --- | --- | --- | --- | --- | --- | --- | --- |
| Highly toxic | *Mylabris* | Cantharidin | Cantharidin | **Cantharidin i.v. 34µg/kg:**  AUC=203.5±23.8h·µg/L  CL=168.8±18.6ml/h/kg  T1-2 =0.69±0.03h  **Cantharidin p.o. 102µg/kg:**  AUC=160.4±26.9h·µg/L  CL=649.1±97.7ml/h/kg  T1-2 =0.38±0.1h | i.v. p.o.  Beagle dog | - | [86] |
|  | *Aconiti Kusnezoffii Radix* | Aconitine,  Mesaconine,  Hypaconitine | Aconitine,  Mesaconine,  Hypaconitine | **Aconitine 0.5g/kg:**  AUC=13.47±3.28µg/L/h  T_max_=4.38±3.11h  C_max_=1.28±0.34μg/L；  CL=4.62±01.29L/h/kg  **Mesaconine 0.5g/kg:**  AUC=26.39±5.42µg/L/h  T_max_=2.18±1.40h  C_max_=2.66±0.50μg/L  CL=9.89±1.93L/h/kg  **Hypaconitine 0.5g/kg:**  AUC=62.90±26.89µg/L/h  T_max_=2.14±0.90h  C_max_=6.37±1.96μg/L  CL=4.02±1.80L/h/kg | p.o.  Male SD rats | - | [32] |

Continue Table S1

| Toxicity level | Latin name | Active substances | Toxic substances | PK parameters | Animal model | Toxicology | Refs |
| --- | --- | --- | --- | --- | --- | --- | --- |
| Highly toxic | *Rhododendri Mollis Flos* | RhodojaponinII,  RhodojaponinIII | RhodojaponinII,  Rhodojaponin III | **RhodojaponinII 2.1g/kg:**  T_max_=1.17±0.61h  C_max_=34.27±9.17ng/mL  T_1/2_=3.88±1.70h  AUC_0-t_=213.39±59.03h·ng/mL  AUC_0-∞_=251.32±85.05h·ng/mL  CL=9071.18±2612.86L/h/kg  V_z_=48721.77±18876.08L/kg  MRT_0-t_=4.46±0.27h  **RhodojaponinIII 2.1g/kg:**  T_max_=1.33±0.41h  C_max_=4.91±1.40ng/mL  T_1/2_=8.65±4.03h  AUC_0-t_=22.38±5.55h·ng/mL  AUC_0-∞_=40.47±13.25h·ng/mL  CL=55179.52±14094.94L/h/kg  V_z_=633384.85±224369.35L/kg  MRT_0-t_=4.14±0.75h | i.g.  Male SD rats | - | [87] |

Note: TCMs, tradional Chinese medicines; PK, Pharmacokinetics; SD, Sprague-Dawley; SGF, Simulated gastrie fluid; SIF, Simulated intestinal fluid; i.g., intragastric administration,; per os,p.o.; i.p., intraperitoneal injection; i.v., intravenous injection; i.m., intramuscular injection.

Table S2 Q-Markers and their PK and clinical relevance of TCMs.

| TCM product | Q-Markers | ADME evidence | PK parameters | Pharmacological outcomes | Refs |
| --- | --- | --- | --- | --- | --- |
| Guizhi fuling capsule | Gallic acid,  Amygdalin,  Albiﬂorin,  Prunasin,  Cinnamic acid | Compared with the normal group, the absorption, distribution, and elimination of active components in the dysmenorrhea model group showed significant differences | **Control:**  **Gallic acid:** AUC_0-t_=4622.3±834.9µg/L/h  MRT_0-t_=4.0±0.8h, C_max_=1166.9±145.6µg/L  T_1/2_=2.6±1.1h, T_max_=1.4±0.3h  CL_Z/F_=5.2±0.9L/h/kg, V_Z/F_=18.4±5.1L/kg  **Amygdalin:** AUC_0-t_=6607.73±818.1µg/L/h  MRT_0-t_=4.4±1.1h, C_max_=1701.7±360.8µg/L  T_1/2_=4.2±2.5h, T_max_=1.5±0.0h  CL_Z/F_=7.2±1L/h/kg, V_Z/F_=41.2±19.5L/kg  **Albiﬂorin:** AUC_0-t_=2123.7±455.6µg/L/h  MRT_0-t_=4.7±1.1h, C_max_=549.2±63.9µg/L  T_1/2_=3.8±2.0h, T_max_=1.5±0.0h  CL_Z/F_=11.4±2.4L/h/kg, V_Z/F_=58.5±21.3L/kg  **Prunasin:** AUC_0-t_=16960.8±2755.4µg/L/h  MRT_0-t_=4.4±1.1h  C_max_=3752.5±585.1µg/L  T_1/2_=2.6±1.7h, T_max_=2.0±0.0h  CL_Z/F_=7.1±0.7L/h/kg, V_Z/F_=44.2±22.1L/kg  **Cinnamic acid:** AUC_0-t_=5330.8±1604.9µg/L/h  MRT_0-t_=3.1±0.1h  C_max_=2942.8±763.9µg/L  T_1/2_=3.4±0.4h, T_max_=0.2±0.1h  CL_Z/F_=0.7±0.2L/h/kg  V_Z/F_=3.8±1.1L/kg | Guizhi fuling capsule can effectively treat primary dysmenorrhea by regulating prostaglandin synthesis, improving blood circulation, and inhibiting blood coagulation | [88] |

Continue Table S2

| TCM product | Q-Markers | ADME evidence | PK parameters | Pharmacological outcomes | Refs |
| --- | --- | --- | --- | --- | --- |
| Guizhi fuling capsule | Gallic acid,  Amygdalin,  Albiﬂorin,  Prunasin,  Cinnamic acid | Compared with the normal group, the absorption, distribution, and elimination of active components in the dysmenorrhea model group showed significant differences | **Model:**  **Gallic acid:** AUC_0-t_=6748.8±1086.2µg/L/h  MRT_0-t_=4.8±1.0h, C_max_=1534.9±145.7µg/L  T_1/2_=4.7±2.9h, T_max_=2.0±0.0h  CL_Z/F_=3.4±0.6L/h/kg, V_Z/F_=22.2±11.6L/kg  **Amygdalin:** AUC_0-t_=6801.6±1330.0µg/L/h  MRT_0-t_=4.2±0.8h, C_max_=1792.6±238.6µg/L  T_1/2_=5.3±4.9h,T_max_=2.0±0.0h  CL_Z/F_=7.0±1.2L/h/kg  V_Z/F_=55.9±54.7L/kg  **Albiﬂorin:** AUC_0-t_=3238.7±430.9µg/L/h  MRT_0-t_=5.2±0.3h, C_max_=653.2±30.9µg/L  T_1/2_=5.9±4.1h, T_max_=2.0±0.0h  CL_Z/F_=7.4±0.9L/h/kg, V_Z/F_=63.3±44.4L/kg  **Prunasin:** AUC_0-t_=39229.5±4702.9µg/L/h  MRT_0-t_=5.7±0.6h, C_max_=6283.3±721.2µg/L  T_1/2_=3.1±0.6h, T_max_=4.0±0.0h  CL_Z/F_=4.2±1.1L/h/kg  V_Z/F_=55.3±31.1L/kg  **Cinnamic acid:** AUC_0-t_=7176.6±1974.2µg/L/h  MRT_0-t_=5.4±0.5h, C_max_=3439.9±988.9µg/L  T_1/2_=6.4±2.7h, T_max_=0.2±0.0h  CL_Z/F_=0.6±0.2L/h/kg  V_Z/F_=5.0±1.7L/kg | Guizhi fuling capsule can effectively treat primary dysmenorrhea by regulating prostaglandin synthesis, improving blood circulation, and inhibiting blood coagulation | [88] |

Continue Table S2

| TCM product | Q-Markers | ADME evidence | PK parameters | Pharmacological outcomes | Refs |
| --- | --- | --- | --- | --- | --- |
| Shengjiang xiexin decoction | Baicalin,  Baicalein,  Wogonoside,  Wogonin,  Liquiritigenin,  Isoliquiritigenin,  Norwogonin,  Oroxylin A,  Dihydrobaicalin,  Chrysin,  Glycyrrhizic acid,  Glycyrrhetinic acid,  Oroxylin A 7-O-glucuronide,  Liquiritin,  Isoliquiritin | A total of 142 Shengjiang xiexin decoction-related exogenous components, including 77 prototype compounds and 65 metabolites, were identified by UHPLC-Q-Orbitrap HRMS | **Baicalin:** AUC_0-t_=24533.34±3868.20h·ng/mL  AUC_0-∞_=26206.44±5779.58h·ng/mL  C_max_=1736.67±286.89ng/mL  MRT=10.37±1.14h, T_1/2_=4.98±1.73h  V_d_=106.90±48.53L, CL=13.03±4.16L/h  **Oroxylin A:** AUC_0-t_=485.62±189.19h·ng/mL  AUC_0-∞_=519.22±248.70h·ng/mL  C_max_=61.60±19.30ng/mL, MRT=9.12±1.32h  T_1/2_=2.48±1.09h, V_d_=36.34±11.66L  CL=9.67±4.69L/h  **Liquiritigenin:** AUC_0-t_=79.15±18.67h·ng/mL  AUC_0-∞_=81.11±19.20h·ng/mL  C_max_=51.09±19.86ng/mL, MRT=4.40±0.75h  T_1/2_=4.26±0.80h, V_d_=2456.33±906.33L  CL=462.05±159.99L/h  **Oroxylin A 7-O-glucuronide:** CL=67.69±35.78L/h  AUC_0-t_=2310.48±934.89h·ng/mL  AUC_0-∞_=2835.94±1429.99h·ng/mL  C_max_=208.08±75.87ng/mL, MRT=9.89±1.19h  T_1/2_=7.11±2.85h, V_d_=620.57±267.58L  **Wogonoside:** AUC_0-t_=6725.48±2006.50h·ng/mL  AUC_0-∞_=8486.33±3875.68h·ng/mL  C_max_=493.11±188.22ng/mL, MRT=10.07±1.23h  T_1/2_=6.60±3.66h, V_d_=295.46±109.60L | Shengjiang xiexin decoction alleviates intestinal injury by regulating the Mrp-2 transporter, thereby reducing the intestinal exposure of CPT-11 and its metabolites | [89] |

Continue Table S2

| TCM product | Q-Markers | ADME evidence | PK parameters | Pharmacological outcomes | Refs |
| --- | --- | --- | --- | --- | --- |
| Shengjiang xiexin decoction | Baicalin,  Baicalein,  Wogonoside,  Wogonin,  Liquiritigenin,  Isoliquiritigenin,  Norwogonin,  Oroxylin A,  Dihydrobaicalin,  Chrysin,  Glycyrrhizic acid,  Glycyrrhetinic acid,  Oroxylin A 7-O-glucuronide,  Liquiritin,  Isoliquiritin | A total of 142 Shengjiang xiexin decoction-related exogenous components, including 77 prototype compounds and 65 metabolites, were identified by UHPLC-Q-Orbitrap HRMS | **Wogonoside:** CL=38.78±19.20L/h  **Wogonin:** AUC_0-t_=562.51±247.19h·ng/mL  AUC_0-∞_=619.97±326.88h·ng/mL  C_max_=58.59±19.57ng/mL, MRT=9.33±1.39h  T_1/2_=4.33±2.44h, V_d_=42.55±9.54L  CL=10.14±5.15L/h  **Glycyrrhizic acid:**  AUC_0-t_=493.31±197.03h·ng/mL  AUC_0-∞_=536.36±230.77h·ng/mL  C_max_=207.87±85.68ng/mL, MRT=5.66±1.44h  T_1/2_=4.35±2.18h, V_d_=595.49±332.39L  CL=88.03±46.12L/h  **Isoliquiritin:** AUC_0-t_=5.64±2.73h·ng/mL  AUC_0-∞_=7.63±3.19h·ng/mL  C_max_=5.56±1.80ng/mL, MRT=0.95±0.50h  T_1/2_=1.10±0.43h, V_d_=2851.59±915.11L  CL=1567.99±536.83L/h  **Glycyrrhetinic acid:**  AUC_0-t_=6256.37±2459.56h·ng/mL  AUC_0-∞_=6899.12±2787.93h·ng/mL  C_max_=609.33±251.27ng/mL, MRT=10.36±1.00h  T_1/2_=5.06±1.19h, V_d_=54.16±34.63L  CL=7.48±4.24L/h | Shengjiang xiexin decoction alleviates intestinal injury by regulating the Mrp-2 transporter, thereby reducing the intestinal exposure of CPT-11 and its metabolites | [89] |

Continue Table S2

| TCM product | Q-Markers | ADME evidence | PK parameters | Pharmacological outcomes | Refs |
| --- | --- | --- | --- | --- | --- |
| Shuangshen pingfei formula | Mangiferin,  Salvianolic acid B,  Tanshinone IIA,  Naringin,  Glycyrrhizic acid | After oral administration of Shuangshen pingfei formula to rats, the plasma concentration changes of nine Q-marker candidates were measured to evaluate their absorption | **Mangiferin 5g/kg:** T_max_=1.0h  C_max_=67.09±8.68ng/mL, T_1/2_=1.8±0.04h  AUC_0-t_=220.94±15.67h·ng/mL, MRT=1.42h  **Salvianolic acid B 5g/kg:** T_max_=1.5h  C_max_=31.30±6.43ng/mL, T_1/2_=1.5±0.03h  AUC_0-t_=84.04±5.55h·ng/mL, MRT=1.21h  **Tanshinone IIA 5g/kg:** T_max_=1.5h  C_max_=71.77±3.79ng/mL, MRT=1.46h  T_1/2_=2.0±0.04h, AUC_0-t_=260.31±20.18h·ng/mL  **Naringin 5g/kg:** T_max_=0.5h  C_max_=89.42±14.14ng/mL, T_1/2_=1.0±0.03h  AUC_0-t_=302.93±21.04h·ng/mL, MRT=0.66h  **Glycyrrhizic acid 5g/kg:** T_max_=1.0h  C_max_=35.16±4.83ng/mL, T_1/2_=1.6±0.05h  AUC_0-t_=136.02±11.27h·ng/mL, MRT=1.62h | Shuangshen pingfei formula significantly attenuates lung inflammation, fibrosis, rats by downregulating fibrosis-related targets, | [90] |
| Tripterygium glycosides tablet | Wilforine | Wilforine exhibits dose-and time-dependent characteristics in C_max_ and AUC(0–tn), indicating that it can be absorbed by the human body. Studies have shown that triptolide may be partially metabolized via CYP3A4 | **Wilforine 30mg/kg**  **Control:** T_max_=4.17±2.23h  C_max_=52.50±6.18ng/mL  AUC_0-t_=671.01±128.12ng/mL/h  MRT=8.40±0.29h, V_Z/F_=0.04±0.01L  **Model-14day:** T_max_=4.67±3.06h  C_max_=62.22±23.78ng/mL  AUC_0-t_=678.61±164.10ng/mL/h  MRT=7.18±1.20h, V_Z/F_=0.03±0.01L | Tripterygium glycosides tablets have a therapeutic effect on adjuvant arthritis and can alleviate joint swelling | [91] |

Continue Table S2

| TCM product | Q-Markers | ADME evidence | PK parameters | Pharmacological outcomes | Refs |
| --- | --- | --- | --- | --- | --- |
| Tripterygium glycosides tablet | Wilforine | Wilforine exhibits dose-and time-dependent characteristics in C_max_ and AUC(0–tn), indicating that it can be absorbed by the human body. Studies have shown that triptolide may be partially metabolized via CYP3A4 | **Model-21day:** T_max_=2.00±0.00h  C_max_=139.90±21.88ng/mL  AUC_0-t_=1469.08±415.47ng/mL/h  MRT=5.69±1.96h, V_Z/F_=0.01±0.01L | Tripterygium glycosides tablets have a therapeutic effect on adjuvant arthritis and can alleviate joint swelling | [91] |
| Danlou tablet | Puerarin,  Alisol A,  Daidzein,  Paeoniflorin,  Tanshinone IIA | A total of 110 Danlou tablet-related compounds, including 35 prototypes and 75 metabolites, were identified by UHPLC-Q/TOF-MS and UHPLC-TQ-MS | **Puerarin 0.9g/kg/day:** C_max_=27.24±2.47ng/mL  T_1/2_=17.13±4.31h, T_max_=3.25±1.12h  MRT_0-t_=11.27±0.90h, MRT_0-∞_=20.10±4.02h  AUC_0-t_=248.21±38.39ng·h/mL  AUC_0-∞_=287.14±38.83ng·h/mL  **Alisol A 0.9g/kg/day:** C_max_=5.74±2.37ng/mL  T_1/2_=47.78±20.02h, T_max_=1.00±0.00h  MRT_0-t_=16.72±3.96h  MRT_0-∞_=65.33±26.11h  AUC_0-t_=54.57±9.38ng·h/mL  AUC_0-∞_=107.88±17.72ng·h/mL  **Daidzein 0.9g/kg/day:** T_1/2_=23.38±9.93h  C_max_=4.91±0.80ng/mL, T_max_=1.58±1.28h  MRT_0-t_=7.83±0.42h, MRT_0-∞_=23.58±7.67h  AUC_0-t_=23.50±3.68ng·h/mL  AUC_0-∞_=35.18±9.44ng·h/mL | Danlou tablet possesses strong cardioprotective activity, and the PI3K/AKT pathway has been demonstrated to be a key mechanism through which Danlou tablet treats coronary heart disease | [92] |

Continue Table S2

| TCM product | Q-Markers | ADME evidence | PK parameters | Pharmacological outcomes | Refs |
| --- | --- | --- | --- | --- | --- |
| Danlou tablet | Puerarin,  Alisol A,  Daidzein,  Paeoniflorin,  Tanshinone IIA | A total of 110 Danlou tablet-related compounds, including 35 prototypes and 75 metabolites, were identified by UHPLC-Q/TOF-MS and UHPLC-TQ-MS | **Paeoniflorin 0.9g/kg/day:** C_max_=77.86±8.64ng/mL  T_1/2_=4.74±1.60h, T_max_=0.46±0.12h  MRT_0-t_=3.83±0.53h, MRT_0-∞_=5.83±1.39h  AUC_0-t_=296.22±44.26ng·h/mL  AUC_0-∞_=333.04±49.82ng·h/mL  **Tanshinone IIA 0.9g/kg/day:** C_max_=1.98±0.61ng/mL  T_1/2_=50.72±14.97h, T_max_=1.58±0.57h  MRT_0-t_=15.73±2.77h, MRT_0-∞_=70.06±19.64h  AUC_0-t_=20.95±4.28ng·h/mL  AUC_0-∞_=46.92±17.51ng·h/mL | Danlou tablet possesses strong cardioprotective activity, and the PI3K/AKT pathway has been demonstrated to be a key mechanism through which Danlou tablet treats coronary heart disease | [92] |
| Qingzao jiufei decoction | Chlorogenic acid,  Methylophiopogonan-one A,  Methylophiopogonan-one B,  Sesamin,  Ursolic acid,  Amygdalin,  Liquiritin apioside,  Liquiritigenin,  Isoliquiritin | A total of 121 *in* *vitro* components and 33 *in* *vivo* prototype components were identified using UHPLC-ESI-Q/TOF-MS technology | **Control:**  **Chlorogenic acid:** T_1/2_=1.753×10±5.92h  AUC_0-t_=3.051×10^3^±0.441×10^3^µg/L/h  AUC_0-∞_=3.625×10^3^±0.502×10^3^µg/L/h  C_max_=1.525×10^3^±0.385×10^3^µg/L  T_max_=2.250×10^−1^±0.380×10^−1^h  CL_Z/F_=5.608±0.756L/h/kg  V_Z/F_=1.390×10^2^±0.461×10^2^L/kg  **Methylophiopogonanone A:** V_Z/F_=57.24±5.84L/kg  AUC_0-t_=3.944×10^3^±0.215×10^3^µg/L/h  AUC_0-∞_=4.195×10^3^±0.224×10^3^µg/L/h  T_1/2_=8.31±0.82h, T_max_=1.188±0.438h  C_max_=3.722×10^2^±0.471×10^2^µg/L  CL_Z/F_=4.780±0.272L/h/kg | Qingzao jiufei decoction exerts a systemic therapeutic effect against acute lung injury through multi-level and multi-pathway regulation, synergistically promoting anti-inflammatory, antioxidant, and metabolic homeostasis-restoring effects | [93] |

Continue Table S2

| TCM product | Q-Markers | ADME evidence | PK parameters | Pharmacological outcomes | Refs |
| --- | --- | --- | --- | --- | --- |
| Qingzao jiufei decoction | Chlorogenic acid,  Methylophiopogonan-one A,  Methylophiopogonan-one B,  Sesamin,  Ursolic acid,  Amygdalin,  Liquiritin apioside,  Liquiritigenin,  Isoliquiritin | A total of 121 *in* *vitro* components and 33 *in* *vivo* prototype components were identified using UHPLC-ESI-Q/TOF-MS technology | **Methylophiopogonanone B:**  AUC_0-t_=6.148×10^3^±0.300×10^3^µg/L/h  AUC_0-∞_=6.376×10^3^±0.333×10^3^µg/L/h  T_1/2_=7.394±0.774h, T_max_=1.156±0.399h  C_max_=5.616×10^2^±0.499×10^2^µg/L  CL_Z/F_=3.145±0.175L/h/kg  V_Z/F_=33.48±3.13L/kg  **Sesamin:** V_Z/F_=30.77±9.69L/kg  AUC_0-t_=1.114×10^4^±0.115×10^4^µg/L/h  AUC_0-∞_=1.331×10^4^±0.194×10^4^µg/L/h  T_1/2_=14.72±4.11h, T_max_=1.125±0.354h  C_max_=1.110×10^3^±0.214×10^3^µg/L  CL_Z/F_=1.528±0.206L/h/kg  **Ursolic acid:** V_Z/F_=22.00±2.49L/kg  AUC_0-t_=1.292×10^4^±0.084×10^4^µg/L/h  AUC_0-∞_=1.359×10^4^±0.082×10^4^µg/L/h  T_1/2_=10.33±1.00h, T_max_=3.500±0.926h  C_max_=1.834×10^3^±0.079×10^3^µg/L  CL_Z/F_=1.476±0.088L/h/kg  **Amygdalin:** T_1/2_=30.24±9.00h  AUC_0-t_=4.908×10^3^±0.337×10^3^µg/L/h  AUC_0-∞_=6.411×10^3^±2.020×10^3^µg/L/h  C_max_=9.60×10^2^±2.06×10^2^µg/L  T_max_=2.440×10^-1^±0.177×10^-1^h | Qingzao jiufei decoction exerts a systemic therapeutic effect against acute lung injury through multi-level and multi-pathway regulation, synergistically promoting anti-inflammatory, antioxidant, and metabolic homeostasis-restoring effects | [93] |

Continue Table S2

| TCM product | Q-Markers | ADME evidence | PK parameters | Pharmacological outcomes | Refs |
| --- | --- | --- | --- | --- | --- |
| Qingzao jiufei decoction | Chlorogenic acid,  Methylophiopogonan-one A,  Methylophiopogonan-one B,  Sesamin,  Ursolic acid,  Amygdalin,  Liquiritin apioside,  Liquiritigenin,  Isoliquiritin | A total of 121 *in* *vitro* components and 33 *in* *vivo* prototype components were identified using UHPLC-ESI-Q/TOF-MS technology | **Amygdalin:** CL_Z/F_=3.472±1.018L/h/kg  V_Z/F_=1.281×10^2^±0.307×10^2^L/kg  **Liquiritin apioside:**  AUC_0-t_=4.040×10^3^±0.538×10^3^µg/L/h  AUC_0-∞_=4.376×10^3^±0.630×10^3^µg/L/h  T_1/2_=10.14±2.51h, T_max_=3.000×10^-1^±0.964×10^-1^h  C_max_=1.790×10^3^±0.113×10^3^µg/L  CL_Z/F_=4.650±0.630L/h/kg, V_Z/F_=67.54±15.50L/kg  **Liquiritigenin:**  AUC_0-t_=3.406×10^3^±0.470×10^3^µg/L/h  AUC_0-∞_=3.654×10^3^±0.529×10^3^µg/L/h  T_1/2_=9.48±1.89h, T_max_=7.788×10^−2^±1.450×10^−2^h  C_max_=1.362×10^3^±0.306×10^3^µg/L  CL_Z/F_=5.591±0.939L/h/kg  V_Z/F_=7.583×10^2^±1.648×10^2^L/kg  **Isoliquiritin:** AUC_0-t_=4.60×10^3^±0.656×10^3^µg/L/h  AUC_0-∞_=5.759×10^3^±0.671×10^3^µg/L/h  T_1/2_=1.839×10^1^±6.52h  C_max_=1.504×10^3^±0.307×10^3^µg/L  CL_Z/F_=3.513±0.388L/h/kg, V_Z/F_=92.7±30.3L/kg  T_max_=2.125×10^−1^±0.582×10^−1^h  **Model:**  **Chlorogenic acid:** V_Z/F_=41.56±6.73L/kg  AUC_0-t_=6.805×10^3^±0.916×10^3^µg/L/h | Qingzao jiufei decoction exerts a systemic therapeutic effect against acute lung injury through multi-level and multi-pathway regulation, synergistically promoting anti-inflammatory, antioxidant, and metabolic homeostasis-restoring effects | [93] |

Continue Table S2

| TCM product | Q-Markers | ADME evidence | PK parameters | Pharmacological outcomes | Refs |
| --- | --- | --- | --- | --- | --- |
| Qingzao jiufei decoction | Chlorogenic acid,  Methylophiopogonan-one A,  Methylophiopogonan-one B,  Sesamin,  Ursolic acid,  Amygdalin,  Liquiritin apioside,  Liquiritigenin,  Isoliquiritin | A total of 121 *in* *vitro* components and 33 *in* *vivo* prototype components were identified using UHPLC-ESI-Q/TOF-MS technology | **Chlorogenic acid:** CL_Z/F_=2.708±0.411L/h/kg  AUC_0-∞_=7.539×10^3^±1.157×10^3^µg/L/h  T_1/2_=10.77±1.98h, T_max_=2.563×10^−1^±0.177×10^−1^h  C_max_=1.159×10^3^±0.200×10^3^µg/L  **Methylophiopogonanone A:**  AUC_0-t_=3.352×10^3^±0.222×10^3^µg/L/h  AUC_0-∞_=3.620×10^3^±0.428×10^3^µg/L/h  T_1/2_=8.59±1.40h, T_max_=1.500±0.267h  C_max_=2.889×10^2^±0.299×10^2^µg/L  CL_Z/F_=5.587±0.602L/h/kg  V_Z/F_=66.90±24.67L/kg  **Methylophiopogonanone B:**  AUC_0-t_=5.218×10^3^±1.376×10^3^µg/L/h  AUC_0-∞_=5.271×10^3^±1.382×10^3^µg/L/h  T_1/2_=5.406±0.986h, T_max_=3.688±1.044h  C_max_=3.861×10^2^±0.342×10^2^µg/L  CL_Z/F_=4.092±1.369L/h/kg  V_Z/F_=31.12±8.33L/kg  **Sesamin:** AUC_0-t_=8.19×10^3^±0.47×10^3^µg/L/h  AUC_0-∞_=8.95×10^3^±0.71×10^3^µg/L/h  T_1/2_=10.34±1.7h, T_max_=1.625±0.231h  C_max_=7.952×10^2^±1.126×10^2^µg/L  CL_Z/F_=2.246±0.175L/h/kg, V_Z/F_=33.23±4.21L/kg  **Ursolic acid:** C_max_=1.522×10^3^±0.204×10^3^µg/L | Qingzao jiufei decoction exerts a systemic therapeutic effect against acute lung injury through multi-level and multi-pathway regulation, synergistically promoting anti-inflammatory, antioxidant, and metabolic homeostasis-restoring effects | [93] |

Continue Table S2

| TCM product | Q-Markers | ADME evidence | PK parameters | Pharmacological outcomes | Refs |
| --- | --- | --- | --- | --- | --- |
| Qingzao jiufei decoction | Chlorogenic acid,  Methylophiopogonan-one A,  Methylophiopogonan-one B,  Sesamin,  Ursolic acid,  Amygdalin,  Liquiritin apioside,  Liquiritigenin,  Isoliquiritin | A total of 121 *in* *vitro* components and 33 *in* *vivo* prototype components were identified using UHPLC-ESI-Q/TOF-MS technology | **Ursolic acid:** AUC_0-t_=9.75×10^3^±1.20×10^3^µg/L/h  AUC_0-∞_=1.026×10^4^±0.137×10^4^µg/L/h  T_1/2_=11.84±3.43h, T_max_=4.250±0.378h  CL_Z/F_=1.988±0.331L/h/kg, V_Z/F_=32.96±7.29L/kg  **Amygdalin:** AUC_0-t_=4.386×10^3^±0.403×10^3^µg/L/h  AUC_0-∞_=5.113×10^3^±0.385×10^3^µg/L/h  T_1/2_=14.17±2.32h, T_max_=2.563×10^-1^±0.177×10^-1^h  C_max_=8.23×10^2^±1.57×10^2^µg/L  CL_Z/F_=3.932±0.304L/h/kg, V_Z/F_=80.6±15.8L/kg  **Liquiritin apioside:**  AUC_0-t_=6.961×10^3^±0.775×10^3^µg/L/h  AUC_0-∞_=7.715×10^3^±0.894×10^3^µg/L/h  T_1/2_=10.98±2.26h, T_max_=2.938×10^-1^±0.563×10^-1^h  C_max_=1.290×10^3^±0.254×10^3^µg/L  CL_Z/F_=2.625±0.322L/h/kg, V_Z/F_=41.23±8.63L/kg  **Liquiritigenin:**  AUC_0-t_=3.096×10^3^±0.269×10^3^µg/L/h  AUC_0-∞_=3.299×10^3^±0.299×10^3^µg/L/h  T_1/2_=9.09±1.38h  T_max_=8.725×10^−2^±0.787×10^−2^h  C_max_=1.038×10^3^±0.220×10^3^µg/L  CL_Z/F_=6.108±0.561L/h/kg  V_Z/F_=7.968×10^2^±1.084×10^2^L/kg  **Isoliquiritin:**C_max_=1.180×10^3^±0.109×10^3^µg/L | Qingzao jiufei decoction exerts a systemic therapeutic effect against acute lung injury through multi-level and multi-pathway regulation, synergistically promoting anti-inflammatory, antioxidant, and metabolic homeostasis-restoring effects | [93] |

Continue Table S2

| TCM product | Q-Markers | ADME evidence | PK parameters | Pharmacological outcomes | Refs |
| --- | --- | --- | --- | --- | --- |
| Qingzao jiufei decoction | Chlorogenic acid,  Methylophiopogonan-one A,  Methylophiopogonan-one B,  Sesamin, Ursolic acid,  Amygdalin,  Liquiritin apioside,  Liquiritigenin,  Isoliquiritin | A total of 121 *in* *vitro* components and 33 *in* *vivo* prototype components were identified using UHPLC-ESI-Q/TOF-MS technology | **Isoliquiritin:** AUC_0-t_=5.856×10^3^±0.37×10^3^µg/L/h  AUC_0-∞_=6.454×10^3^±0.452×10^3^µg/L/h  T_1/2_=11.35±1.26h, T_max_=2.875×10^−1^±0.518×10^−1^h  CL_Z/F_=3.114±0.237L/h/kg, V_Z/F_=50.89±5.79L/kg | Qingzao jiufei decoction exerts a systemic therapeutic effect against acute lung injury through multi-level and multi-pathway regulation, synergistically promoting anti-inflammatory, antioxidant, and metabolic homeostasis-restoring effects | [93] |
| Taohong siwu decoction | Adenosine,  Hydroxysafflor yellow A,  Amygdalin,  Vanillic acid,  Caffeic acid | A total of 68 compounds were identified in Taohong siwu decoction using UPLC-Q-TOF-MS, and 16 major bioactive components were selected for pharmacokinetic studies | **Control:**  **Adenosine:** AUC_0-t_=370.99±73.06ng/L/h  AUC_0-∞_=539.46±417.25ng/L/h  MRT_0-t_=10.11±1.93h, MRT_0-∞_=20.85±10.87h  T_1/2_=15.73±8.23h, T_max_=0.92±0.21h  C_max_=41.45±21.94ng/L  **Hydroxysafflor yellow A:** MRT_0-t_=8.23±2.73h  AUC_0-t_=1673.19±380.72ng/L/h  AUC_0-∞_=1797.58±494.15ng/L/h  MRT_0-∞_=10.07±2.52h, T_1/2_=7.27±3.77h  T_max_=0.60±0.35h, C_max_=475.57±244.18 ng/L  **Amygdalin:** AUC_0-t_=370.66±54.24ng/L/h  AUC_0-∞_=760.01±61.69ng/L/h  MRT_0-t_=10.33±1.17h, MRT_0-∞_=33.70±26.97h | Taohong siwu decoction has the potential to treat blood stasis-related disorders and serve as an adjuvant therapy for other diseases, providing new insights for its clinical application | [94] |

Continue Table S2

| TCM product | Q-Markers | ADME evidence | PK parameters | Pharmacological outcomes | Refs |
| --- | --- | --- | --- | --- | --- |
| Taohong siwu decoction | Adenosine,  Hydroxysafflor yellow A,  Amygdalin,  Vanillic acid,  Caffeic acid | A total of 68 compounds were identified in Taohong siwu decoction using UPLC-Q-TOF-MS, and 16 major bioactive components were selected for pharmacokinetic studies | **Amygdalin:** T_1/2_=0.76±0.29h, T_max_=0.25±0.2h  C_max_=90.48±45.29ng/L  **Vanillic acid:** AUC_0-t_=395.91±56.52ng/L/h  AUC_0-∞_=732.58±142.92ng/L/h  MRT_0-t_=10.92±0.65h, MRT_0-∞_=30.97±8.02h  T_1/2_=21.00±5.76h, T_max_=0.14±0.09h  C_max_=48.97±15.82ng/L  **Caffeic acid:** AUC_0-t_=71.81±22.53ng/L/h  AUC_0-∞_=78.01±27.01ng/L/h  MRT_0-t_=7.44±1.39h,MRT_0-∞_=9.31±3.59h  T_1/2_=5.08±3.05h,T_max_=5.00±1.67h  C_max_=9.33±5.01ng/L  **Model:**  **Adenosine:**AUC_0-t_=146.89±67.71ng/L/h  AUC_0-∞_=373.45±67.82ng/L/h  MRT_0-t_=9.63±1.85h, MRT_0-∞_=52.21±69.78h  T_1/2_=8.03±4.90h, T_max_=1.06±0.83h  C_max_=15.45±3.71ng/L  **Hydroxysafflor yellow A:** MRT_0-t_=7.28±1.63h  AUC_0-t_=986.03±226.95ng/L/h  AUC_0-∞_=1374.97±276.37ng/L/h  MRT_0-∞_=18.77±8.66h, T_1/2_=15.75±6.56h  T_max_=0.71±0.1h, C_max_=413.60±220.29ng/L  **Amygdalin:** AUC_0-t_=348.91±31.36ng/L/h | Taohong siwu decoction has the potential to treat blood stasis-related disorders and serve as an adjuvant therapy for other diseases, providing new insights for its clinical application | [94] |

Continue Table S2

| TCM product | Q-Markers | ADME evidence | PK parameters | Pharmacological outcomes | Refs |
| --- | --- | --- | --- | --- | --- |
| Taohong siwu decoction | Adenosine,  Hydroxysafflor yellow A,  Amygdalin,  Vanillic acid,  Caffeic acid | A total of 68 compounds were identified in Taohong siwu decoction using UPLC-Q-TOF-MS, and 16 major bioactive components were selected for pharmacokinetic studies | **Amygdalin:** C_max_=54.75±23.42ng/L  AUC_0-∞_=1753.85±251.88ng/L/h  MRT_0-t_=10.69±0.76h, MRT_0-∞_=76.49±43.57h  T_1/2_=1.63±1.37h, T_max_=0.9±0.65h  **Vanillic acid:** AUC_0-t_=338.21±77.64ng/L/h  AUC_0-∞_=1272.54±299.03ng/L/h  MRT_0-t_=12.21±1.03h, MRT_0-∞_=69.88±25.78h  T_1/2_=48.58±18.43h, T_max_=0.25±0.29h  C_max_=30.58±8.57ng/L  **Caffeic acid:** AUC_0-t_=65.34±18.57ng/L/h  AUC_0-∞_=66.63±18.77ng/L/h  MRT_0-t_=5.85±0.76h, MRT_0-∞_=6.34±0.94h  T_1/2_=4.29±1.22h, T_max_=4.33±0.82h  C_max_=11.65±6.32ng/L | Taohong siwu decoction has the potential to treat blood stasis-related disorders and serve as an adjuvant therapy for other diseases, providing new insights for its clinical application | [94] |
| Qiliqiangxin capsule | Astragaloside,  Calycosin-7-glucoside,  Sinapine,  Ginsenoside Rg1 | - | **Astragaloside:** T_1/2_=8.14±4.84h  T_max_=4.00±0.24h, C_max_=18.71±6.53ng/mL  AUC_0-t_=64.29±11.17h·ng/mL  AUC_0-∞_=72.41±35.22h·ng/mL  **Calycosin-7-glucoside:** T_1/2_=4.83±3.58h  T_max_=4.00±0.00h, C_max_=8.75±0.98ng/mL  AUC_0-t_=52.02±8.77h·ng/mL  AUC_0-∞_=80.80±9.86h·ng/mL  **Sinapine:** T_1/2_=6.99±1.23h, T_max_=4.00±0.06h  C_max_=1.33±0.08ng/mL | Qiliqiangxin capsule exerts its therapeutic effect on chronic heart failure by inhibiting the excessive activation of the renin-angiotensin-aldosterone system and reducing the level of angiotensin II | [95] |

Continue Table S2

| TCM product | Q-Markers | ADME evidence | PK parameters | Pharmacological outcomes | Refs |
| --- | --- | --- | --- | --- | --- |
| Qiliqiangxin capsule | Astragaloside,  Calycosin-7-glucoside,  Sinapine,  Ginsenoside Rg1 | - | **Sinapine:** AUC_0-t_=5.05±1.64h·ng/mL  AUC_0-∞_=23.65±3.34h·ng/mL  **Ginsenoside Rg1:** T_1/2_=8.21±5.20h  T_max_=4.00±0.00h  C_max_=8.60±1.25ng/mL  AUC_0-t_=20.75±8.20h·ng/mL  AUC_0-∞_=39.34±11.33h·ng/mL | Qiliqiangxin capsule exerts its therapeutic effect on chronic heart failure by inhibiting the excessive activation of the renin-angiotensin-aldosterone system and reducing the level of angiotensin II | [95] |
| *Vladimiriae Radix* extract | Costunolide,  Dehydrocostus lactone | - | **Control:**  **Costunolide:** MRT_0-∞_=21.39±7.76h  AUC_0-t_=748.42±41.43mg/L/h  AUC_0-∞_=969.59±198.64mg/L/h, C_max_=65.25±11.75mg/L  T_1/2_=13.27±4.15h, T_max_=0.76±0.15h  CL_Z/F_=0.19±0.04L/h/kg, V_Z/F_=3.48±0.48L/kg  **Dehydrocostus lactone:** MRT_0-∞_=12.14±2.55h  AUC_0-t_=970.54±119.34mg/L·h, C_max_=96.41±16.32mg/L  AUC_0-∞_=1025.87±127.62mg/L·h  T_1/2_=7.95±2.59h, T_max_=6.00±0.00h  CL_Z/F_=0.18±0.02L/h/kg, V_Z/F_=2.02±0.62L/kg  **Model:**  **Costunolide:** MRT_0-∞_=9.73±1.89h  AUC_0-t_=2244.76±179.88mg/L/h  AUC_0-∞_=2410.84±163.24mg/L/h  T_1/2_=9.11±1.99h, T_max_=1.17±0.42h  C_max_=598.61±172.60mg/L | The *Vladimiriae Radix* extract exhibits significant therapeutic effects against cholestatic liver injury, and its mechanism of action may be related to the pharmacological activities of costunolide and dehydrocostus lactone, including anti-inflammatory, antioxidant, and bile acid metabolism-regulating effects | [96] |

Continue Table S2

| TCM product | Q-Markers | ADME evidence | PK parameters | Pharmacological outcomes | Refs |
| --- | --- | --- | --- | --- | --- |
| *Vladimiriae Radix* extract | Costunolide,  Dehydrocostus lactone | - | **Costunolide:** CL_Z/F_=0.08±0.01L/h/kg  V_Z/F_=0.99±0.24L/kg  **Dehydrocostus lactone:** MRT_0-∞_=10.27±2.55h  AUC_0-t_=2928.56±435.56mg/L/h  AUC_0-∞_=3082.00±455.71mg/L/h  T_1/2_=7.56±2.17h, T_max_=0.57±0.15h  C_max_=531.70±116.19mg/L  CL_Z/F_=0.06±0.01L/h/kg, V_Z/F_=0.64±0.16L/kg | The *Vladimiriae Radix* extract exhibits significant therapeutic effects against cholestatic liver injury, and its mechanism of action may be related to the pharmacological activities of costunolide and dehydrocostus lactone, including anti-inflammatory, antioxidant, and bile acid metabolism-regulating effects | [96] |
| *Scutellaria Extract* | Baicalin,  Baicalein,  Wogonin,  Wogonoside,  Oroxylin A | A total of 139 compounds were identified in the *Scutellaria Extract*, among which 35 and 41 compounds were detected in rat plasma and urine, respectively | **Control:**  **Baicalin:** C_max_=3744±755ng/L  T_max_=0.29±0.09h, T_1/2_=11.1±5.0h  AUC_0-24h_=18243±3793ng·h/mL  AUC_0-∞_=24940±5829ng·h/mL  **Baicalein:** C_max_=77.1±18.6ng/L  T_max_=0.13±0.11h, T_1/2_=25.4±11.2h  AUC_0-24h_=796±134ng·h/mL  AUC_0-∞_=1964±795ng·h/mL  **Wogonin:** C_max_=139±54.0ng/L  T_max_=0.20±0.08h, T_1/2_=8.2±2.2h  AUC_0-24h_=935±215ng·h/mL  AUC_0-∞_=1302±257ng·h/mL  **Wogonoside:** C_max_=875±237ng/L  T_max_=0.33±0.28h, T_1/2_=5.6±2.2h | *Scutellaria Extract* exhibits therapeutic effects on rats with viral pneumonia by alleviating lung injury, suppressing inflammatory responses, and regulating metabolic disorders | [97] |

Continue Table S2

| TCM product | Q-Markers | ADME evidence | PK parameters | Pharmacological outcomes | Refs |
| --- | --- | --- | --- | --- | --- |
| *Scutellaria Extract* | Baicalin,  Baicalein,  Wogonin,  Wogonoside,  Oroxylin A | A total of 139 compounds were identified in the *Scutellaria Extract*, among which 35 and 41 compounds were detected in rat plasma and urine, respectively | **Wogonoside:** AUC_0-24h_=9147±1859ng·h/mL  AUC_0-∞_=11297±2769ng·h/mL  **Oroxylin A:** C_max_=71.9±23.8ng/L, T_max_=0.21±0.07h  T_1/2_=8.7±3.1h, AUC_0-24h_=293±142ng·h/mL  AUC_0-∞_=370±163ng·h/mL  **Model:**  **Baicalin:** C_max_=2410±381ng/L, T_max_=0.34±0.25h  T_1/2_=10.9±4.8h, AUC_0-24h_=18730±3838ng·h/mL  AUC_0-∞_=32917±6665ng·h/mL  **Baicalein:** C_max_=59.9±19.7ng/L**,** T_max_=0.26±0.07h  T_1/2_=19.5±8.8h, AUC_0-24h_=843±35ng·h/mL  AUC_0-∞_=1455±385ng·h/mL  **Wogonin:** C_max_=57.3±15.9ng/L**,** T_max_=0.29±0.09h  T_1/2_=15.6±4.2h, AUC_0-24h_=667±153ng·h/mL  AUC_0-∞_=960±249ng·h/mL  **Wogonoside:** C_max_=401±85ng/L, T_max_=0.39±0.26h  T_1/2_=8.1±3.5h, AUC_0-24h_=5354±1320ng·h/mL  AUC_0-∞_=7561±2317ng·h/mL  **Oroxylin A:** C_max_= 26.7±8.5ng/L, T_max_=0.24±0.12h  T_1/2_=10.1±4.4h, AUC_0-24h_=244±109ng·h/mL  AUC_0-∞_=335±131ng·h/mL | *Scutellaria Extract* exhibits therapeutic effects on rats with viral pneumonia by alleviating lung injury, suppressing inflammatory responses, and regulating metabolic disorders | [97] |

Note: ADME, absorption, distribution, metabolism, and excretion.

**References:**

[1] Gao XY, Li XY, Zhang CY, Bai CY. Scopoletin: a review of its pharmacology, pharmacokinetics, and toxicity. Front Pharmacol, 2024, 15: 1268464. doi:10.3389/fphar.2024.1268464.

[2] Zeng Y, Li S, Wang X, Gong T, Sun X, Zhang Z. Validated LC-MS/MS Method for the Determination of Scopoletin in Rat Plasma and Its Application to Pharmacokinetic Studies. Molecules, 2015, 20(10): 18988-19001. doi:10.3390/molecules201018988.

[3] Yang HL, Li YF,Liu ZJ , Lu XY, Pan ZX. Simultaneous determination of 7 activeingredients in Erycibes Caulis with differentprocessing technology by QAMS. Chinese Journal of Pharmaceutical Analysis, 2024, 44(03): 395-404. doi:10.16155/j.0254-1793.2024.03.04.

[4] Liao ZF, Liu H, Lin Y, Lu H, Ma Q, Xie WB, et al. Research Progress on Main chemicalConstituents, Pharmacological Effects, andConservation of Murrayae Folium et Cacumen . Modern Chinese Medicine, 2023, 25(08): 1799-1814. doi:10.13313/j.issn.1673-4890.20221009003.

[5] Ni LJ, Liu XF, Hu X, Lin WZ, Xu HF, Yu LS. Research Progress on Chemical Composition ofTubiechong(Eupolyphaga Steleophaga). Chinese Archives of Traditional Chinese Medicine, 2024, 42(11): 170-176. doi:10.13193/j.issn.1673-7717.2024.11.033.

[6] Wang CC, Zuo BL, Peng X, Zhu JG, Zhou N, Li HW, et al. Quality standard for Gleditsiae sinensis Fructus. Chinese Traditional Patent Medicine, 2023, 45(07): 2259-2264.

[7] Zan K, Zhou Y, Li YL, Wang Y, Jin HY, Zuo TT, et al. Risk Assessment and Determination ofHepatotoxic Pyrrolizidine Alkaloids inEuphorbiae Hirtae Herba. Chinese Pharmaceutical Journal, 2021, 56(22): 1829-1833.

[8] Chang H, Wang C, Gong L, Zhang Y, Liang C, Liu H. An overview of Fructus Meliae Toosendan: Botany, traditional uses, phytochemistry, pharmacology and toxicology. Biomed Pharmacother, 2023, 157: 113795. doi:10.1016/j.biopha.2022.113795.

[9] Li PZ, Shi XC,Xu ZH, Li JF, Sun GZ, Qing BY. Pharmacological and toxicological studies of toosendanin. Chinese Traditional and Herbal Drugs, 1982, 13(07): 29-32.

[10] Yu JY, Wang QW, Shi L, Zhao J, Zhao RT, Zhang Y, et al. Pharmacokinetic study of toosendanin inToosendan Fructus extract in rats. China Medical Herald, 2019, 16(30): 21-25.

[11] Xu MH, Qiu WT, Jin L, Li MF, Tong HY. Research progress on bioactive constituents andpharmacological effects of Tibetan medicineSinopodophylli Fructus. China Journal of Chinese Materia Medica, 2024, 49(10): 2640-2647. doi:10.19540/j.cnki.cjcmm.20230602.201.

[12] Shang MY, Xu LS, Li P,Xu GJ,Wang YX,Cai SQ. Study on pharmacodynamics of chinese herbaldrug Guijiu and its lignan. Chinese Traditional and Herbal Drugs, 2002, (08): 52-54.

[13] Xeu M, Zhu RR, Qing LL,Li FJ, Liu ZX, Sun XY, et al. Study on the Antitumor Activity and Pharmacokinetics of Podophyllotoxin Solid Lipid Nanoparticles. Scientia Sinica(Chimica), 2009, 39(02): 159-164.

[14] Jia HJ, Xu H, Huang Y, Yang MH, Yang XJ, Zhao SM. Research progress on the chemical compositionand medicinal use of leeches. China Feed, 2024, (09): 162-166. doi:10.15906/j.cnki.cn11-2975/s.2023040038-04.

[15] Ruan JX, Liu SJ, Wang C, Wang XF, Yan JH, Liu SM. Research progress and prediction of qualitymarkers of Menispermi Rhizoma. China Journal of Chinese Materia Medica, 2024, 49(18): 4847-4859. doi:10.19540/j.cnki.cjcmm.20240605.202.

[16] Zhao XL, Li T, Liu Y, Zhang MY, Chen Y, Cui Y, et al. Pharmacokinetic analysis of sinomenine based on the automatic blood collection system and HPLC-QQQ-MS. Chinese Journal of Experimental Traditional Medical Formulae, 2015, 21(14): 66-70. doi:10.13422/j.cnki.syfjx.2015140066.

[17] Wei Y, Li Y, Wang S, Xiang Z, Li X, Wang Q, et al. Phytochemistry and pharmacology of Armeniacae semen Amarum: A review. J Ethnopharmacol, 2023, 308: 116265. doi:10.1016/j.jep.2023.116265.

[18] Liu Y, He Y, Wang F, Xu R, Yang M, Ci Z, et al. From longevity grass to contemporary soft gold: Explore the chemical constituents, pharmacology, and toxicology of Artemisia argyi H.Lév. & vaniot essential oil. J Ethnopharmacol, 2021, 279: 114404. doi:10.1016/j.jep.2021.114404.

[19] Hou M Z, Chen L L, Chang C, Zan J F, Du S M. Pharmacokinetic and tissue distribution study of eight volatile constituents in rats orally administrated with the essential oil of Artemisiae argyi Folium by GC-MS/MS. J Chromatogr B Analyt Technol Biomed Life Sci, 2021, 1181: 122904. doi:10.1016/j.jchromb.2021.122904.

[20] Zou ZQ, Ning DS, Li LC, Fu YX, Pan ZH. Research Progress on chemical Constituentsand Pharmacological Activities of llliciumdifengpi. Guangxi Sciences, 2020, 27(04): 336-346. doi:10.13656/j.cnki.gxkx.20200924.009.

[21] Hong YL, Ma L, Wang YF, Sun JF, Hou GG, Zhao F, et al. Anthraquinones and triterpenoids from roots ofKnoxia roxburghii. China Journal of Chinese Materia Medica, 2014, 39(21): 4230-4233.

[22] Lu Q, Ma R, Yang Y, Mo Z, Pu X, Li C. Zanthoxylum nitidum (Roxb.) DC: Traditional uses, phytochemistry, pharmacological activities and toxicology. J Ethnopharmacol, 2020, 260: 112946. doi:10.1016/j.jep.2020.112946.

[23] Lu Q, Luo S, Shi Z, Yu M, Guo W, Li C. Nitidine chloride, a benzophenanthridine alkaloid from Zanthoxylum nitidum (Roxb.) DC., exerts multiple beneficial properties, especially in tumors and inflammation-related diseases. Front Pharmacol, 2022, 13: 1046402. doi:10.3389/fphar.2022.1046402.

[24] Li M, Wang C. Traditional uses, phytochemistry, pharmacology, pharmacokinetics and toxicology of the fruit of Tetradium ruticarpum: A review. J Ethnopharmacol, 2020, 263: 113231. doi:10.1016/j.jep.2020.113231.

[25] Hao SY, Shi GG, Luo WH, Li H, Gao FF, Zhang YM, et al. Pharmacokinetics of Xiangbei Injection inHybrid Dogs by HPLC-MS Assay. Traditional Chinese Drug Research and Clinical Pharmacology, 2010, 21(05): 509-511. doi:10.19378/j.issn.1003-9783.2010.05.019.

[26] Zhao BS, Gui HS, Zhu YD, Xu TH. Research Progress in Chemical Compoents, Pharmacological Effectiveness and Toxicity of Psammosilene tunicoides. Chinese Journal of Experimental Traditional Medical Formulae, 17(18): 288-291.

[27] Cui CY, Xiao L, Yang YT, Li Q. Research Progress in chemical Constituents andPharmacological Activities of Carotae Fructus. Liaoning Chemical Industry, 2020, 49(06): 651-654. doi:10.14029/j.cnki.issn1004-0935.2020.06.014.

[28] Kong LL, Li l, Du GH. Research progress of toxicity of Garden BalsamSeed. Pharmacology and Clinics of Chinese Materia Medica, 2018, 34(05): 155-156. doi:10.13412/j.cnki.zyyl.2018.05.039.

[29] Huang Y, Luo M, Wang Y, Li YQ. Progress in Plant Biology Research of FructusCnidii. Journal of Tropical and Subtropical Botany, 2020, 28(06): 644-650.

[30] Dong NN, Chen XL, Deng BL, Wang J. Study Advances in chemical Constituents andPharmacological Effects of Gleditsiae Fructusabnormalis. Journal of Guizhou University of Traditional Chinese Medicine, 2022, 44(05): 72-76. doi:10.16588/j.cnki.issn2096-8426.2022.05.016.

[31] Dong SR, Cao YG, Li K, Tian LQ, Meng XL, Shi YB, et al. Effects of different processing methods on oicomponents in Gleditsiae sinensis Fructus andGleditsiae Fructus Abnormalis. Chinese Traditional Patent Medicine, 2024, 46(06): 1809-1818.

[32] Yang L, Xie G, Wang Y, Li J, Zheng B, Zhu J, et al. Metabolic Behaviors of Aconitum Alkaloids in Different Concentrations of Aconiti Lateralis Radix Praeparata and Effects of Aconitine in Healthy Human and Long QT Syndrome Cardiomyocytes. Molecules, 2022, 27(13). doi:10.3390/molecules27134055.

[33] Yang XY, Zhang W, Yuan TY, Du GH. The historical cognition and evaluation ofCarpesii Fructus toxicity. Pharmacology and Clinics of Chinese Materia Medica, 2018, 34(05): 153-155. doi:10.13412/j.cnki.zyyl.2018.05.038.

[34] Dong ST, Xu LX, Gao JP, Li BX. Quality evaluation of DryopteridisCrassirhizomatis Rhizoma Carbonisatum basedon QAMS and EW-TOPSIS method. Chinese Traditional and Herbal Drugs, 2024, 55(07): 2397-2404.

[35] Xiong W, Zhao Y, Cheng JJ, Zhang ML, Sun ZW, Luo J, et al. Novel carbon dots derived from DryopteridisCrassirhizomatis Rhizoma Carbonisatum andtheir hemostatic effect. Chinese Traditional and Herbal Drugs, 2019, 50(06): 1388-1394.

[36] Zhao J, Liu ZY. Advances in Chemical Constituents andPharmacological Activities of RhizomaAtractylodis. Anhui Chemical Industry, 2019, 45(01): 10-13.

[37] Wang G, Hao R, Luo C, Wang Y, Man S, Gao W. Pharmacokinetics profiles of polyphyllin II and polyphyllin VII in rats by liquid chromatography with tandem mass spectrometry. Biomed Chromatogr, 2021, 35(6): e5083. doi:10.1002/bmc.5083.

[38] Ștefănescu R, Tero-Vescan A, Negroiu A, Aurică E, Vari C E. A Comprehensive Review of the Phytochemical, Pharmacological, and Toxicological Properties of Tribulus terrestris L. Biomolecules, 2020, 10(5). doi:10.3390/biom10050752.

[39] Gao M, Zhang TT, Qu MM, Yan JW, Ji JZ, Yu HB. Modern Study of Entada phaseoloides(Linn.)Merr. and Predictive Analysis on Q-markers. Modern Chinese Medicine, 2023, 25(05): 1125-1134. doi:10.13313/j.issn.1673-4890.20220905003.

[40] Zeng Z, Sun Z, Wu C Y, Long F, Shen H, Zhou J, et al. Quality evaluation of Pterocephali Herba through simultaneously quantifying 18 bioactive components by UPLC-TQ-MS/MS analysis. J Pharm Biomed Anal, 2024, 238: 115828. doi:10.1016/j.jpba.2023.115828.

[41] Man F, Choo C Y. HPLC-MS/MS method for bioavailability study of bruceines D & E in rat plasma. J Chromatogr B Analyt Technol Biomed Life Sci, 2017, 1063: 183-8. doi:10.1016/j.jchromb.2017.08.037.

[42] Li M C, Zhang Y Q, Meng C W, Gao J G, Xie C J, Liu J Y, et al. Traditional uses, phytochemistry, and pharmacology of Toxicodendron vernicifluum (Stokes) F.A. Barkley - A review. J Ethnopharmacol, 2021, 267: 113476. doi:10.1016/j.jep.2020.113476.

[43] Li X, Wang W, Hu ZP, Lu TX, Lu X, Wang DJ. Comparison of Anti-inflammatory andAnalgesic Effects of Berberis soulieana withDifferent Processing Methods and Optimizationof Processing Technology. Journal of Chinese Medicinal Materials, 2019, 42(12): 2797-2802. doi:10.13863/j.issn1001-4454.2019.12.012.

[44] Li B, Duan SL, Wang SC, Zeng N, Yao QY, Yao W, et al. Research progress on chemical constituents andpharmacological activities of Pseudolarixamabilis. Chinese Traditional and Herbal Drugs, 2021, 52(19): 6096-6110.

[45] Zhao FH, Hou XT, Hao EW, Liang YF, Du ZC, Deng JG. Chemical Constituents,Toxicology andPharmacological Effect of Momordicae Semen. Chinese Journal of Experimental Traditional Medical Formulae, 2020, 26(03): 222-235. doi:10.13422/j.cnki.syfjx.20192307.

[46] Zeng F F, Chen Z H, Luo F H, Liu C J, Yang X, Zhang F X, et al. Sophorae tonkinensis radix et rhizoma: A comprehensive review of the ethnopharmacology, phytochemistry, pharmacology, pharmacokinetics, toxicology and detoxification strategy. J Ethnopharmacol, 2025, 337(Pt 1): 118784. doi:10.1016/j.jep.2024.118784.

[47] Su F, Sun Y, Zhu W, Bai C, Zhang W, Luo Y, et al. A comprehensive review of research progress on the genus Arisaema: Botany, uses, phytochemistry, pharmacology, toxicity and pharmacokinetics. J Ethnopharmacol, 2022, 285: 114798. doi:10.1016/j.jep.2021.114798.

[48] Wang HP, Yu HL, Wu H, Xie YW, Tao XB, Zeng p, et al. Effect of Arisaematis Rhizoma Processed on theContents of Lectin Protein and Calcium OxalateCrystal as Toxic Components. Journal of Nanjing University of Traditional Chinese Medicine, 2022, 38(05): 375-381. doi:10.14148/j.issn.1672-0482.2022.0375.

[49] Ma L, Meng XH, Yang JL. Research progress on chemicalconstituents,pharmacological activities andclinical application of Euphorbia kansui. Natural Product Research and Development, 2022, 34(04): 699-712. doi:10.16333/j.1001-6880.2022.4.018.

[50] Cruz L S, de Oliveira T L, Kanunfre C C, Paludo K S, Minozzo B R, Prestes A P, et al. Pharmacokinetics and cytotoxic study of euphol from Euphorbia umbellata (Bruyns) Pax latex. Phytomedicine, 2018, 47: 105-12. doi:10.1016/j.phymed.2018.04.055.

[51] Zhang JY. Research Progress of Laggera pterodonta (DC.) Benth. Pharmacy Information, 2022. doi:10.12677/pi.2022.115047.

[52] Wang PH, Wang YZ, Wang HN, Yang ZY, Zhang GY, Chen MY, et al. Study on excretion kinetics of three mainditerpene esters in rats of Euphorbiae Semenbefore and after frosting based on UPLC.MS/MS technique. Chinese Journal of Pharmaceutical Analysis, 2021, 41(11): 1904-1913. doi:10.16155/j.0254-1793.2021.11.07.

[53] Zhu A, Zhang T, Wang Q. The phytochemistry, pharmacokinetics, pharmacology and toxicity of Euphorbia semen. J Ethnopharmacol, 2018, 227: 41-55. doi:10.1016/j.jep.2018.08.024.

[54] Tao Y, Su D, Li W, Cai B. Pharmacokinetic comparisons of six components from raw and vinegar-processed Daphne genkwa aqueous extracts following oral administration in rats by employing UHPLC-MS/MS approaches. J Chromatogr B Analyt Technol Biomed Life Sci, 2018, 1079: 34-40. doi:10.1016/j.jchromb.2018.02.005.

[55] Yuan T T, Xu H T, Zhao L, Lv L, He Y J, Zhang N D, et al. Pharmacokinetic and tissue distribution profile of curculigoside after oral and intravenously injection administration in rats by liquid chromatography-mass spectrometry. Fitoterapia, 2015, 101: 64-72. doi:10.1016/j.fitote.2014.12.012.

[56] Wu D, Wang H, Tan J, Wang C, Lin H, Zhu H, et al. Pharmacokinetic and Metabolism Studies of Curculigoside C by UPLC-MS/MS and UPLC-QTOF-MS. Molecules, 2018, 24(1). doi:10.3390/molecules24010021.

[57] Lu Y, Yang D, Song X, Wang S, Song M, Hang T. Bioaccessibility and health risk assessment of mercury in cinnabar containing Traditional Chinese Medicines. J Trace Elem Med Biol, 2017, 44: 17-25. doi:10.1016/j.jtemb.2017.05.006.

[58] Chen ZY, Zhang Y. Research progress in the cardiovascular toxicityof 15 toxic Chinese materia medica. Chinese Journal of New Drugs, 2021, 30(14): 1282-1288.

[59] Zhao N J, Wang L L, Liu Z Y, Wang Q, Liu L, Sun Z L, et al. Pharmacokinetics of chelerythrine and its metabolite after oral and intramuscular administrations in pigs. Xenobiotica, 2021, 51(11): 1264-1270. doi:10.1080/00498254.2021.1882714.

[60] Qian Y, Su S, Wei M, Zhu Z, Guo S, Yan H, et al. Interactions of pharmacokinetic profiles of Ginkgotoxin and Ginkgolic acids in rat plasma after oral administration. J Pharm Biomed Anal, 2019, 163: 88-94. doi:10.1016/j.jpba.2018.09.053.

[61] Lv Z, Li C, Wu T, Zhao P, Liu Y, Ouyang H, et al. Development of a high sensitivity UHPLC-MS/MS method to determine the twelve compounds of Physochlainae Radix extract and application to a pharmacokinetic study in rats. Arabian Journal of Chemistry, 2024, 17(4): 105664. doi:https:// doi.org/10.1016/j.arabjc.2024.105664.

[62] Wang MK, Wang MW, Li MG, Wei YD, Song CG, Chen TC. Research on Correlation between EffectiveComponents and Color Values ofBaifuzi(Typhonii Rhizoma) Based on AHP.entropy Weight Method. Chinese Archives of Traditional Chinese Medicine, 2022, 40(12): 125-131+296. doi:10.13193/j.issn.1673-7717.2022.12.025.

[63] Lu W, Hu L, Yang J, Sun X, Yan H, Liu J, et al. Isolation and pharmacological characterization of a new cytotoxic L-amino acid oxidase from Bungarus multicinctus snake venom. J Ethnopharmacol, 2018, 213: 311-320. doi:10.1016/j.jep.2017.11.026.

[64] Zhou YL, Yang P, Li XX, Li XF. Research progress on chemical constituents andpharmacological effects of Pinelliae Rhizomaand its quality marker prediction analysis. Chinese Traditional and Herbal Drugs, 2024, 55(14): 4939-4952.

[65] Fan W, Fan L, Peng C, Zhang Q, Wang L, Li L, et al. Traditional Uses, Botany, Phytochemistry, Pharmacology, Pharmacokinetics and Toxicology of Xanthium strumarium L.: A Review. Molecules, 2019, 24(2). doi:10.3390/molecules24020359.

[66] Liu Y, Liu L, Tian CK, Zhou DZ. Research progress of studies on chemicalconstituents and biologic activities of Anemonespecies. China Journal of Chinese Materia Medica, 2019, 44(05): 912-919. doi:10.19540/j.cnki.cjcmm.20181226.011.

[67] Song YJ, Zou HC, Weng X, Ju JH. Research progress on chemical constituents andpharmacological activities of small moleculecompounds in scorpions. China Journal of Chinese Materia Medica, 2024, 49(03): 661-670. doi:10.19540/j.cnki.cjcmm.20231212.201.

[68] Jiang J, Li P, Zhou HP, Qian YY, Sun L, Wan R, et al. Study condition and the quality control studyroute of the sulfur fumigation chinese medicine. China Journal of Traditional Chinese Medicine and Pharmacy, 2014, 29(06): 1764-1767.

[69] Zhao B, Cheng M, Xue YB, Zhang JH, Zhang ZH, Pan XB, et al. Research progress on chemical constituents,pharmacological activities and clinicalapplication of Melia Cortex. West China Journal of Pharmaceutical Sciences, 2023, 38(02): 227-235. doi:10.13375/j.cnki.wcjps.2023.02.023.

[70] Xiao Q, Xu KX, Zhang WT, Wang YT, Huang WZ, Liu J, et al. Overview on chemical compounds andpharmacological effects of radix euphorbiaelantu. Global Traditional Chinese Medicine, 2017, 10(08): 1027-1032.

[71] Wei W L, Li H J, Yang W Z, Qu H, Li Z W, Yao C L, et al. An integrated strategy for comprehensive characterization of metabolites and metabolic profiles of bufadienolides from Venenum Bufonis in rats. J Pharm Anal, 2022, 12(1): 136-144. doi:10.1016/j.jpha.2021.02.003.

[72] Gao P, Wang L, Chen Y, Yang X, Chen X, Yue C, et al. Pharbitidis Semen: A review of botany, traditional uses, phytochemistry, pharmacology, and toxicology. J Ethnopharmacol, 2023, 314: 116634. doi:10.1016/j.jep.2023.116634.

[73] Li W, Wang M, Mi HY, Zhang P, Gao HY, Wei F, et al. Research Progress on Processing TechnologyChemical Constituents and Pharmacology ofEuphorbia pekinensis. Chinese Pharmaceutical Journal, 2024, 59(02): 111-117.

[74] Sun WX, Sheng YH, Huang J, Sun J, Wei ST, Tang LM. Study on pharmacodynamics andpharmacokinetics of Phytolacca acinosa Roxb.and its mechanism. Chinese Pharmacological Bulletin, 2022, 38(09): 1429-1434.

[75] Lin F, Wang R, Du J, Wen C, Wang X, Jin Y, et al. Pharmacokinetics and bioavailability of febrifugine in rat plasma determined by UPLC-MS/MS. Acta Chromatographica, 2025, 37(3): 388-393. doi:https:// doi.org/10.1556/1326.2024.01288.

[76] Wang S, Yu X, Wu S, Yang W, Gao Y, Wang W, et al. Simultaneous determination of periplocin, periplocymarin, periplogenin, periplocoside M and periplocoside N of Cortex Periplocae in rat plasma and its application to a pharmacokinetic study. Biomed Chromatogr, 2022, 36(3): e5283. doi:10.1002/bmc.5283.

[77] Zhu JL, Deng YJ, He YS, Wang R, Kuang HX, Wang QH. Research Progress on chemicalConstituents,Pharmacological Effect and ClinicalApplications of Daturae Flos. Chinese Journal of Experimental Traditional Medical Formulae, 2021, 27(23): 201-209. doi:10.13422/j.cnki.syfjx.20211549.

[78] Zhang QL, Qu Q, Xie YY, Shen LS, Fan MW, Hu P, et al. Toxicokinetics study of arsenic in Liushen pillsand realgar. Journal of Toxicology, 2011, 25(05): 332-5. doi:10.16421/j.cnki.1002-3127.2011.05.014.

[79] Fu JT, Zhang Y, Zhang WP, Guo L, Xu B, Xie JW. Toxicokinetics of castor bean extraction afterintragastrical administration in SD rats andmeasurement of clinical intoxcant samples. Chinese Journal of Pharmacology and Toxicology, 2022, 36(08): 599-604.

[80] Li X, Zhang MT, Guo XX, Wang JD, Li DH, Ma X. Study on change of Morphine Content inYingsugiao(Papaveris Pericarpium) Before andAfter Processing. Liaoning Journal of Traditional Chinese Medicine, 2023, 50(12): 174-178. doi:10.13192/j.issn.1000-1719.2023.12.048.

[81] Yang C, Zhang B, Chen CJ, Liu PA, Peng YM, Tan QY, et al. Research Progress of Deinagkistrodon Acutus Venom. Chinese Journal of Modern Applied Pharmacy, 2023, 40(23): 3324-3333. doi:10.13748/j.cnki.issn1007-7693.20230099.

[82] Hu Y, Gao B, Zhang H, Guo ZJ. Research Progress on Processing HistoryEvolution, Chemical Constituents andPharmacological Action of Centipede. Acta Chinese Medicine and Pharmacology, 2024, 52(06): 98-104. doi:10.19664/j.cnki.1002-2392.240125.

[83] Lin A, Su X, She D, Qiu K, He Q, Liu Y. LC-MS/MS determination and comparative pharmacokinetics of strychnine, brucine and their metabolites in rat plasma after intragastric administration of each monomer and the total alkaloids from Semen Strychni. J Chromatogr B Analyt Technol Biomed Life Sci, 2016, 1008: 65-73. doi:10.1016/j.jchromb.2015.11.012.

[84] Jain B, Jain N, Jain S, Teja P K, Chauthe S K, Jain A. Exploring brucine alkaloid: A comprehensive review on pharmacology, therapeutic applications, toxicity, extraction and purification techniques. Phytomedicine Plus, 2023, 3(4): 100490. doi:https:// doi.org/10.1016/j.phyplu.2023.100490.

[85] Yan P, Zhang L, Peng C, Zhang R. Pharmacokinetics and tissue distribution of crotonoside. Xenobiotica, 2018, 48(1): 28-36. doi:10.1080/00498254.2016.1276311.

[86] Dang YJ, Zhu CY. Pharmacokinetics and bioavailability of cantharidin in beagledogs. China Journal of Chinese Materia Medica, 2009, 34(16): 2088-2091

[87] Zhang XQ, Li T, Wang HY, Liu N, Guo XH. Effect of Compatibility of Gardeniae Fructus andRhododendri Mollis Flos on Pharmacokinetics ofRhodojaponin I and Rhodojaponin m in Rat Plasma. Chinese Journal of Modern Applied Pharmacy, 2023, 40(24): 3352-3359. doi:10.13748/j.cnki.issn1007-7693.20231377.

[88] Cheng Y, Chu Y, Su X, Zhang K, Zhang Y, Wang Z, et al. Pharmacokinetic-pharmacodynamic modeling to study the anti-dysmenorrhea effect of Guizhi Fuling capsule on primary dysmenorrhea rats. Phytomedicine, 2018, 48: 141-151.doi:10.1016/j.phymed.2018.04.041

[89] Guan HY, Wang Q, Mei Y, Ran JY, Zeng FL, Cai HM, et al. A multistep approach for exploring quality markers of Shengjiang Xiexin decoction by integrating plasma pharmacochemistry-pharmacokinetics-pharmacology. J Pharm Biomed Anal, 2024, 241: 115999.doi:10.1016/j.jpba.2024.115999

[90] Chen YQ, Fan XS, Zhou LP, Hu FY, Wang PL. Screening and evaluation of quality markers from Shuangshen Pingfei formula for idiopathic pulmonary fibrosis using network pharmacology and pharmacodynamic, phytochemical, and pharmacokinetic analyses. Phytomedicine, 2022, 100: 154040.doi:10.1016/j.phymed.2022.154040

[91] Gao X, Du X, An LJ, Wang YY, Wang LJ, Wu ZG, et al. Wilforine, the Q-marker and PK-maker of Tripterygium glycosides tablet: Based on preparation quantitative analysis and PK-PD study. Phytomedicine, 2019, 54: 357-364.doi:10.1016/j.phymed.2018.03.031

[92] Wang Q, Chen GT, Chen XT, Liu YH, Qin ZF, Lin P, et al. Development of a three-step-based novel strategy integrating DMPK with network pharmacology and bioactivity evaluation for the discovery of Q-markers of traditional Chinese medicine prescriptions: Danlou tablet as an example. Phytomedicine, 2023, 108: 154511.doi:10.1016/j.phymed.2022.154511

[93] Wang TY, Lin S, Li H, Liu R, Liu ZH, Xu HR, et al. A stepwise integrated multi-system to screen quality markers of Chinese classic prescription Qingzao Jiufei decoction on the treatment of acute lung injury by combining 'network pharmacology-metabolomics-PK/PD modeling'. Phytomedicine, 2020, 78: 153313.doi:10.1016/j.phymed.2020.153313

[94] Wang XR, Lu JZ, Li GT, Luo LF, Yuan Z, Li M, et al. Established UPLC-MS/MS procedure for multicomponent quantitative analysis of rat plasma: Pharmacokinetics of Taohong Siwu Decoction in normal and acute blood stasis models. J Ethnopharmacol, 2023, 305: 116094.doi:10.1016/j.jep.2022.116094

[95] Zhang FG, Zhang Y, Li XF, Zhang SQ, Zhu MD, Du WX, et al. Research on Q-markers of Qiliqiangxin capsule for chronic heart failure treatment based on pharmacokinetics and pharmacodynamics association. Phytomedicine, 2018, 44: 220-230.doi:10.1016/j.phymed.2018.03.003

[96] Wei CL, Wu LJ, Wu YY, Xu CY, Hu HL, Wang ZG. Selection and evaluation of quality markers (Q-markers) of vladimiriae radix extract for cholestatic liver injury based on spectrum-effect relationship, pharmacokinetics, and molecular docking. J Ethnopharmacol, 2024, 329: 118151.doi:10.1016/j.jep.2024.118151

[97] Wang XY, Xie YJ, Bayoude A, Zhang BL, Yu BY. Discovering the Q-marker of scutellaria baicalensis against viral pneumonia integrated chemical profile identification, pharmacokinetic, metabolomics and network pharmacology. J Ethnopharmacol, 2025, 340: 119232.doi:10.1016/j.jep.2024.119232
